# Blood RNA biomarkers for tuberculosis screening in people living with HIV before antiretroviral therapy initiation: a diagnostic accuracy study

**DOI:** 10.1016/S2214-109X(24)00029-9

**Published:** 2024-04-04

**Authors:** Tiffeney Mann, Rishi K Gupta, Byron W P Reeve, Gcobisa Ndlangalavu, Aneesh Chandran, Amirtha P Krishna, Claire J Calderwood, Happy Tshivhula, Zaida Palmer, Selisha Naidoo, Desiree L Mbu, Grant Theron, Mahdad Noursadeghi

**Affiliations:** Division of Infection and Immunity and Institute of Health Informatics University College London, London, UK; Division of Infection and Immunity and Institute of Health Informatics University College London, London, UK; DSI-NRF Centre of Excellence for Biomedical Tuberculosis Research, South African Medical Research Council Centre for Tuberculosis Research, Division of Molecular Biology and Human Genetics, Faculty of Medicine and Health Sciences, Stellenbosch University, Cape Town, South Africa; DSI-NRF Centre of Excellence for Biomedical Tuberculosis Research, South African Medical Research Council Centre for Tuberculosis Research, Division of Molecular Biology and Human Genetics, Faculty of Medicine and Health Sciences, Stellenbosch University, Cape Town, South Africa; Division of Infection and Immunity and Institute of Health Informatics University College London, London, UK; Division of Infection and Immunity and Institute of Health Informatics University College London, London, UK; Department of Clinical Research, Faculty of Infectious and Tropical Diseases, London School of Hygiene & Tropical Medicine, London, UK; DSI-NRF Centre of Excellence for Biomedical Tuberculosis Research, South African Medical Research Council Centre for Tuberculosis Research, Division of Molecular Biology and Human Genetics, Faculty of Medicine and Health Sciences, Stellenbosch University, Cape Town, South Africa; DSI-NRF Centre of Excellence for Biomedical Tuberculosis Research, South African Medical Research Council Centre for Tuberculosis Research, Division of Molecular Biology and Human Genetics, Faculty of Medicine and Health Sciences, Stellenbosch University, Cape Town, South Africa; DSI-NRF Centre of Excellence for Biomedical Tuberculosis Research, South African Medical Research Council Centre for Tuberculosis Research, Division of Molecular Biology and Human Genetics, Faculty of Medicine and Health Sciences, Stellenbosch University, Cape Town, South Africa; DSI-NRF Centre of Excellence for Biomedical Tuberculosis Research, South African Medical Research Council Centre for Tuberculosis Research, Division of Molecular Biology and Human Genetics, Faculty of Medicine and Health Sciences, Stellenbosch University, Cape Town, South Africa; DSI-NRF Centre of Excellence for Biomedical Tuberculosis Research, South African Medical Research Council Centre for Tuberculosis Research, Division of Molecular Biology and Human Genetics, Faculty of Medicine and Health Sciences, Stellenbosch University, Cape Town, South Africa; Division of Infection and Immunity and Institute of Health Informatics University College London, London, UK

## Abstract

**Background:**

Undiagnosed tuberculosis remains a major threat for people living with HIV. Multiple blood transcriptomic biomarkers have shown promise for tuberculosis diagnosis. We sought to evaluate their diagnostic accuracy and clinical utility for systematic pre-antiretroviral therapy (ART) tuberculosis screening.

**Methods:**

We enrolled consecutive adults (age ≥18 years) referred to start ART at a community health centre in Cape Town, South Africa, irrespective of symptoms. Sputa were obtained (using induction if required) for two liquid cultures. Whole-blood RNA samples underwent transcriptional profiling using a custom Nanostring gene panel. We measured the diagnostic accuracy of seven candidate RNA signatures (one single gene biomarker [BATF2] and six multigene biomarkers) for the reference standard of *Mycobacterium tuberculosis* culture status, using area under the receiver-operating characteristic curve (AUROC) analysis, and sensitivity and specificity at prespecified thresholds (two standard scores above the mean of healthy controls; Z2). Clinical utility was assessed by calculating net benefit in decision curve analysis. We compared performance with C-reactive protein (CRP; threshold ≥5 mg/L), WHO four-symptom screen (W4SS), and the WHO target product profile for tuberculosis triage tests.

**Findings:**

A total of 707 people living with HIV (407 [58%] female and 300 [42%] male) were included, with median CD4 count 306 cells per mm^3^ (IQR 184–486). Of 676 participants with available sputum culture results, 89 (13%) had culture-confirmed tuberculosis. The seven RNA signatures were moderately to highly correlated (Spearman rank coefficients 0·42–0·93) and discriminated tuberculosis culture positivity with similar AUROCs (0·73–0·80), but none statistically better than CRP (AUROC 0·78, 95% CI 0·72–0·83). Diagnostic accuracy was similar across CD4 count strata, but lower among participants with negative W4SS (AUROCs 0·56–0·65) compared with positive (AUROCs 0·75–0·84). The RNA biomarker with the highest AUROC point estimate was a four-gene signature (Suliman4; AUROC 0·80, 95% CI 0·75–0·86), with sensitivity 83% (95% CI 74–90) and specificity 59% (55–63) at the Z2 threshold. In decision curve analysis, Suliman4 and CRP had similar clinical utility to guide confirmatory tuberculosis testing, but both had higher net benefit than W4SS. In exploratory analyses, an approach combining CRP (≥5 mg/L) and Suliman4 (≥Z2) had sensitivity of 80% (70–87), specificity of 70% (66–74), and higher net benefit than either biomarker alone.

**Interpretation:**

RNA biomarkers showed better clinical utility to guide confirmatory tuberculosis testing for people living with HIV before ART initiation than symptom-based screening, but their performance did not exceed that of CRP and fell short of WHO recommended targets. Interferon-independent approaches might be required to improve accuracy of host-response biomarkers to support tuberculosis screening before ART initiation.

**Funding:**

South African Medical Research Council, European and Developing Countries Clinical Trials Partnership 2, National Institutes of Health National Institute of Allergy and Infectious Diseases, The Wellcome Trust, National Institute for Health and Care Research, Royal College of Physicians London.

## Introduction

Tuberculosis continues to be a leading cause of death among people living with HIV and often remains undiagnosed.^1,2^ Prompt diagnosis and treatment initiation are required to reduce mortality in HIV-associated tuberculosis (HIV-TB). However, diagnosis can be challenging, with only an estimated 56% of people with incident HIV-TB notified in 2019.^3^ Therefore, WHO recommends systematic screening for tuberculosis among people living with HIV at every health-care encounter.^3^ Since 2011, systematic screening has been based on the WHO four-symptom screen (W4SS) to trigger confirmatory tuberculosis testing using culture or molecular assays, and investigate for tuberculosis before initiation of preventive treatment.^3^ W4SS has an estimated 84% sensitivity and 37% specificity for tuberculosis among outpatients not receiving antiretroviral therapy (ART).^4^ More recently, WHO have also endorsed measurement of blood C-reactive protein (CRP) of at least 5 mg/L for screening, which showed similar sensitivity (89%) but higher specificity (54%) than W4SS in an individual participant data meta-analysis.^3,4^ However, this still falls short of the WHO target product profile of at least 90% sensitivity and 70% specificity,^5^ necessitating the development of novel approaches with greater accuracy than W4SS or CRP.^3,4^

Multiple blood transcriptomic biomarkers for tuberculosis have shown promise for prediction of incident tuberculosis over an interval of 3–6 months^6^ and for diagnosis of prevalent tuberculosis among people who are symptomatic.^7–9^ Head-to-head analyses have shown areas under the receiver-operating characteristic curves (AUROCs) of 0·87–0·91 for the best performing signatures to identify culture-positive tuberculosis among symptomatic people self-presenting to health-care services, irrespective of HIV status.^7^ In the context of general population screening, transcriptomic signature AUROCs for prevalent tuberculosis have been estimated as 0·63–0·79 in people without HIV and 0·65–0·88 among people living with HIV, with inferior performance among people who are asymptomatic.^8,10^ Signatures are now being translated to near-patient assays, for example using the GeneXpert platform, with promising initial performance among individuals who are symptomatic.^11^ However, data among people living with HIV are scarce, with only ten prevalent tuberculosis cases in the only previous general population screening study of people living with HIV.^12^ Moreover, the vast majority of people living with HIV in previous studies of both passive and active case finding were receiving ART. There are insufficient data from untreated people living with HIV.

Pre-ART initiation is a key timepoint for tuberculosis screening, because this frequently represents a nadir of immunocompromise associated with elevated risk of tuberculosis both before and during the initial months of ART.^13^ Untreated HIV might also be associated with upregulation of interferon signalling^12,14,15^ that could reduce the specificity of blood transcriptomic biomarkers for tuberculosis, as is evident during intercurrent respiratory viral infections.^16,17^ In this study, we sought to evaluate the diagnostic accuracy and clinical utility as measured by net benefit in decision curve analysis, for a range of concise candidate blood transcriptomic signatures of tuberculosis in a large cohort of people living with HIV, in South Africa, before commencement of ART. We benchmarked performance against the WHO target product profile, along with CRP and W4SS as alternative screening approaches.

## Methods

### Study participants

**Figure 1:**
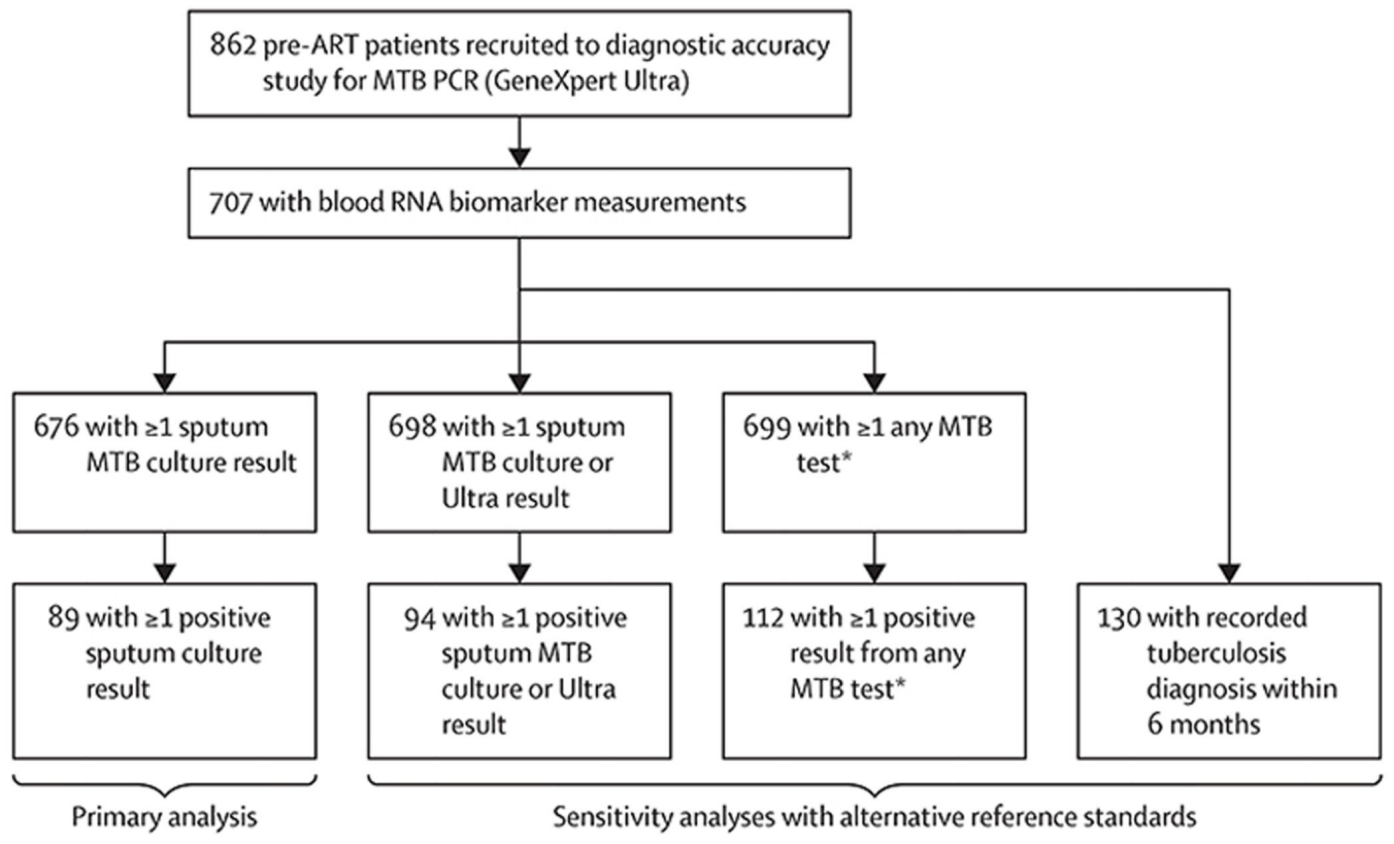
CONSORT diagram for patient recruitment and outcomes Schematic representation of number of eligible and recruited patients and their outcomes for primary and secondary analyses. ART=antiretroviral therapy. MTB=*Mycobacterium tuberculosis*. *Any MTB test includes microbiological tests on urine as well as sputum samples.

This study was nested within a diagnostic accuracy study of sputum Xpert MTB/RIF Ultra (Cepheid, Sunnydale, CA, USA) in which consecutive adults with HIV infection referred to start ART at Kraaifontein Community Health Centre in Cape Town, South Africa, were prospectively enrolled irrespective of symptoms of tuberculosis.^18^ This study cohort is generally representative of southern African populations with hyperendemic transmission of tuberculosis and HIV. Participants were excluded if they were younger than 18 years, had received any tuberculosis treatment within the last 2 months, were of unknown treatment status, or did not consent. Extrapulmonary tuberculosis was not excluded. In the current study, we included participants who were recruited between May 15, 2017, and Dec 14, 2020, who had blood RNA and CRP data available (figure 1). This study was approved by the Stellenbosch University Faculty of Health Sciences Research Ethics Committee (N14/10/136) and the Western Cape Department of Health, South Africa (WC_2016RP38_944), and is registered on ClinicalTrials. gov, NCT03187964. All participants provided written informed consent. The study is reported in line with STARD guidance.^19^

Age, self-reported (male or female) sex, comorbidity, and symptom and tuberculosis treatment data were captured at baseline. CRP was measured in real time using a point-of-care assay (iChromaII platform, Boditech, Chuncheon, South Korea), or a retrospective laboratory assay using stored serum when point-of-care data were unavailable (six participants). Three sputum samples were obtained per participant; two underwent smear microscopy and liquid culture, and the third sample was tested using Ultra. Expectoration was attempted for at least one sample. If this was not sufficient, sputum induction was performed using nebulised hypertonic saline. Blood RNA was collected in Tempus tubes (Thermo Fisher Scientific, Waltham, MA, USA) and preserved at –80°C. Urine samples were collected and tested using Ultra and Determine lateral flow lipoarabinomannan assay (Abbott, Johannesburg, South Africa). Tuberculosis diagnoses and treatment data within 6 months of enrolment were obtained through linkage to routinely collected health record data held at the Western Cape Provincial Health Data Centre using a deterministic algorithm.^20^

The reference standard for primary analyses was sputum liquid culture status for *Mycobacterium tuberculosis* complex. Secondary reference standards included: sputum culture or Ultra positivity (excluding trace positive results); any positive tuberculosis test, including urine lipoarabinomannan assay or Ultra (excluding sputum Ultra trace positive results); and recorded tuberculosis diagnosis or tuberculosis treatment in study or linkage data within 6 months of enrolment. Reference standard assessments were blinded to index test results. Participants with missing outcome data were excluded for each analysis. Participants with missing RNA or CRP data were excluded from all analyses. These exclusions were primarily due to either: missing blood RNA samples, because the substudy started with a short delay after the initiation of the parent study; brief interruptions to supply of blood RNA collection (Tempus) tubes; or 10% technical failure rate in RNA extractions.

Our sample size calculation was based on achieving a minimum AUROC of 0·8, equivalent to that of CRP^4^ with a 95% lower CI bound of 0·75, requiring 700 participants with a minimum tuberculosis prevalence rate of 10% (appendix 1 p 10).^21^

### Sample processing and Nanostring

Sample processing for blood RNA signatures was performed blinded to the reference standard sputum culture results. Peripheral blood RNA samples were extracted using the Tempus Spin RNA Isolation kit (Thermo Fisher Scientific) and the Turbo DNA-free Kit (Invitrogen, Waltham, MA, USA). RNA integrity scores were determined using the Agilent Tape Station (Agilent, Santa Clara, CA, USA). Transcriptional profiling of 300 ng of blood RNA was performed using a custom gene panel on the Nanostring platform (Nanostring Technologies, Seattle, WA, USA). The gene panel was designed to include 23 genes encompassing seven candidate RNA signatures for tuberculosis, limited to concise signatures of up to 11 genes that performed well in our previous head-to-head analyses (appendix 2).^6,7^ The signatures included were Suliman4, RISK6, Sweeney3, Giddon3, BATF2, Roe3 and Zak11. Signatures are referred to with a prefix of the first author’s surname from the original publication where the signature was derived, and a suffix of the number of component genes, with the exceptions of BATF2 (a single transcript) and RISK6 (as named by the original investigators).^22^

Nanostring data were analysed on the nCounter Analyser (Nanostring Technologies), as per manufacturer’s instructions. Quality control and gene expression normalisation were performed with nSolver Analysis software (version 4.0.70). Gene expression values were log_2_-transformed and then normalised by subtracting the log_2_ expression of a housekeeping gene (*GAPDH*). Blood RNA signature Z scores were calculated by standardising scores for each signature to the mean and SD of blood samples (n=105) from a previously reported healthy control population of individuals with latent tuberculosis,^23^ also measured using the same Nanostring codeset. This group comprised a more ethnically diverse population than our cohort of people living with HIV, but had similar distributions of age and sex.

Reference RNA samples (Universal Human Reference RNA, Agilent) were included in each Nanostring run for quality control. These samples showed minimal coefficients of variation for each gene, supporting reproducibility of measurements (appendix 1 p 11). We also examined the discrimination of tuberculosis and non-tuberculosis cases using Nanostring measurements of the seven candidate RNA signatures in 59 paired RNA samples from our previously reported presumptive tuberculosis cohort,^7^ to ensure that we could reproduce the results derived from RNAseq data (appendix 1 p 12).

Principal component analysis revealed systematic differences in reference RNA data by manufacturing codeset batch (appendix 1 p 13). Therefore, we performed batch correction by codeset manufacturing batch, using the ComBat function from the sva package in R.^24^ Distributions of target genes differed by batch to a varying degree for each probe before batch correction (appendix 1 p 14). These differences resolved after correction (appendix 1 pp 15–16).

### Data analysis

Analyses were conducted in R (version 4.0.2). Blood RNA signature scores were calculated from processed Nanostring data as reported previously (appendix 1 p 3).^6,7^ For the Roe3 signature, we sought to simplify our approach to calculating signature scores to promote easier use of this signature. We showed that geometric mean calculation for the three component genes perform as well as the original support vector machine approach that was used to derive this signature^1^ (appendix 1 p 17). The simplified geometric mean calculation was therefore used in all subsequent analyses.

We quantified discrimination for each biomarker by constructing receiver-operating characteristic curves and calculating AUROCs using the pROC package, with 95% CIs using the DeLong method.^25^ We also compared AUROCs for each RNA signature with CRP using paired DeLong tests, with adjustment for multiple testing using a Benjamini–Hochberg correction.^25^ Sensitivities, specificities, and predictive values for each RNA signature were calculated using prespecified cutoffs of two standard scores above the mean of the healthy control population (Z2).

Subgroup analyses were performed by stratifying by W4SS status (presence of any of fever, cough, weight loss, or sweats) and CD4 count (<200 cells per mm^3^ *vs* ≥200 cells per mm^3^). We also compared discrimination between participants with a recorded tuberculosis diagnosis or treatment within 6 months of enrolment and those who remained free of tuberculosis for this period, stratified by sputum culture, to assess if accuracy varied according to sputum culture status. We compared discrimination for each signature between subgroups using unpaired DeLong tests, with adjustment for multiple testing as before.

Correlation between RNA signatures, CRP, and physiological indices of disease severity were examined using scatter plots and Spearman rank correlation coefficients. We also examined factors associated with higher RNA signature scores using multivariable linear regression, with restricted cubic splines for continuous variables to account for potential non-linear associations. In order to investigate the clinical utility of candidate tuberculosis screening strategies, decision curve analysis was performed using the rmda package,^26^ as described previously.^27^ Briefly, decision curve analysis determines the net benefit of diagnostic approaches, in comparison with intervening for all or no participants.^28^ Net benefit reflects the true positive rate minus false positive rate weighted by the cost–benefit ratio across a range of threshold probabilities that will trigger a decision, as a surrogate measure of the range of cost–benefit ratios. As a result, the false positive penalty increases and net benefit reduces with increasing cost–benefit ratio. The intervention in the context of a triage test for tuberculosis is the offer of confirmatory testing (eg, using sputum culture). The threshold probability reflects the minimum probability of disease at which further investigation would be triggered. Accordingly, this threshold represents the number willing to test for every case of tuberculosis detected. Hence, a threshold probability of 0·1 indicates that a positive predictive value of at least 10% is enough to trigger confirmatory testing, and reflects a number willing to test of 10 per true case detected. Cost is not limited to economic resources, but rather encompasses any risk of the intervention. As perceived cost–benefit ratio of confirmatory testing increases, so does the threshold probability that will trigger confirmatory testing and a reduction in the number willing to test. The ideal approach has the highest net benefit across a clinically relevant threshold probability range. The net benefit of using the best performing RNA biomarker (at a threshold of Z2) to guide confirmatory testing was compared with alternative strategies of confirmatory testing for all, confirmatory testing for none, and confirmatory testing guided by CRP (cutoff ≥5 mg/L as recommended in WHO guidance^3,4^) or W4SS.

We also performed a range of exploratory analyses. First, we examined whether an optimised HIV-TB RNA signature could be derived by temporally splitting the cohort into a development set (505 [75%] patients, with 56 culture positive for *M tuberculosis*) and validation set (171 [25%] patients, with 33 culture positive for *M tuberculosis*). In this analysis, temporal splitting of the cohort was favoured to randomly selected subsets because it tests whether the training model is robust despite time-dependent variables, thus providing greater confidence of generalisability compared with a random split. We then ranked the 23 measured transcripts by AUROC for tuberculosis culture status in the development set and examined whether iteratively adding genes improved discrimination in the held-out validation set. We used a range of approaches to combine individual genes to calculate overall scores, including simple calculations (geometric means or disease risk scores^29^) and multivariable models trained on the development set (logistic regression and support vector machines). Second, we used the same development versus validation split to explore whether a multivariable model including the most discriminating RNA signature, CRP, and clinical predictors (number of W4SS symptoms, haemoglobin, CD4 count, and BMI) could further improve performance, compared with the RNA biomarker alone. These predictors were measured at baseline and selected for inclusion in the multivariable model on the basis of previous analyses showing associations with HIV-TB risk.^2^ For this analysis, we used a multivariable logistic regression approach for the primary outcome of tuberculosis culture status, with restricted cubic splines to model potential non-linear associations. We quantified discrimination in the temporal validation set as the AUROC. Finally, we examined whether an approach combining CRP (≥5 mg/L) and the most discriminating RNA biomarker (Z score ≥2) could offer better net benefit to guide confirmatory testing in decision curve analysis.

#### Sensitivity analyses

We explored the effect of alternative reference standard definitions, as described above, in sensitivity analyses. We also examined an alternative approach to Nanostring data batch correction by normalising probe-level data to the mean of the reference RNA samples for each batch.

#### Role of the funding source

The funders had no role in study design, data collection, data analysis, data interpretation, writing of the report, or the decision to submit for publication.

## Results

A total of 862 participants were recruited to the parent study during the study period. Of these, 707 (82%) had blood RNA and CRP data available and were included in the analysis (figure 1, table 1). There were no systematic differences between included participants and those excluded due to the absence of RNA or CRP data (appendix 1 pp 4–5). Of the included study population (n=707), median age was 32 years (IQR 27–39), 407 (58%) were female and 300 (42%) male, and median CD4 cell count was 306 cells per mm^3^ (184–486). 406 (57%) participants presented with at least one of the symptoms comprising the W4SS, and 633 (90%) had two sputum culture results available. Of 676 participants with at least one sputum culture result, 89 (13%) had positive results, and 65 (9%) of the 699 with available Ultra results had positive results. 130 (18%) of 707 participants had a recorded tuberculosis diagnosis or treatment within 6 months of enrolment, representing the sum of prevalent cases at enrolment and incident cases within 6 months identified by linkage to registry data. 109 (84%) of these cases were within 4 weeks and 116 (89%) within 8 weeks (appendix 1 p 18). For 11 (10%) of 107 participants with known site of disease, the site was extrapulmonary.

**Figure 2:**
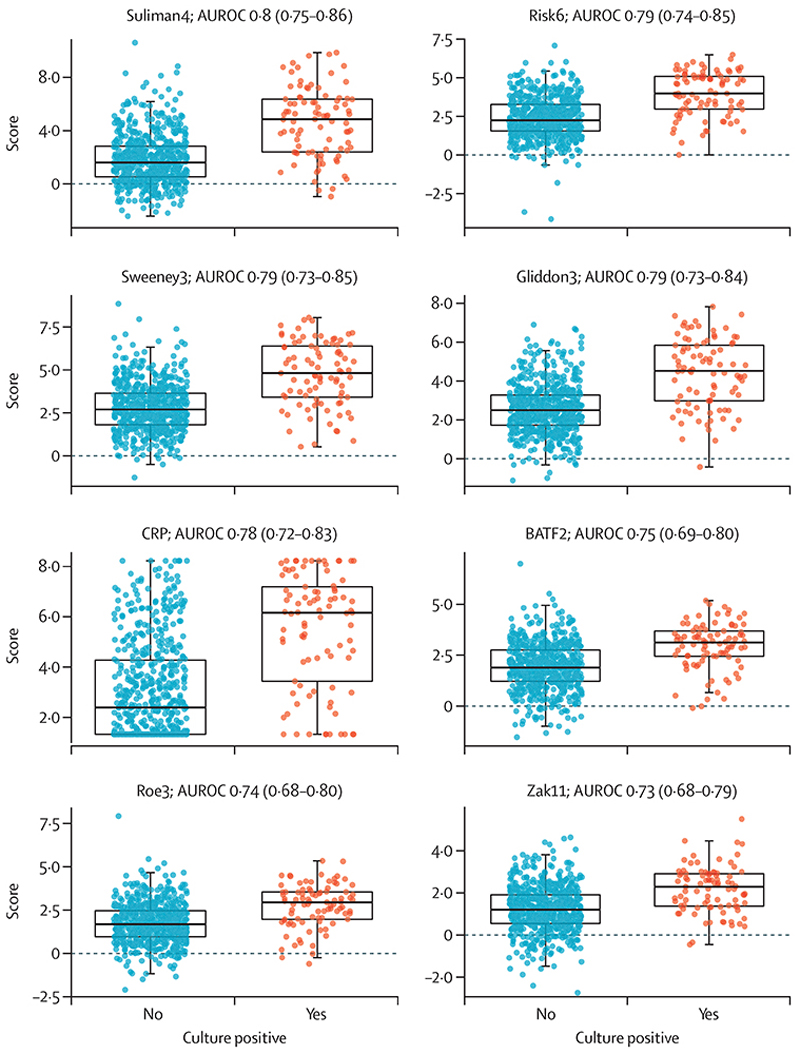
Blood RNA biomarker discrimination of *Mycobacterium tuberculosis* sputum culture positivity Scores and discrimination of RNA signatures (Z scores) and CRP (log2-transformed, mg/L) for the primary outcome of sputum culture positivity (n=676). Discrimination is presented as AUROCs with 95% CIs. AUROC=area under the receiver-operating characteristic curve. CRP=C-reactive protein.

The seven RNA biomarkers had similar discrimination for sputum culture status, with AUROCs ranging from 0·73 (95% CI 0·68–0·79) for Zak11 to 0·80 (0·75–0·86) for Suliman4 (figure 2). All RNA biomarkers had statistically equivalent discrimination to CRP in pairwise tests (CRP AUROC 0·78 [0·72–0·83]; table 2). Using Z2 cutoffs, none of the signatures met the WHO-recommended minimum sensitivity of 90% and specificity of 70% for a triage test (table 2). Suliman4, the RNA signature with the highest AUROC point estimate, had sensitivity of 83% (74–90) and specificity of 59% (55–63). Most signatures had higher sensitivity than specificity at Z2 cutoffs, with 28–74% of participants having a score above the Z2 threshold. In view of the relationship of tuberculosis blood RNA signatures with interferon signalling,^6^ the poor specificity is consistent with active interferon signalling in untreated HIV infection.^12,14,15^ Positive predictive values ranged from 16% to 27%, and negative predictive values from 92% to 97%. In comparison, CRP had sensitivity of 85% (77–91) and specificity of 47% (44–52) at the primary cutoff of at least 5 mg/L, with 56% of participants having a positive result. Alternative CRP cutoffs of at least 8 mg/L and at least 10 mg/L are presented in appendix 1 (p 6), with slightly lower sensitivity and higher specificity at the higher cutoffs, but not meeting the WHO targets. For Suliman4 at the Z2 threshold, the numbers needed to test with confirmatory testing were 4·3 (3·5–5·3) among positive and 23·9 (14·8–39·2) among negative participants. For CRP, numbers needed to test were similar at 5·0 (4·1–6·2) among positive and 22·7 (13·5–38·6) among negative individuals.

In subgroup analyses, RNA signatures and CRP were less discriminating among W4SS-negative (AUROCs 0·56–0·65) compared with W4SS-positive participants (AUROCs 0·75–0·84; table 3). There were no differences in discrimination when stratified by CD4 count or sputum culture status of tuberculosis cases.

The seven RNA biomarkers were moderately to highly correlated (Spearman rank coefficients 0·42–0·93; appendix 1 p 19). Correlation was also observed between CRP and RNA biomarkers (Spearman rank coefficients 0·23–0·61), although this tended to be weaker than that observed between the RNA biomarkers. To examine whether RNA signature scores were associated with HIV-TB severity, we plotted scatter plots for Suliman4 (the signature with the highest AUROC point estimate) with age, self-reported sex, and clinical measures of disease severity, stratified by tuberculosis status. Higher Suliman4 scores were associated with lower BMI, CD4 count, haemoglobin, and middle upper arm circumference, along with higher TBscoreII,^30^ number of symptoms, and respiratory rate among participants with and without tuberculosis (appendix 1 p 20). Among those with tuberculosis, higher Suliman4 scores were associated with higher smear grade and lower time to positivity in liquid culture. In multivariable linear regression, number of symptoms, BMI, CD4 count, haemoglobin, respiratory rate, and sputum culture status were independently associated with higher Suliman4 scores (appendix 1 pp 7, 21).

**Figure 3:**
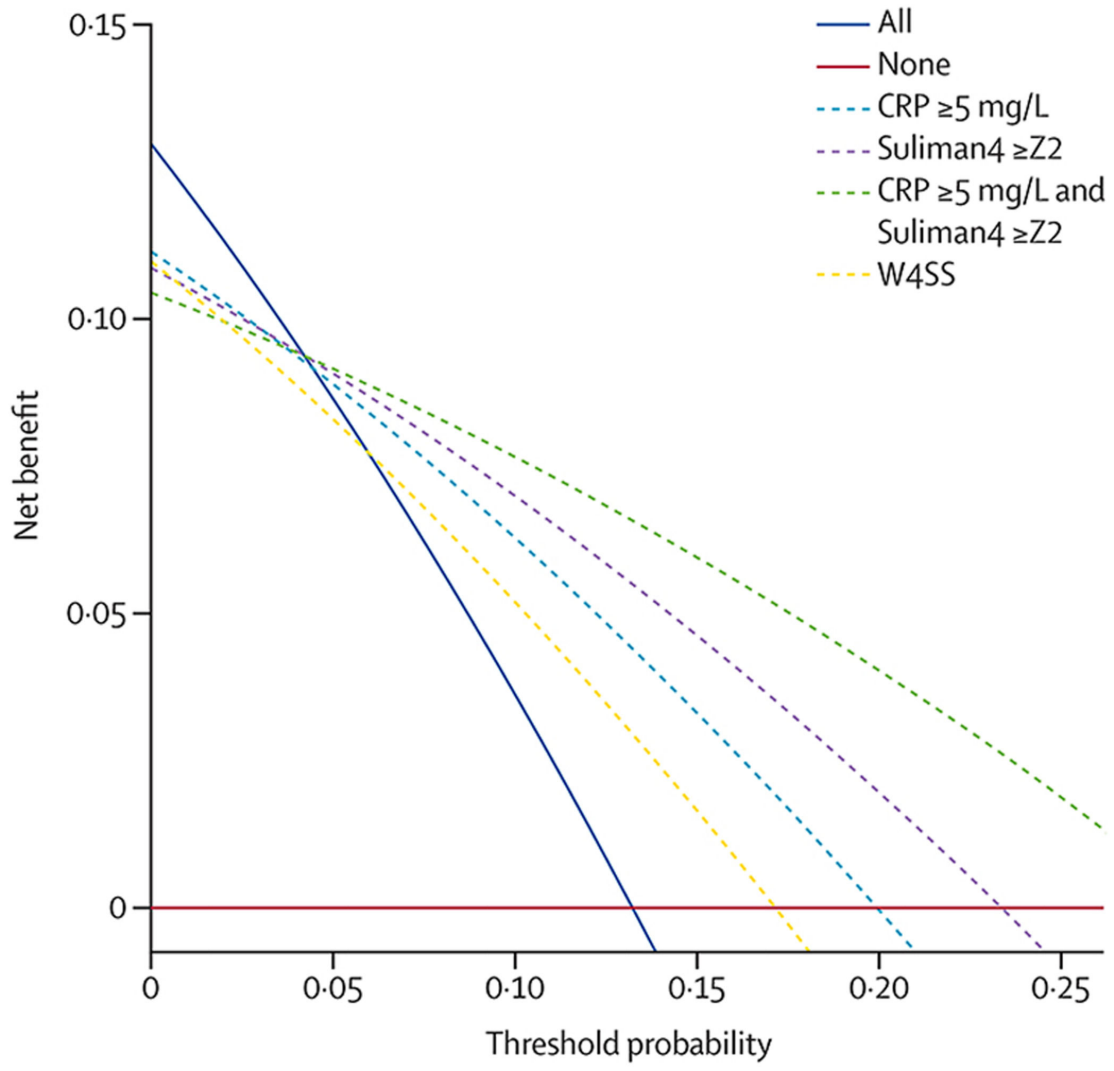
Decision curve analysis for alternative triage strategies to trigger confirmatory investigations for tuberculosis Net benefit (true positive rate minus weighted false positive rate) for different approaches to confirmatory testing for tuberculosis. Confirmatory testing for all and confirmatory testing for none approaches across the range of threshold probabilities that a service might use to trigger confirmatory investigations for tuberculosis are compared with those of decisions to investigate triggered by each of the triage strategies indicated: CRP (≥5 and ≥10 mg/L), Suliman4 (Z2), symptoms (W4SS), and using an exploratory approach of CRP of at least 5 mg/L and Suliman4 of at least Z2. CRP=C-reactive protein. W4SS=WHO four-symptom screen.

In decision curve analysis, Suliman4 with a Z2 cutoff to guide confirmatory testing had higher net benefit than an approach of confirmatory testing for all when the threshold probability exceeded approximately 4% (equivalent to a number willing to test with confirmatory testing of up to around 24 people per true tuberculosis case detected; figure 3, appendix 1 p 8). Using CRP of at least 5 mg/L had similar, albeit slightly lower, net benefit to Suliman4. There was a larger incremental net benefit for Suliman4 with increasing threshold probabilities (ie, when the number willing to test was lower). Both Suliman4 and CRP had higher net benefit than W4SS, which itself surpassed a confirmatory testing for all approach above threshold probabilities of approximately 6% (equivalent to a number willing to test with confirmatory testing of up to around 15 people per true tuberculosis case detected).

Our forward search to identify an optimised RNA signature for HIV-TB did not lead to significantly improved discrimination for tuberculosis culture status in the temporal validation set, when using simple calculations or multivariable models to combine individual gene expression values (appendix 1 pp 9, 22). A multivariable model trained on the development set incorporating Suliman4, CRP, and clinical predictors (number of W4SS symptoms, haemoglobin, CD4 count, and BMI) also did not lead to significant improvement in discrimination for the primary reference standard, with AUROC 0·81 (0·71–0·91) in the validation set. Our exploratory approach of combining CRP (≥5 mg/L) and Suliman4 (≥Z2) had sensitivity of 80% (70–87) and specificity of 70% (66–74), positive predictive value 29% (24–35), and negative predictive value 96% (93–97), with 36% (33–40) of the cohort being triage positive. In decision curve analyses, this combined approach had slightly higher net benefit than Suliman4 alone, with an incrementally greater net benefit at higher threshold probabilities (figure 3).

Our sensitivity analyses using alternative reference standard definitions for tuberculosis and an alternative approach to Nanostring data batch correction did not lead to any substantial difference in the main results (appendix 1 pp 24–26).

## Discussion

To our knowledge, this is the first study to examine the diagnostic accuracy and clinical utility of a range of promising blood RNA signatures for the application of systematic HIV-TB screening before ART initiation. We found that the seven candidate signatures had similar diagnostic accuracy for culture-positive tuberculosis and were moderately to highly correlated to each other. This is consistent with our previous findings suggesting the signatures are regulated by the same immune response pathways,^6^ and highlights that this approach offers multiple biomarker options that would be expected to have similar performance. However, none of the candidate signatures met the WHO target product profile criteria for a triage test and performance did not exceed that of CRP alone. The RNA signature with the highest point estimate was Suliman4; both Suliman4 and CRP had superior clinical utility to guide confirmatory testing compared with W4SS. Signature accuracy appeared independent of CD4 count and the sputum culture status of tuberculosis cases. However, accuracy was lower among W4SS-negative participants for all signatures and CRP, suggesting inferior performance in the absence of symptomatic disease.

Although most RNA signatures either met or approached the WHO triage test sensitivity target of 90% at the Z2 cutoff, specificity was generally below the 70% target. This inadequate specificity might reflect upregulation of interferon activity that we have previously shown underpins expression of tuberculosis biomarker genes.^6^ Such upregulation of interferon activity could be driven by untreated HIV^12,14,15^ or other opportunistic infections. Notably, higher RNA signature scores were associated with indices of HIV severity (including lower BMI, haemoglobin, and CD4 count) in univariable and multivariable analyses, further supporting this hypothesis by demonstrating higher scores among more unwell participants. Collectively, these findings highlight the need to develop interferon-independent host-response biomarkers to improve the specificity of tuberculosis screening among people living with HIV.

In our clinical utility analyses, we showed that both Suliman4 and CRP had higher net benefit than confirmatory testing for all, if the health service is willing to perform up to approximately 24 confirmatory tests per true tuberculosis case diagnosed. If the health service can perform more confirmatory tests than this, then our analysis suggests a confirmatory testing for all approach might be preferable. Of note, our exploratory approach combining CRP (≥5 mg/L) and Suliman4 (≥Z2) showed higher net benefit than either biomarker alone, with largely preserved sensitivity of 80% and improved specificity of 70%. This finding reflects the observation that blood RNA biomarkers and CRP had weak to moderate correlation, and might, therefore, provide orthogonal information. Combining both biomarkers has the potential to improve specificity and, consequently, clinical utility. Such a combinatorial approach would require further independent validation to assess whether it might provide a pathway to achieve the WHO target product profile for triage or potentially confirmatory tests, which is yet to be met by any single biomarker. Even if test specificity is substantially improved, the need for antimicrobial susceptibility profiling might restrict the application of host-response biomarkers to triage tests, requiring confirmatory microbiology. In addition, technological advances in near-patient or point-of-care testing platforms and substantial reductions in costs will be necessary to achieve the WHO target for a rapid and accessible triage test costing less than US$2. Because CRP testing is already widely available, including low-cost point-of-care platforms, our data support the programmatic roll-out of CRP for pre-ART tuberculosis screening^3^ while better biomarkers are sought.

Our study has numerous strengths, including the size of the cohort, with a representative sample of 707 participants newly referred to initiate ART. The cohort were well characterised and intensively investigated for tuberculosis, with 90% having two sputum culture results available, thus enabling a robust culture-based primary reference standard as recommended by the WHO target product profile for evaluation of triage tests,^3^ and complemented by sputum induction when required. In addition, sputum Ultra and urine diagnostics were systematically applied, and data linkage was performed to routinely collected data warehouse records to identify participants who were diagnosed and treated for tuberculosis following study enrolment. Limitations in accurate matching and completeness of the registry are recognised.^20^ Nonetheless, the results of our sensitivity analyses using alternative reference standards supported the robustness of our primary findings. We implemented a laboratory and analysis pipeline using the Nanostring platform to measure seven candidate RNA signatures that have performed well in previous analyses.^6–8^ The signatures were curated through our previous systematic review^6^ and were reproduced according to the original authors’ descriptions. The Nanostring pipeline showed high levels of reproducibility and our head-to-head analysis showed superior discrimination for tuberculosis status when compared with RNA sequencing data in a subset of samples from our previously published presumptive tuberculosis cohort, thus reinforcing its robustness. In addition, we used a healthy control population at risk of tuberculosis (with evidence of latent infection) to establish normal reference ranges and biomarker cutoffs at a prespecified threshold based on two SDs above the mean of controls. Finally, CRP was also measured, enabling direct head-to-head analyses with the candidate RNA biomarkers.

Our study is limited to a single centre, precluding assessments of generalisability across settings. This setting could be considered to be generally representative of southern African populations with hyperendemic transmission of tuberculosis and HIV. Further studies in diverse settings are required to assess wider generalisability, but the low specificity of the biomarkers means that the positive predictive value of these tests will be reduced among people living with HIV with lower previous probability of tuberculosis. Our targeted approach to RNA quantification precludes development of novel signatures beyond the 23 measured transcripts. Further genome-wide discovery will be required in such cohorts to identify novel biomarkers. Viral load data were not available and, therefore, we were unable to test the hypothesis that high viral loads are independently associated with higher RNA signature scores. This limitation was mitigated by the availability of multiple other indices of HIV severity, which were associated with RNA signature scores, but failed to improve the performance of biomarkers in multivariable models.

In conclusion, RNA biomarkers showed better clinical utility to guide confirmatory tuberculosis testing for pre-ART screening than W4SS, but their performance did not exceed that of CRP and fell short of WHO-mandated targets. Interferon-independent approaches for host-response tuberculosis diagnostic screening might be required to improve specificity among people living with HIV before ART initiation. Until then, the clinical and health-economic impact of widely available point-of-care CRP tests should be further evaluated for pre-ART tuberculosis screening.

## Supplementary Material

Supplementary appendix 1

Supplementary appendix 2

Supplementary appendix 3

## Figures and Tables

**Table 1:** Baseline characteristics of the included study cohort, stratified by culture status

	Overall (N=707)	Negative (n=587)	Positive (n=89)	Missing (n=31)
Age, years	32 (27–39)	32 (26–39)	35 (29–43)	33 (26–39)
Sex				
Female	407 (58%)	356 (61%)	35 (39%)	16 (52%)
Male	299 (42%)	230 (39%)	54(61%)	15 (48%)
Missing	1	1	0	0
Previous tuberculosis	98 (14%)	82 (14%)	13 (15%)	3 (10%)
CD4, cells/mm^3^	306 (184–486)	333 (206–511)	204 (66–314)	262 (81–478)
Missing	5	2	2	1
CD4 <200 cells/mm^3^	193 (27%)	139 (24%)	42 (47%)	12 (39%)
Missing	5	2	2	1
Haemoglobin, g/dL	12·0 (11·30–13·60)	12·95 (11·50–14·10)	11·35 (9·80–12·57)	12·60 (11·55–13·75)
Missing	150	127	19	4
BMI, kg/m^2^	24 (21–29)	25 (21–30)	21(19–23)	24 (22–27)
Missing	2	1	1	0
Middle upper arm circumference, cm	27·0 (25·0–30·0)	28·0 (25·0–31·0)	25·0 (23·0–27·0)	27·0 (25·0–29·2)
WHO four-symptom screen positive	406 (57%)	311 (53%)	70 (79%)	25 (81%)
TBscorell	1·00 (0·00–2·00)	1·00 (0·00–1·00)	2·00 (1·00–3·00)	1·00 (0·00–2·00)
Missing	36	26	9	1
C-reactive protein (mg/L)	6 (2–32)	5 (2–19)	71 (11–146)	10 (3–52)
Number of valid sputum cultures				
0	31 (4%)	0	0	31 (100%)
1	43 (6%)	39 (7%)	4 (4%)	0
2	633 (90%)	548 (93%)	85 (96%)	0
Sputum smear	21 (3%)	7 (1%)	13 (15%)	1 (3%)
Missing	13	0	0	13
Sputum Ultra				
Negative	616 (88%)	572 (98%)	23 (26%)	21 (88%)
Trace	18 (3%)	10 (2%)	6 (7%)	2 (8%)
Positive	65 (9%)	4 (<1%)	60(67%)	1 (4%)
Missing	8	1	0	7
Urine lipoarabinomannan assay	18 (3%)	6 (1%)	10 (11%)	2 (6%)
Missing	3	3	0	0
Urine Ultra	37 (5%)	11 (2%)	24 (27%)	2 (6%)
Missing	6	5	1	0
Sputum culture or Ultra positive	94 (13%)	4 (<1%)	89 (100%)	1 (3%)
Missing	9	0	0	9
Any positive tuberculosis test	112 (16%)	21 (4%)	89 (100%)	2 (6%)
Missing	8	0	0	8
Recorded tuberculosis diagnosis or treatment within 6 months	130 (18%)	37 (6%)	85 (96%)	8 (26%)
Tuberculosis site				
Extrapulmonary tuberculosis	11 (10%)	9 (29%)	1 (1%)	1 (14%)
Pulmonary tuberculosis	96 (90%)	22 (71%)	68 (99%)	6 (86%)
Missing	600	556	20	24

Data are median (IQR), n, or n (%).

**Table 2:** Blood RNA biomarker and CRP performance metrics for discrimination of sputum culture status

	AUROC	Sensitivity	Specificity	Positive predictive value	Negative predictive value	Triage positive	NNT(+)	NNT(–)	p value
Suliman4	0·80 (0·75–0·86)	83% (74–90)	59% (55–63)	23% (19–28)	96% (93–97)	47% (43–51)	4·3 (3·5–5·3)	23·9 (14·8–39·2)	0·32
Risk6	0·79 (0·74–0·85)	91% (83–95)	39% (35–43)	18% (15–22)	97% (94–98)	65% (61–68)	5·4 (4·5–6·6)	29·8 (15·4–58·4)	0·58
Sweeney3	0·79 (0·73–0·85)	92% (85–96)	28% (25–32)	16% (13–20)	96% (92–98)	74% (71–77)	6·1 (5·0–7·5)	24·9 (12·4–51·0)	0·61
Gliddon3	0·79 (0·73–0·84)	89% (81–94)	31% (28–35)	16% (13–20)	95% (91–97)	71% (68–75)	6·1 (5·0–7·5)	19·4 (10·8–35·4)	0·61
CRP	0·78 (0·72–0·83)	85% (77–91)	48% (44–52)	20% (16–24)	96% (93–97)	56% (53–60)	5·0 (4·1–6·2)	22·7 (13·5–38·6)	··
BATF2	0·75 (0·69–0·8)	82% (73–89)	53% (49–57)	21% (17–25)	95% (92–97)	52% (48–56)	4·8 (3·9–5·9)	20·4 (12·8–32·9)	0·41
Roe3	0·74 (0·68–0·8)	73% (63–81)	61% (57–65)	22% (18–27)	94% (91–96)	44% (40–48)	4·6 (37–5·7)	15·8 (10·8–23·4)	0·32
Zak11	0·73 (0·68–0·79)	56% (46–66)	77% (73–80)	27% (21–34)	92% (89–94)	28% (24–31)	3·7 (3·0–4·8)	*12·6* (9·3–17·0)	0·32
W4SS	0·63 (0·58–0·68)	79% (69–86)	47% (43–51)	18% (15–23)	94% (90–96)	56% (53–60)	5·4 (4·4–6·8)	15·5 (10·2–24·0)	<0·0001

Data are AUROC (95% CI), % (95% CI), NNT (95% CI), or p value. p values indicate pairwise comparisons to CRP, with multiple testing correction (n=676). AUROC=area under the receiver-operating characteristic curve. CRP=C-reactive protein. NNT=number needed to test. W4SS=WHO four-symptom screen.

**Table 3:** Blood RNA biomarker and CRP performance metrics for discrimination of tuberculosis status, stratified by selected subgroups

	Discrimination		p value
W4SS	Positive	Negative	··
BATF2	0·78 (0·72–0·84)	0·59 (0·43–0·74)	0·029
CRP	0·81 (0·76–0·87)	0·56 (0.42–0·70)	0·0031
Gliddon3	0·83 (0·77–0·89)	0·58 (0.44–0·72)	0·0031
Risk6	0·83 (0·78–0·89)	0·59 (0·46–0·72)	0·0031
Roe3	0·77 (0·71–0·83)	0·57 (0·41–0·74)	0·029
Suliman4	0·84 (0·79–0·89)	0·6 (0·46–0·74)	0·0031
Sweeney3	0·84 (0·79–0·90)	0·56 (0·42–0·70)	0·0021
Zak11	0·75 (0·68–0·81)	0·65 (0·51–0·78)	0·20
CD4	<200 cells	≥200 cells	··
BATF2	0·76 (0·68–0·84)	0·70 (0.62–0·79)	0·48
CRP	0·84 (0·77–0·91)	0·70 (0·61–0·78)	0·11
Gliddon3	0·83 (0·75–0·9)	0·72 (0·63–0·81)	0·19
Risk6	0·83 (0·75–0·9)	0·73 (0·65–0·82)	0·19
Roe3	0·75 (0·66–0·83)	0·70 (0·61–0·79)	0·51
Suliman4	0·84 (0·76–0·92)	0·75 (0·67–0·83)	0·19
Sweeney3	0·84 (0·76–0·91)	0·73 (0·65–0·82)	0·19
Zak11	0·71 (0·62–0·8)	0·74 (0·65–0·82)	0·67
Tuberculosis sputum culture	Culture positive	Culture negative	··
BATF2	0·75 (0·7–0·81)	0·69 (0·61–0·77)	0·45
CRP	0·79 (0·73–0·85)	0·79 (0·72–0·86)	0·98
Gliddon3	0·8 (0·74–0·86)	0·76 (0·68–0·84)	0·55
Risk6	0·8 (0·75–0·86)	0·75 (0·67–0·83)	0·45
Roe3	0·75 (0·69–0·81)	0·69 (0·61–0·77)	0·45
Suliman4	0·82 (0·76–0·87)	0·79 (072–0·86)	0·69
Sweeney3	0·8 (0·74–0·85)	0·70 (0·61–0·78)	0·36
Zak11	0·74 (0·69–0·8)	0·67(0·60–0·75)	0·45

Accuracy shown for primary outcome of sputum culture status as area under the receiver-operating characteristic curve with 95% confidence intervals. Reference standard for W4SS and CD4 strata is sputum culture, as per primary reference standard. Reference standard for the sputum culture strata is based on tuberculosis diagnosis or treatment recorded within six months of enrolment. p values indicate comparisons between strata for each signature. CRP=C-reactive protein. W4SS=WHO four-symptom screen.

## Research in context

### Evidence before this study

Tuberculosis is a major cause of morbidity and mortality among people living with HIV, particularly among those with untreated HIV and consequent immunosuppression. Initiation of antiretroviral therapy (ART) for HIV is also associated with increased short-term risk of incident tuberculosis, attributed to immune reconstitution inflammatory syndrome, which could in turn potentiate the immunopathogenesis of tuberculosis. As a result, in settings of high tuberculosis prevalence, systematic screening for tuberculosis is widely advocated for people living with HIV before starting ART. In this context, universal sputum microbiological screening is not economically sustainable and is constrained by practical feasibility among those who are not expectorating sputum. Patient stratification to identify those at greater risk of tuberculosis is required to target resources for microbiological testing more precisely. WHO commissioned a systematic review and individual participant data meta-analysis of tuberculosis screening strategies among ambulatory people living with HIV, published in April, 2022. In this analysis, the WHO four-symptom screen (W4SS) showed an estimated 84% sensitivity and 37% specificity for pre-ART tuberculosis screening. Blood C-reactive protein (CRP) levels of at least 5 mg/L offered better performance, estimated at 89% sensitivity and 54% specificity, but still fell short of the WHO target product profile of at least 90% sensitivity and 70% specificity. Blood RNA biomarkers of tuberculosis, reflecting interferon and tumour necrosis factor-mediated immune responses, have been gaining momentum as potential triage tests for symptomatic and presymptomatic tuberculosis to enable targeted application of confirmatory microbiological testing. We performed a systematic search of PubMed (MEDLINE) indexed publications from database inception to Dec 5, 2023, using terms for tuberculosis, HIV, RNA, and biomarker without any language restriction. A number of blood RNA biomarker tuberculosis diagnostic accuracy studies have included symptomatic ART-naive people living with HIV, but none have previously evaluated their application for tuberculosis screening among people living with HIV initiating ART, or undertaken head-to-head analyses benchmarked against W4SS or CRP. Of note, untreated HIV also drives chronic interferon activity that might compromise the specificity of interferon-dependent biomarkers in this population.

### Added value of this study

To our knowledge, this is the largest study to date to benchmark the performance of candidate blood RNA biomarkers for unselected and systematic pre-ART tuberculosis screening among people living with HIV against contemporary standards and aspirational performance targets. The blood RNA biomarkers showed better diagnostic accuracy and clinical utility to guide confirmatory tuberculosis testing for people living with HIV than symptom-based screening with W4SS, but their performance did not exceed that of CRP, and they did not reach WHO recommended targets. The results were similar for microbiologically confirmed tuberculosis at enrolment to the study and for all people starting tuberculosis treatment within 6 months of enrolment. Blood RNA biomarkers correlated with features of disease severity that might be attributed to either tuberculosis or HIV. Accordingly, their discrimination of tuberculosis among people living with HIV was particularly limited by poor specificity. Diagnostic accuracy was statistically significantly better among people who were symptomatic compared with those who were asymptomatic, further limiting the value of RNA biomarkers in presymptomatic tuberculosis. Blood RNA biomarkers only showed moderate correlation with CRP, suggesting these two measurements provided information on different components of the host response. An exploratory analysis showed that CRP can be combined with the best performing blood RNA signature to provide better clinical utility than that achieved by either test alone.

### Implications of all the available evidence

Our data show that blood RNA biomarkers do not perform any better than CRP as triage tests for tuberculosis among people living with HIV before ART initiation. As CRP is already widely available on a low-cost, point-of-care platform, our findings support further evaluation of the clinical and health-economic impact of CRP-based triage for pre-ART tuberculosis screening. An underlying mechanism that limits the diagnostic accuracy of RNA biomarkers for tuberculosis among people living with HIV before ART could be upregulation of interferon signalling in untreated HIV. As interferon activity underpins upregulated expression of tuberculosis biomarker genes, HIV-induced upregulation of interferon-stimulated genes might reduce the specificity of blood transcriptomic biomarkers for tuberculosis in this context. These findings highlight a wider need to identify interferon-independent host-response-based biomarkers to support disease-specific screening of people living with HIV before ART initiation.

## Data sharing

Processed Nanostring data are provided in appendix 3, along with accompanying metadata at the time of peer-reviewed publication.
