# Supplementary material for: Blood RNA biomarkers for tuberculosis screening in people living with HIV before antiretroviral therapy initiation: a diagnostic accuracy study
Source: Lancet Glob Health. Author manuscript; Available in PMC 2026 Jul 21. (PMC7619259; doi:10.1016/S2214-109X(24)00029-9)
Supplement: Supplementary appendix 1 [file EMS196790-supplement-Supplementary_appendix_1.pdf]

THE LANCET
Global Health

Supplementary appendix 1

This appendix formed part of the original submission and has been peer reviewed. We post it as supplied by the authors.

Supplement to: Mann T, Gupta RK, Reeve BWP, et al. Blood RNA biomarkers for tuberculosis screening in people living with HIV before antiretroviral therapy initiation: a diagnostic accuracy study. *Lancet Glob Health* 2024; published online April 4. https://doi.org/10.1016/S2214-109X(24)00029-9.

Contents

Supplementary Table 1 3
Approach to calculation of blood RNA biomarker scores 3
Supplementary Table 2 4
Baseline characteristics of the study cohort, stratified by inclusion in the analyses 4
Supplementary Table 3 6
CRP performance metrics for discrimination of sputum culture positive and negative participants using alternative cut-offs. 6
Supplementary Table 4 7
Linear regression model showing factors associated with higher Suliman4 scores 7
Supplementary Table 5 8
Table showing thresholds where screening approaches have higher net benefit than confirmatory testing for all and none 8
Supplementary Table 6 9
Table showing greedy forward search looking for an optimised signature 9
Supplementary Figure 1 10
Sample size calculations 10
Supplementary Figure 2 11
Coefficients of variation for reference RNA samples, stratified by Nanostring codeset batch. 11
Supplementary Figure 3 12
Head to head comparison of the RNAseq and Nanostring quantitation of blood RNA signatures for identification of culture positive TB cases. 12
Supplementary Figure 4 13
Principal component analyses of reference RNA samples 13
Supplementary Figure 5 14
Distribution of gene expression values by Nanostring codeset batch 14
Supplementary Figure 6 15
Distribution of gene expression values by Nanostring codeset batch after COMBAT correction. 15
Supplementary Figure 7 16
Distribution of gene expression values by Nanostring codeset batch after reference RNA normalisation 16
Supplementary Figure 8 17
Discrimination of Roe3 and Zak11 signatures TB using simple geometric mean calculations. 17
Supplementary Figure 9 18
Prevalent in incident TB diagnoses by time from study enrolment. 18
Supplementary Figure 10 19
Supplementary Figure 11 20
Supplementary Figure 12 21
Linear regression multivariable model of predictors for Suliman4 blood RNA signature scores 21
Supplementary Figure 13 22

Greedy forward search for optimal signature to discriminate between PLHIV with and without culture positive TB. 22
Supplementary Figure 14 23
Sensitivity analysis: Sputum culture or Ultra positivity 23
Supplementary Figure 15 24
Sensitivity analysis: Any positive TB test 24
Supplementary Figure 16 25
Sensitivity analysis: Recorded TB diagnosis or treatment within 6 months 25
Supplementary Figure 17 26
Sensitivity analysis: Using reference RNA-normalised data 26
References 27

Supplementary Table 1

Approach to calculation of blood RNA biomarker scores

Signature	Calculation
BATF2[1]	BATF2
Gliddon3[2]	(FCGR1A + C1QB) - (ZNF296)
RISK6[3]	((GBP2 + FCGR1B + SERPING1)/3) - ((TUBGCP6 + TRMT2A + SDR39U1)/3))
Roe3[4]	(BATF2 + SCARF1 + GBP5)/3
Suliman4[5]	(GAS6 + SEPT4) - (CD1C + BLK)
Sweeney3[6]	(GBP5 + DUSP3)/2) - KLF2
Zak11[7]	Support vector machine model trained on original training dataset[8]

All calculations are performed on log-2 transformed data. A single Nanostring probe was used to measure FCGR1A and FCGR1B.

Supplementary Table 2

Baseline characteristics of the study cohort, stratified by inclusion in the analyses

Characteristic	Overall, N = 862	Included, N = 707[1]	Excluded, N = 155[1]	p-value[2]
Age (years)	32 (26, 39)	32 (27, 39)	32 (25, 38)	0.2
Sex				0.6
Female	501 (58%)	407 (58%)	94 (61%)	
Male	360 (42%)	299 (42%)	61 (39%)	
Missing	1	1	0	
Previous TB	124 (14%)	98 (14%)	26 (17%)	0.4
CD4 (cells/mm3)	299 (172, 486)	306 (184, 486)	274 (129, 490)	0.12
Missing	8	5	3	
CD4 <200 cells/mm3	248 (29%)	193 (27%)	55 (36%)	0.041
Missing	8	5	3	
Haemoglobin (g/dl)	12.70 (11.30, 13.90)	12.70 (11.30, 13.90)	12.50 (11.25, 13.83)	0.4
Missing	177	150	27	
Body mass index (kg/m2)	24 (21, 29)	24 (21, 29)	23 (20, 29)	0.4
Missing	2	2	0	
Middle upper arm circumference (cm)	27.0 (25.0, 30.0)	27.0 (25.0, 30.0)	27.0 (25.0, 30.5)	0.7
WHO 4-symptom screen positive	487 (56%)	406 (57%)	81 (52%)	0.3
TBscoreII	1.00 (0.00, 2.00)	1.00 (0.00, 2.00)	1.00 (0.00, 1.25)	0.6
Missing	39	36	3	
CRP (mg/L)	6 (2, 32)	6 (2, 32)	6 (2, 34)	0.6
Missing	17	0	17	
Number of valid sputum cultures				0.7
0	37 (4.3%)	31 (4.4%)	6 (3.9%)	
1	50 (5.8%)	43 (6.1%)	7 (4.5%)	
2	775 (90%)	633 (90%)	142 (92%)	
Sputum culture positive	107 (13%)	89 (13%)	18 (12%)	0.8
Missing	37	31	6	
Sputum Ultra				0.4
Negative	754 (89%)	616 (88%)	138 (91%)	
Trace	19 (2.2%)	18 (2.6%)	1 (0.7%)	
Positive	78 (9.2%)	65 (9.3%)	13 (8.6%)	
Missing	11	8	3	
Urine LAM	24 (2.8%)	18 (2.6%)	6 (3.9%)	0.4
Missing	5	3	2	
Urine Ultra	45 (5.3%)	37 (5.3%)	8 (5.3%)	>0.9
Missing	10	6	4	
Sputum culture or Ultra positive	113 (13%)	94 (13%)	19 (12%)	0.9
Missing	12	9	3	
Any positive TB test	137 (16%)	112 (16%)	25 (16%)	>0.9

Characteristic	Overall, N = 862	Included, N = 707[1]	Excluded, N = 155[1]	p-value[2]
Missing	11	8	3	
Recorded TB diagnosis or treatment within 6 months	152 (18%)	130 (18%)	22 (14%)	0.3

[1]Statistics presented: median (IQR); n (%)

[2]Statistical tests performed: Wilcoxon rank-sum test; chi-square test of independence; Fisher's exact test

Participants with missing CRP or RNA data were excluded.

Supplementary Table 3

CRP performance metrics for discrimination of sputum culture positive and negative participants using alternative cut-offs.

Test	Cut-off (mg/L)	Sensitivity	Specificity	PPV	NPV	Triage positive	NNT(+)	NNT(-)
CRP	10	0.75 (0.65 - 0.83)	0.64 (0.6 - 0.68)	0.24 (0.19 - 0.29)	0.94 (0.92 - 0.96)	0.41 (0.38 - 0.45)	4.2 (3.4 - 5.2)	18 (12.1 - 27)
CRP	8	0.79 (0.69 - 0.86)	0.59 (0.55 - 0.63)	0.23 (0.18 - 0.28)	0.95 (0.92 - 0.97)	0.46 (0.42 - 0.5)	4.4 (3.6 - 5.5)	19.3 (12.6 - 29.9)

Alternative cut-offs of 8mg/L and 10mg/L presented (n = 676 participants).

Supplementary Table 4

Linear regression model showing factors associated with higher Suliman4 scores

Characteristic	Beta	95% CI[1]	p-value
(Intercept)	3.8	2.2, 5.4	<0.0001
Age (years)	0.01	0.00, 0.03	0.2
Sex			
Female	—	—	
Male	0.13	-0.23, 0.49	0.5
CD4 count (cells/mm3; per 10 unit increase)	-0.02	-0.03, -0.02	<0.0001
Haemoglobin (g/dl)	-0.24	-0.32, -0.16	<0.0001
Body mass index (kg/m2)	-0.01	-0.04, 0.01	0.4
Respiratory rate (per min)	0.07	0.02, 0.11	0.004
Number of W4SS symptoms	0.40	0.26, 0.55	<0.0001
Sputum culture positive	1.5	1.0, 2.0	<0.0001

[1]CI = Confidence Interval

Supplementary Table 5

Table showing thresholds where screening approaches have higher net benefit than confirmatory testing for all and none

Score	Threshold probability range	NWT range
CRP >= 5mg/L & Suliman4 >= Z2	0.04-0.29	3.5-23.8
Suliman4 >= Z2	0.04-0.23	4.3-23.8
CRP >= 5mg/L	0.04-0.2	5-22.2
W4SS	0.06-0.18	5.5-15.4

Thresholds shown as threshold probabilities and as number willing to test (NWT) ranges with confirmatory tests per true TB case detected.

Supplementary Table 6

Table showing greedy forward search looking for an optimised signature

Number of genes	Added gene	Logistic regression	Support vector machine	Disease risk score	Difference in geometric means
1	SEPT4	0.77 (0.66-0.88)	0.77 (0.66-0.88)	0.77 (0.66-0.88)	0.77 (0.66-0.88)
2	FCGR1A	0.77 (0.66-0.87)	0.73 (0.63-0.83)	0.77 (0.66-0.87)	0.77 (0.66-0.87)
3	SERPING1	0.76 (0.66-0.87)	0.75 (0.65-0.85)	0.76 (0.65-0.86)	0.76 (0.65-0.86)
4	BATF2	0.76 (0.65-0.86)	0.75 (0.64-0.85)	0.75 (0.65-0.86)	0.75 (0.65-0.86)
5	CD1C	0.75 (0.63-0.87)	0.5 (0.4-0.61)	0.76 (0.65-0.87)	0.75 (0.64-0.86)
6	DUSP3	0.76 (0.64-0.87)	0.71 (0.59-0.83)	0.76 (0.65-0.87)	0.75 (0.63-0.86)
7	C1QB	0.75 (0.63-0.86)	0.73 (0.62-0.84)	0.76 (0.65-0.86)	0.75 (0.63-0.86)
8	KLF2	0.76 (0.65-0.87)	0.71 (0.6-0.82)	0.76 (0.65-0.87)	0.76 (0.65-0.87)
9	ZNF296	0.78 (0.67-0.88)	0.77 (0.67-0.87)	0.76 (0.65-0.87)	0.77 (0.66-0.88)
10	GBP1	0.78 (0.68-0.88)	0.75 (0.64-0.85)	0.76 (0.65-0.87)	0.77 (0.66-0.88)
11	GAS6	0.77 (0.67-0.88)	0.74 (0.63-0.86)	0.77 (0.66-0.88)	0.77 (0.66-0.88)
12	GBP5	0.8 (0.71-0.89)	0.76 (0.66-0.87)	0.77 (0.66-0.87)	0.77 (0.66-0.88)
13	SDR39U1	0.8 (0.71-0.89)	0.76 (0.66-0.86)	0.77 (0.66-0.88)	0.77 (0.66-0.89)
14	SCARF1	0.78 (0.69-0.88)	0.74 (0.64-0.85)	0.76 (0.65-0.87)	0.77 (0.66-0.88)
15	TRMT2A	0.79 (0.69-0.88)	0.74 (0.64-0.85)	0.77 (0.66-0.88)	0.77 (0.66-0.88)
16	ETV7	0.78 (0.68-0.88)	0.74 (0.63-0.85)	0.76 (0.65-0.87)	0.77 (0.66-0.88)
17	TAP1	0.78 (0.68-0.88)	0.74 (0.64-0.85)	0.76 (0.65-0.87)	0.77 (0.66-0.88)
18	GBP2	0.78 (0.68-0.88)	0.74 (0.63-0.84)	0.76 (0.65-0.87)	0.77 (0.66-0.88)
19	BLK	0.78 (0.68-0.87)	0.74 (0.64-0.85)	0.76 (0.65-0.87)	0.76 (0.65-0.87)
20	TUBGCP6	0.78 (0.68-0.87)	0.75 (0.64-0.85)	0.76 (0.65-0.87)	0.77 (0.66-0.88)
21	STAT1	0.77 (0.68-0.87)	0.74 (0.63-0.85)	0.76 (0.65-0.87)	0.77 (0.66-0.88)
22	TRAFD1	0.78 (0.69-0.88)	0.76 (0.66-0.86)	0.76 (0.65-0.87)	0.76 (0.65-0.87)

The full dataset was temporally split into 75%/25% development and validation sets. Increasing numbers of genes were iteratively added, in order of their discrimination for TB as single predictors.

Supplementary Figure 1

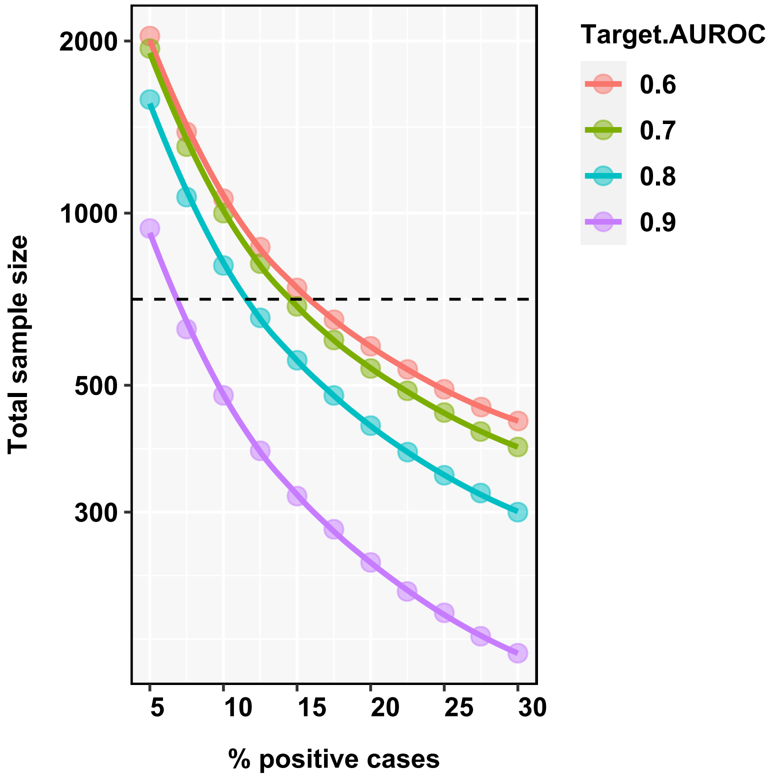

Sample size calculations

Relationship between total sample size required and proportion of positive cases within the sample stratified by target area under the receiver operating characteristic curve (AUROC) to discriminate between cases and controls with 95% lower bound confidence interval of 0.05 from target AUROC. Dashed line represents sample size of present study (N=707).

Supplementary Figure 2

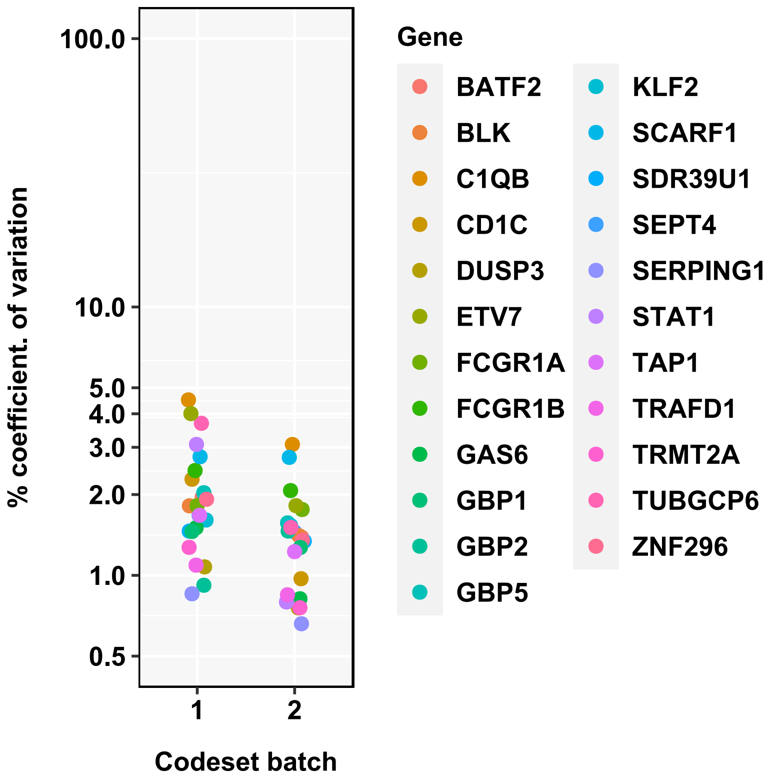

Coefficients of variation for reference RNA samples, stratified by Nanostring codeset batch.

The coefficients of variation are shown for repeated measurements (N=9, codeset batch 1, N=20 codeset batch 2) of target transcripts from a commercially derived human reference RNA sample.

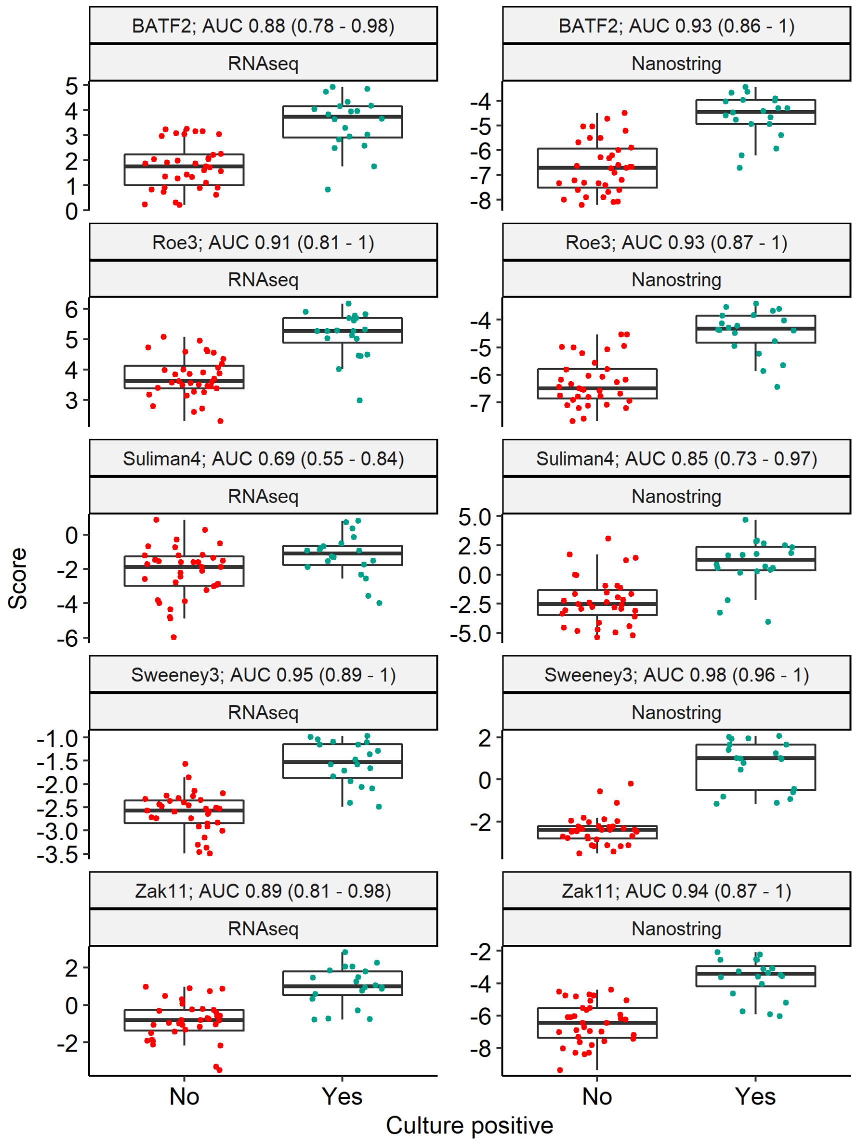

Supplementary Figure 3

Head to head comparison of the RNAseq and Nanostring quantitation of blood RNA signatures for identification of culture positive TB cases.

Discrimination of culture positive TB cases by Nanostring measurements (left panels) of seven candidate blood RNA signatures compared to that of previously reported[9] RNAseq results from a cohort of symptomatic individuals presented for evaluation (right panels) in 59 paired RNA samples. Data points show individual blood RNA signature scores by each method. Panel headings include area under the receiver operating characteristic curve and 95% confidence intervals for discrimination between Mtb culture positive and negative cases.

Supplementary Figure 4

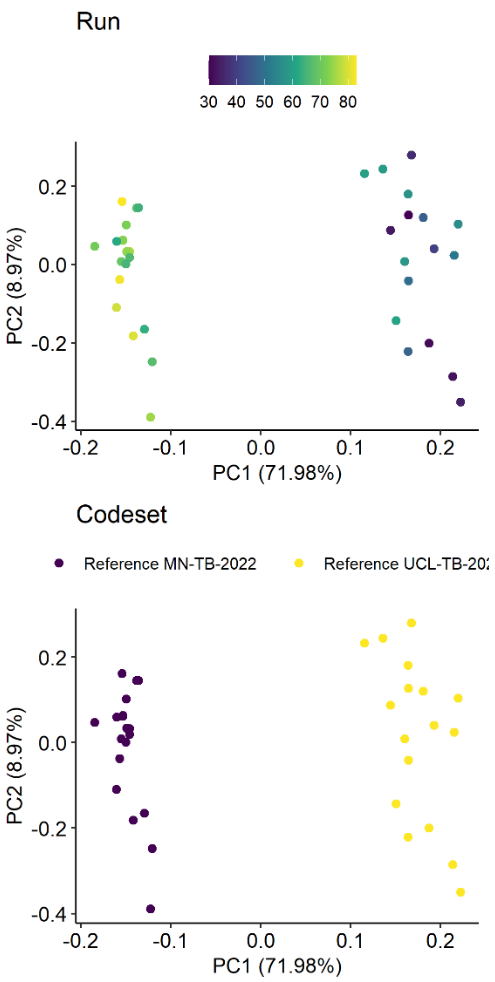

Principal component analyses of reference RNA samples

Principal component analysis of all gene level data derived from Nanostring measurements stratified by Nanostring run (top panel) and codeset batch used (bottom panel).

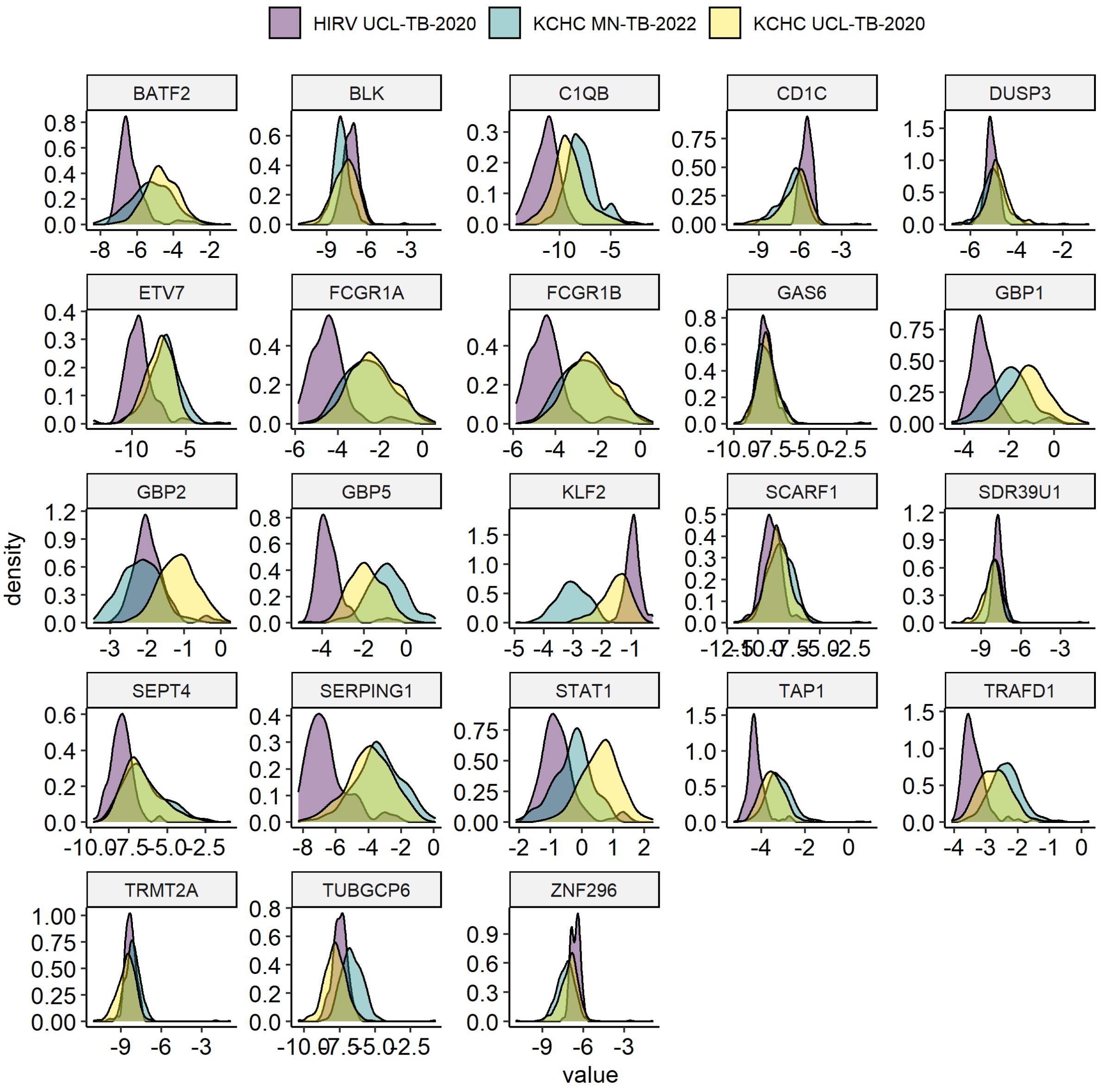

Supplementary Figure 5

Distribution of gene expression values by Nanostring codeset batch.

Density distributions of Nanostring gene expression data normalised to GAPDH, faceted by target gene, and subset by study group (prefix) and codeset batch (suffix). HIRV = healthy control population of patients with latent TB infection; KCHC= PLHIV study cohort; UCL-TB-2020 and MN-TB-2022 represent different codeset batches.

Supplementary Figure 6

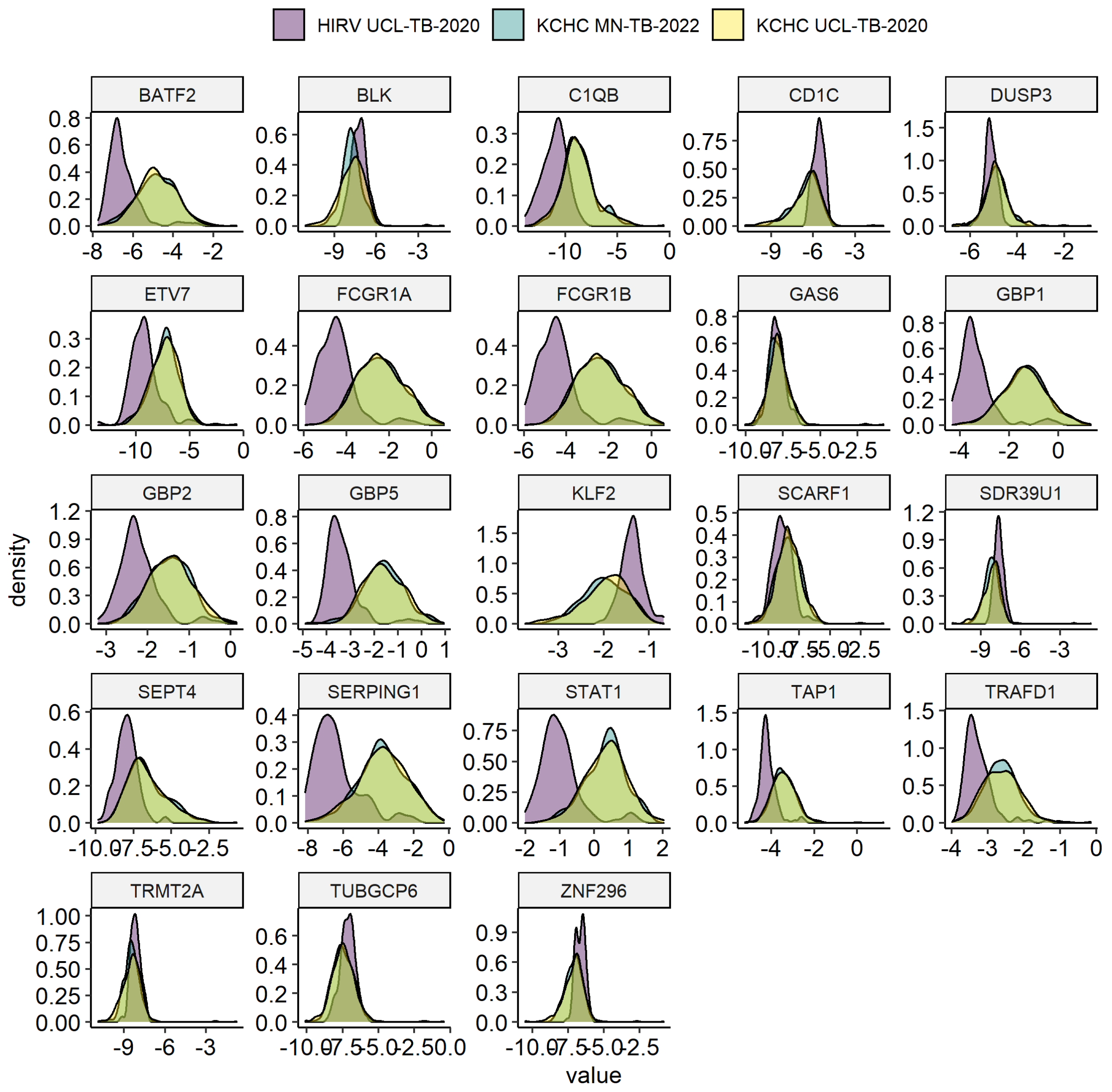

Distribution of gene expression values by Nanostring codeset batch after COMBAT correction.

Density distributions of Nanostring gene expression data normalised to GAPDH following COMBAT batch correction, faceted by target gene, and subset by study group (prefix) and codeset batch (suffix). HIRV = healthy control population of patients with latent TB infection; KCHC= PLHIV study cohort; UCL-TB-2020 and MN-TB-2022 represent different codeset batches.

Supplementary Figure 7

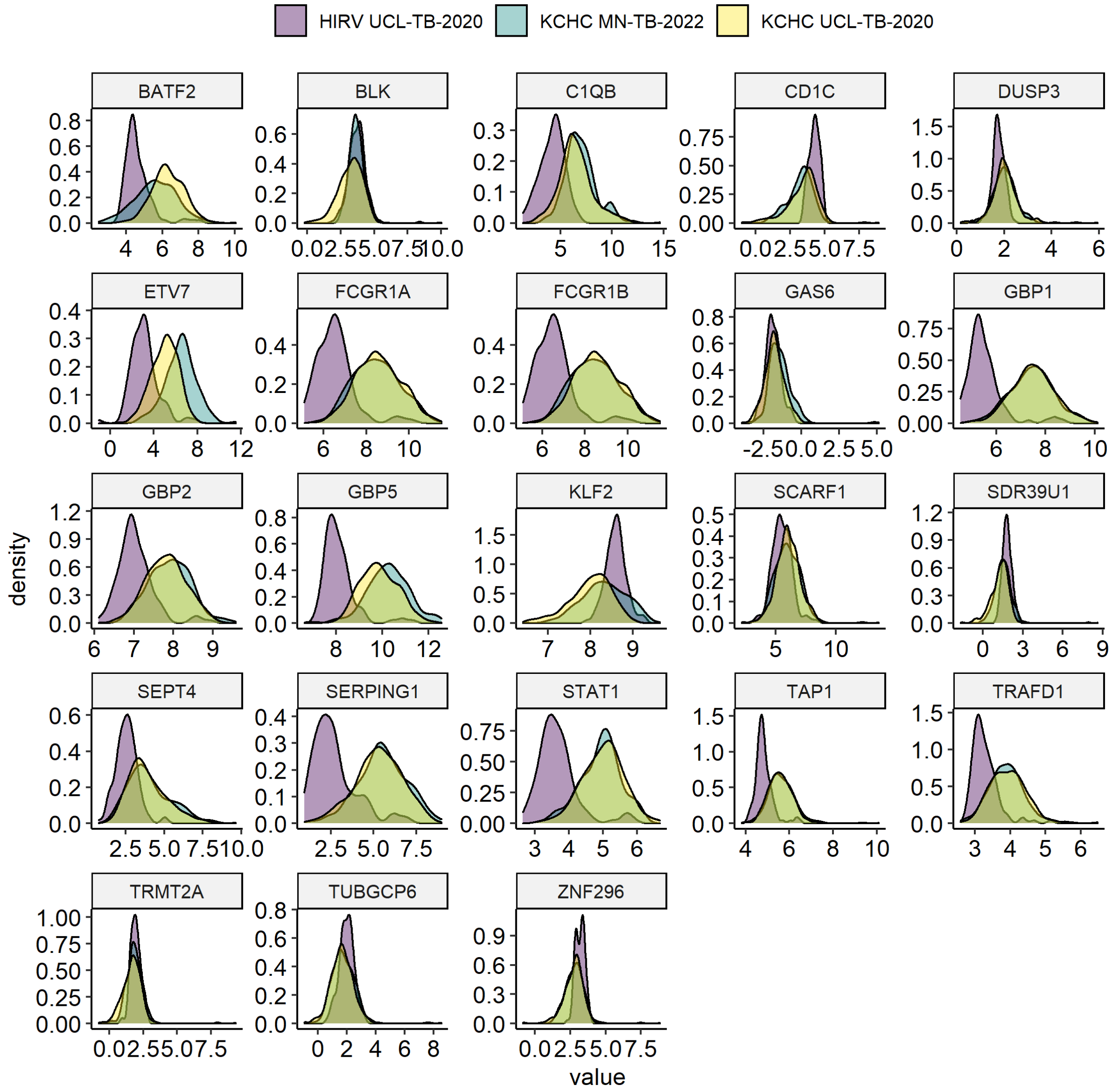

Distribution of gene expression values by Nanostring codeset batch after reference RNA normalisation

Density distributions of Nanostring gene expression data normalised to GAPDH following reference RNA normalisation, faceted by target gene, and subset by study group (prefix) and codeset batch (suffix). HIRV = healthy control population of patients with latent TB infection; KCHC= PLHIV study cohort; UCL-TB-2020 and MN-TB-2022 represent different codeset batches.

Supplementary Figure 8

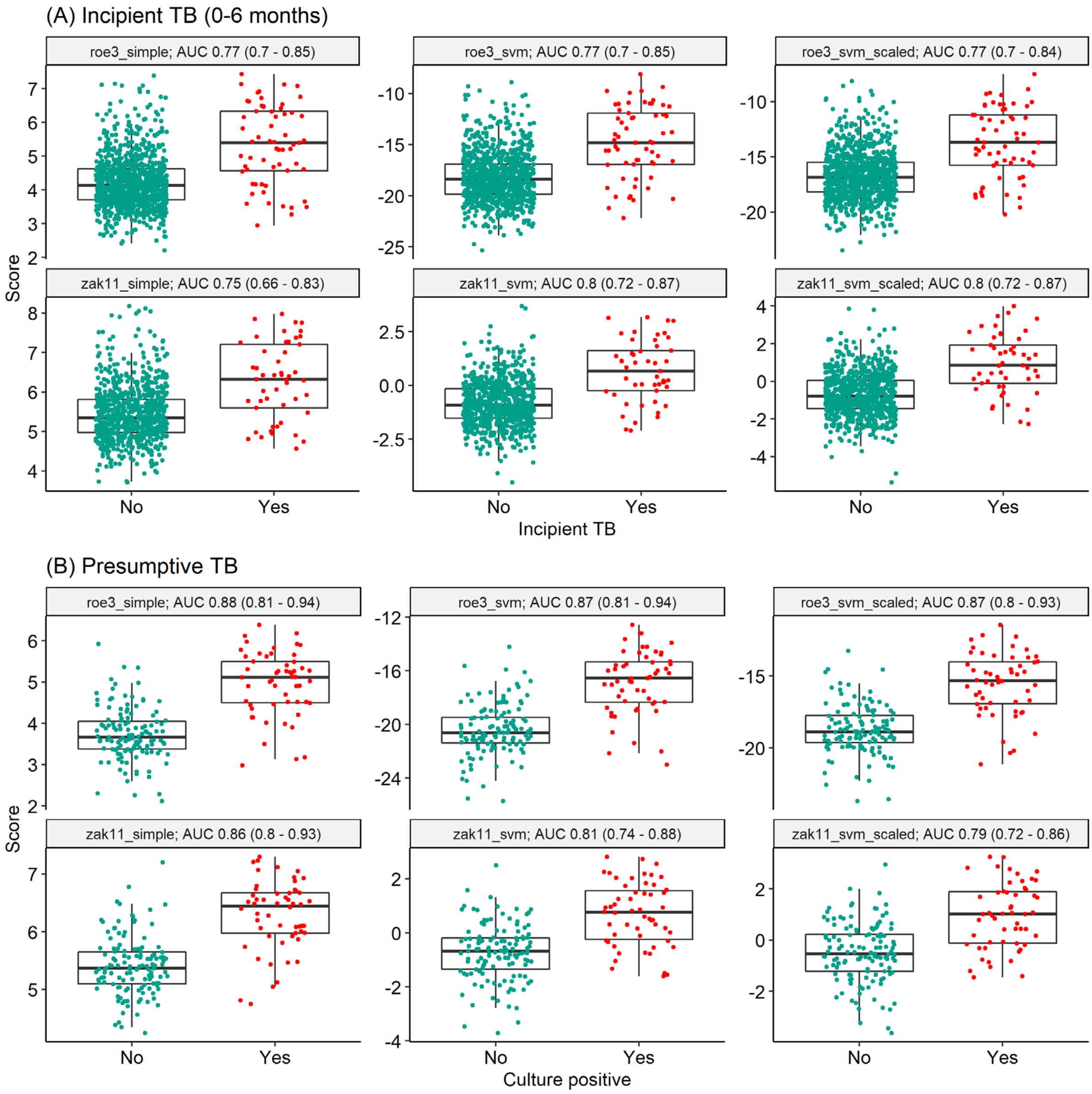

Discrimination of Roe3 and Zak11 signatures TB using simple geometric mean calculations.

Data points show gene blood RNA signature scores for (A) patients with and without incipient TB from a previously reported individual participant data meta-analysis[10] and (B) patients with and without prevalent TB from a cohort of symptomatic individuals presenting for evaluation[9]. Plots are faceted by signature (prefix, Roe 3 or Zak11) and approach to signature score derivation (simple, svm, scaled svm). Plot titles show between-group discrimination as AUROCs with 95% confidence intervals. The “simple” approach reflects a geometric mean calculation. Overall discriminatory performance was similar for both signatures between the geometric mean and support vector machine approaches.

Supplementary Figure 9

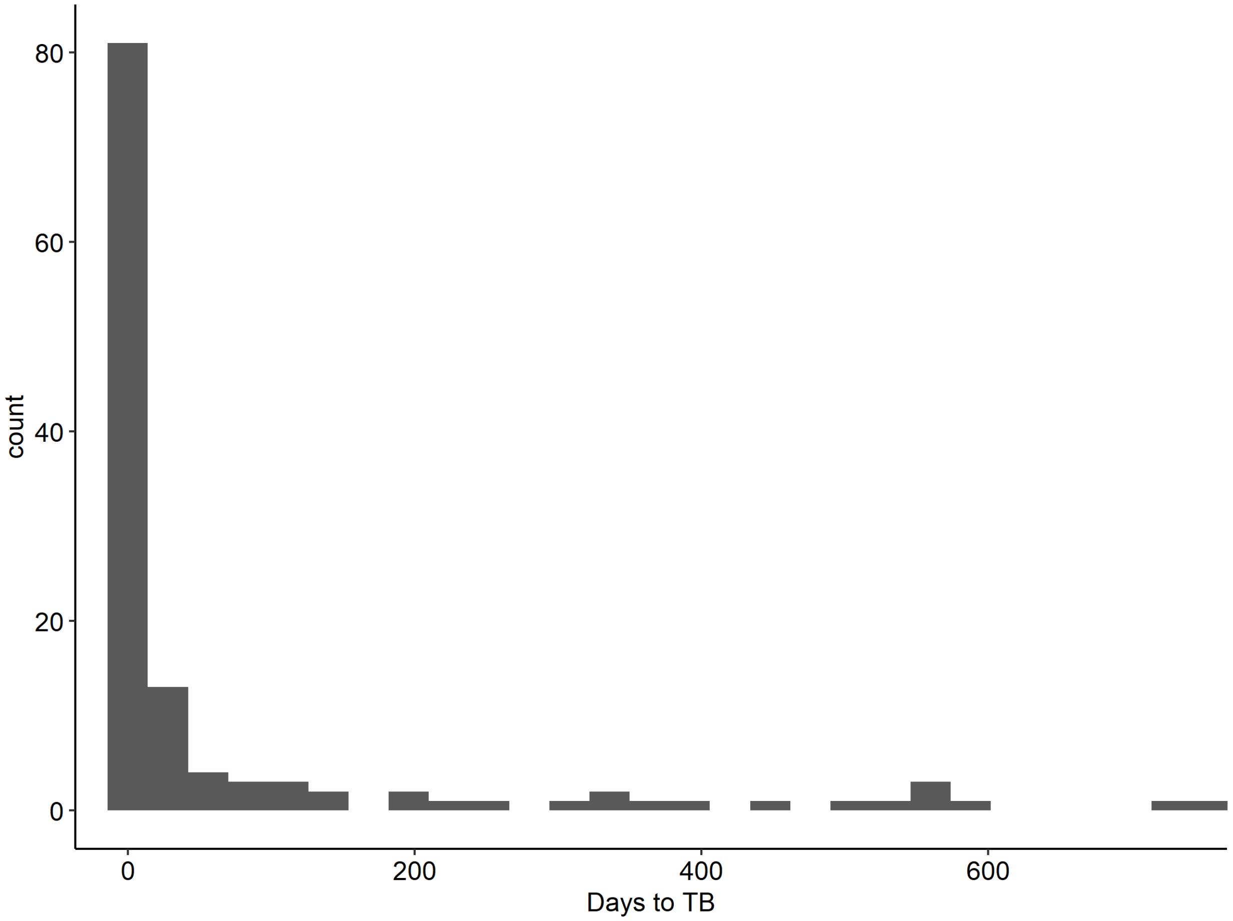

Prevalent in incident TB diagnoses by time from study enrolment.

Frequency distribution of time intervals between study enrolment and first TB diagnosis or treatment initiation recorded in study or registry data in the PLHIV study cohort.

Supplementary Figure 10

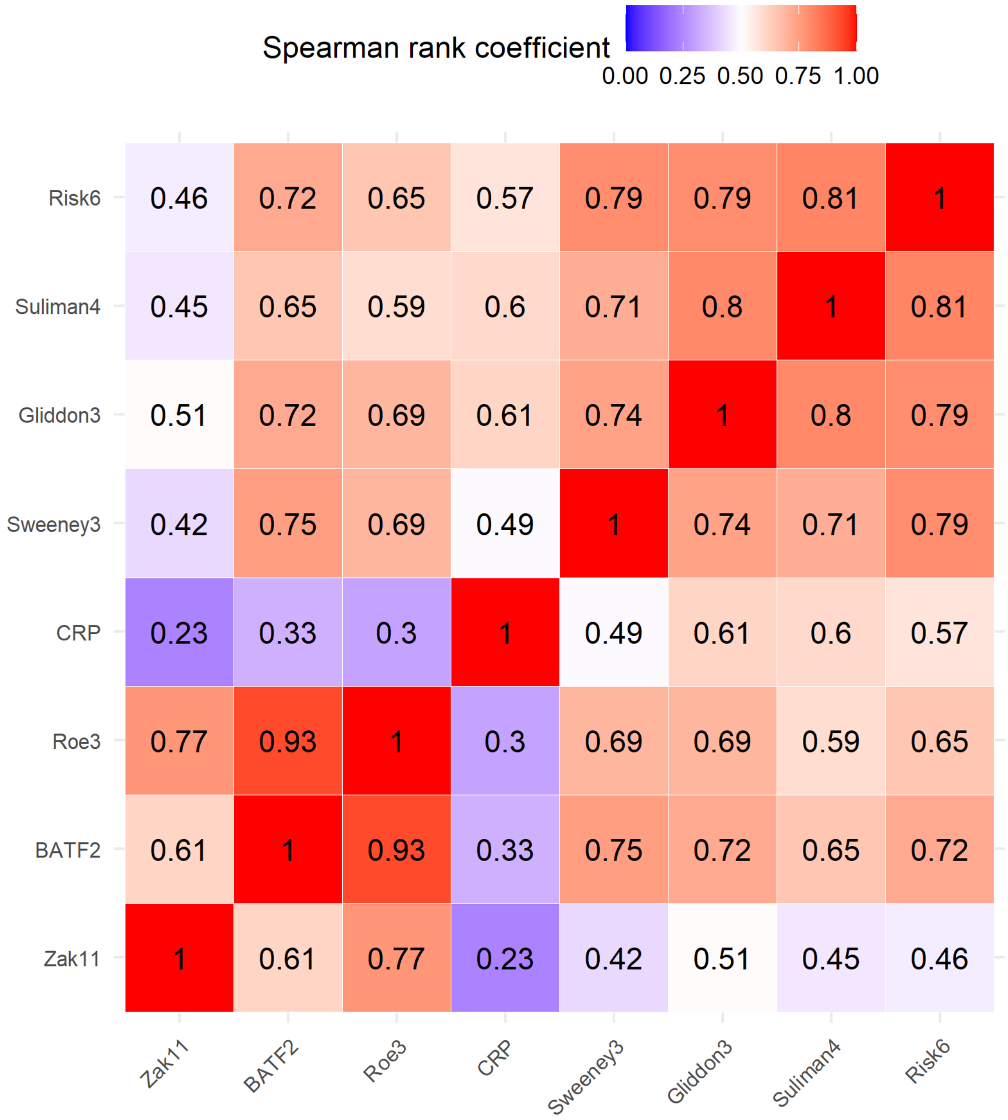

Co-correlation of blood RNA biomarkers and CRP

Spearman rank co-correlation matrix of blood RNA signatures and CRP (n=707), with hierarchical clustering using complete linkage method.

Supplementary Figure 11

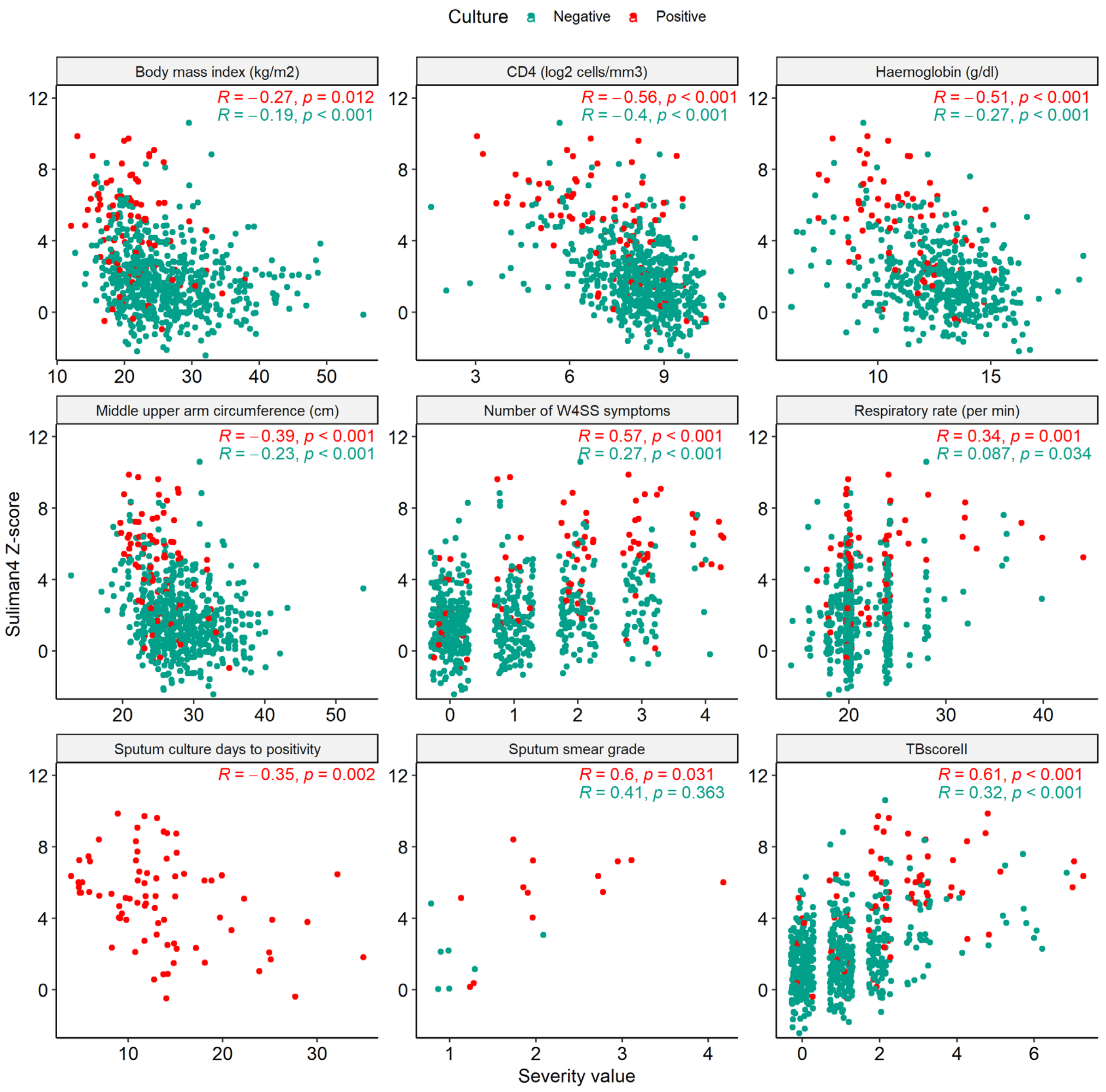

Associations between Suliman4 blood RNA biomarker Z-score sand indices of HIV/TB disease severity

Individual scatter plots of Suliman4 blood RNA signature Z score with selected variables (indicated) associated with HIV and/or TB disease burden/severity, for all participant data (N=676) stratified by sputum Mtb culture result. Spearman correlation coefficients and p values are shown for each pairwise analysis. Sputum smear grade is shown as 1 (scanty), 2 (+), 3 (++) and 4 (+++).

Supplementary Figure 12

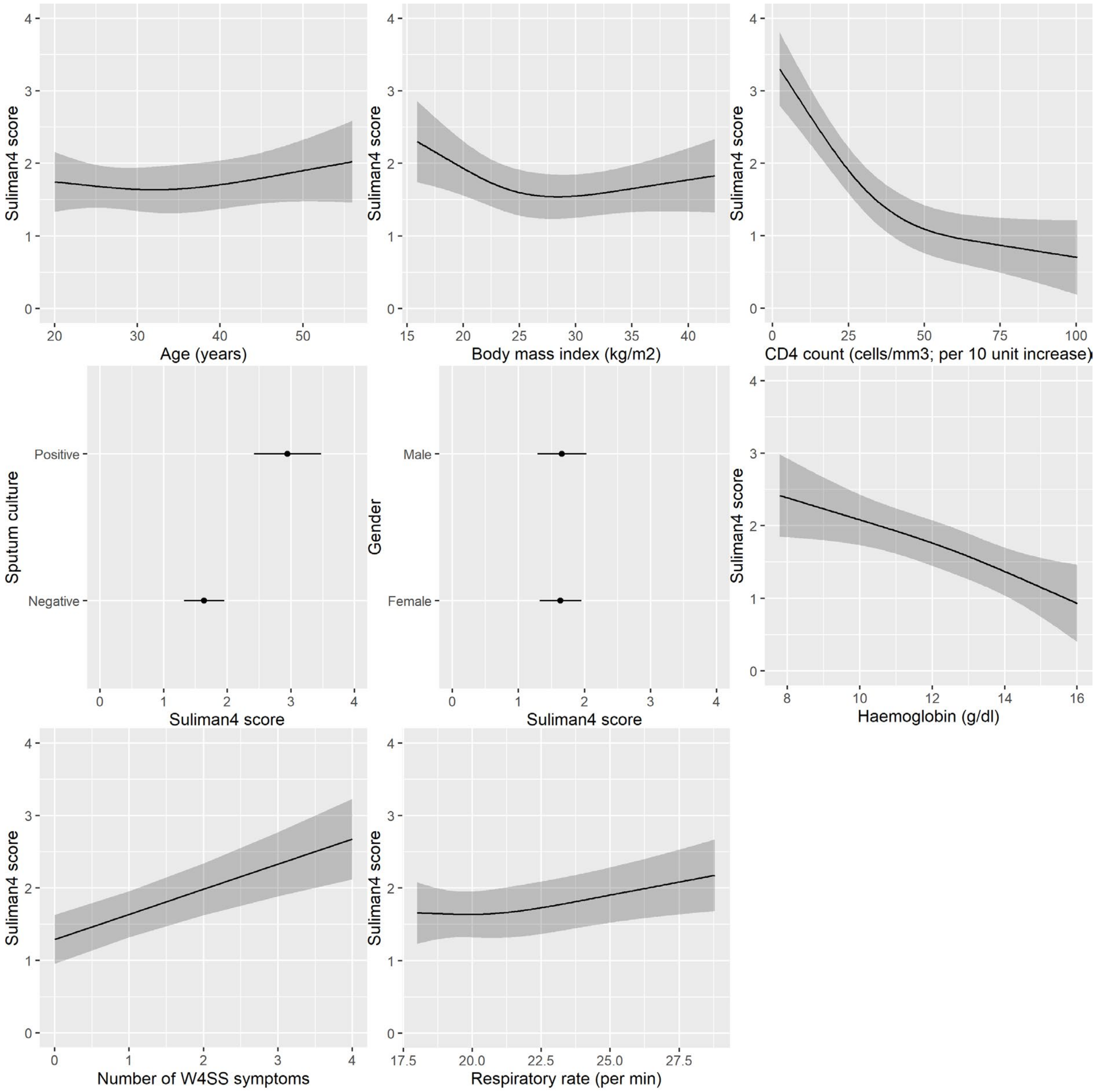

Linear regression multivariable model of predictors for Suliman4 blood RNA signature scores

Plots show associations between each variable and Suliman4 blood RNA signature scores from a multivariable linear regression model including restricted cubic splines to model non-linear associations.

Supplementary Figure 13

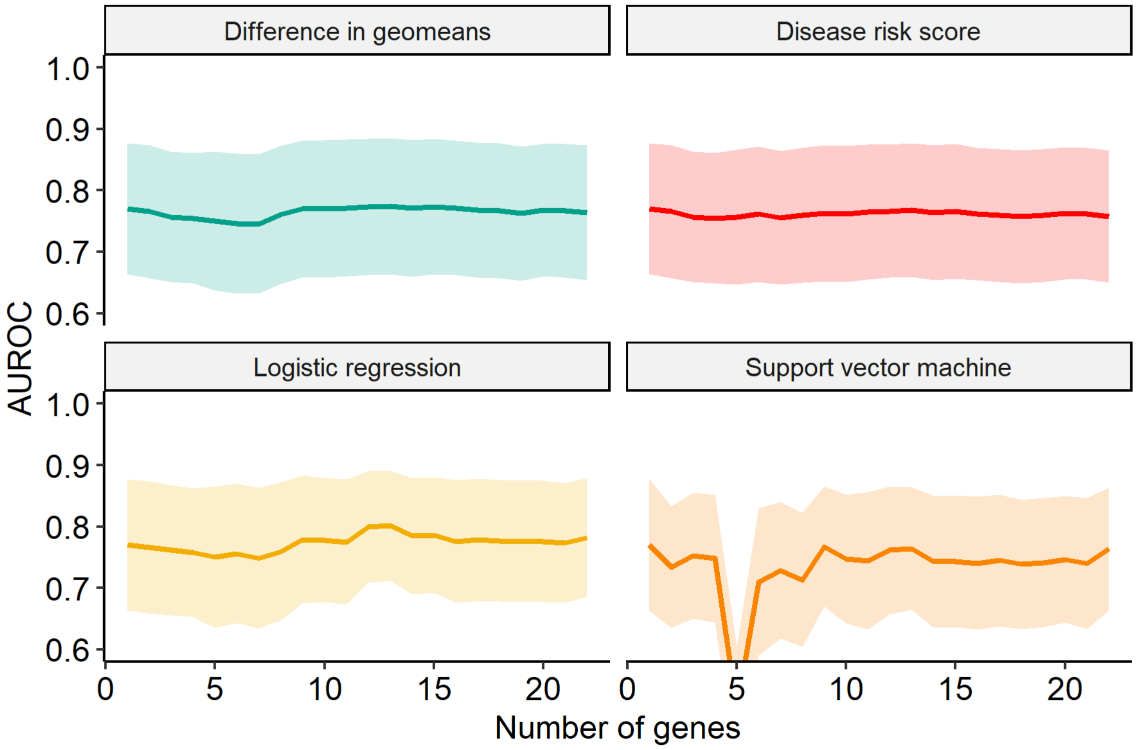

Greedy forward search for optimal signature to discriminate between PLHIV with and without culture positive TB.

Discrimination accuracy (AUROC with 95% confidence intervals) for people with and without culture positive TB in held out validation cases from the PLHIV study cohort using increasing numbers of genes added iteratively in order of their discrimination for culture positive TB as single predictors, and combined into a single signature score by each of the four faceted methods indicated.

Supplementary Figure 14

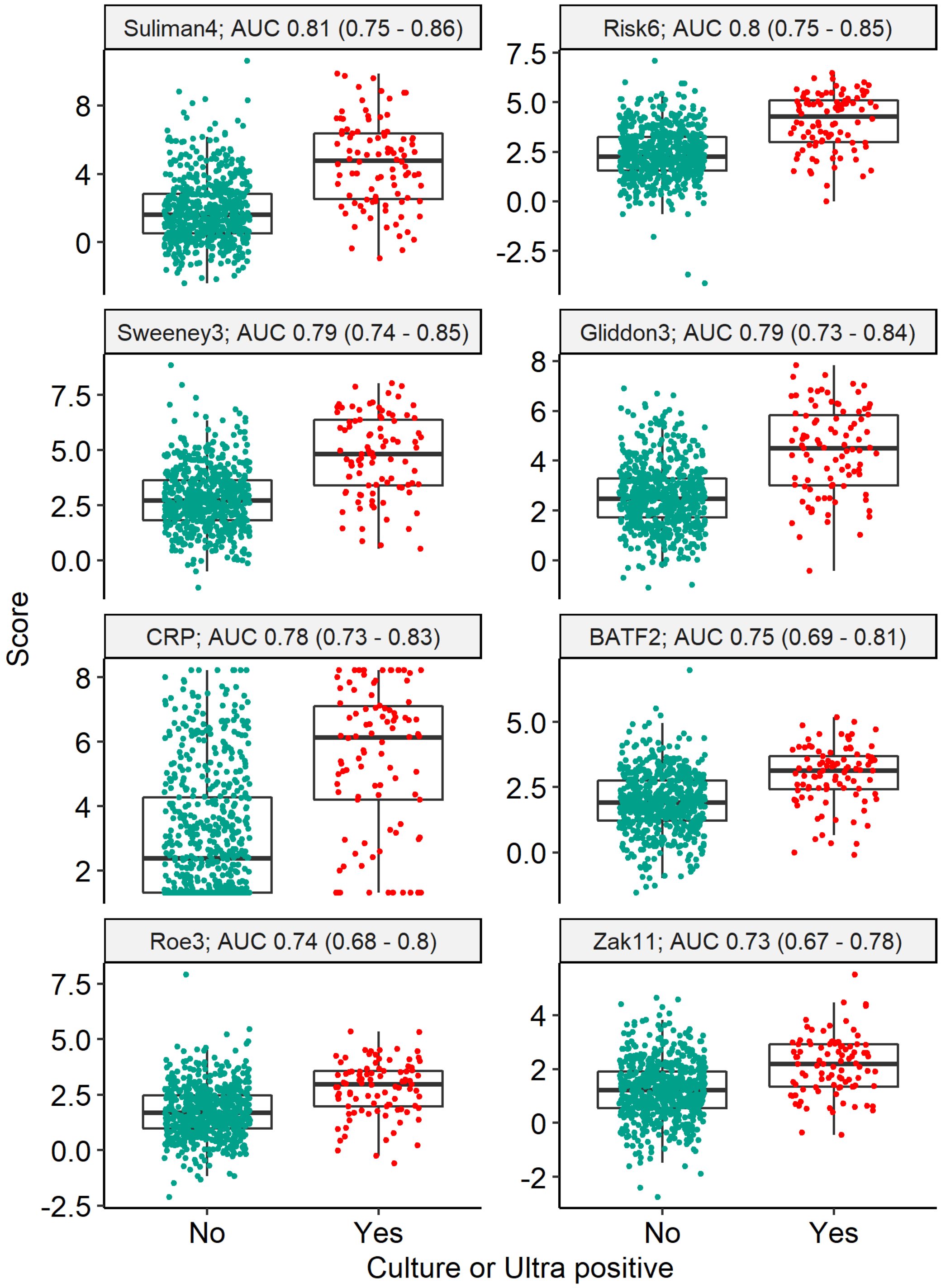

Sensitivity analysis: Sputum culture or Ultra positivity

Blood RNA signature Z scores and log-2 transformed CRP (mg/L) measurements, and discrimination AUROC with 95% confidence intervals for TB sputum culture or Ultra positivity (N = 698 participants).

Supplementary Figure 15

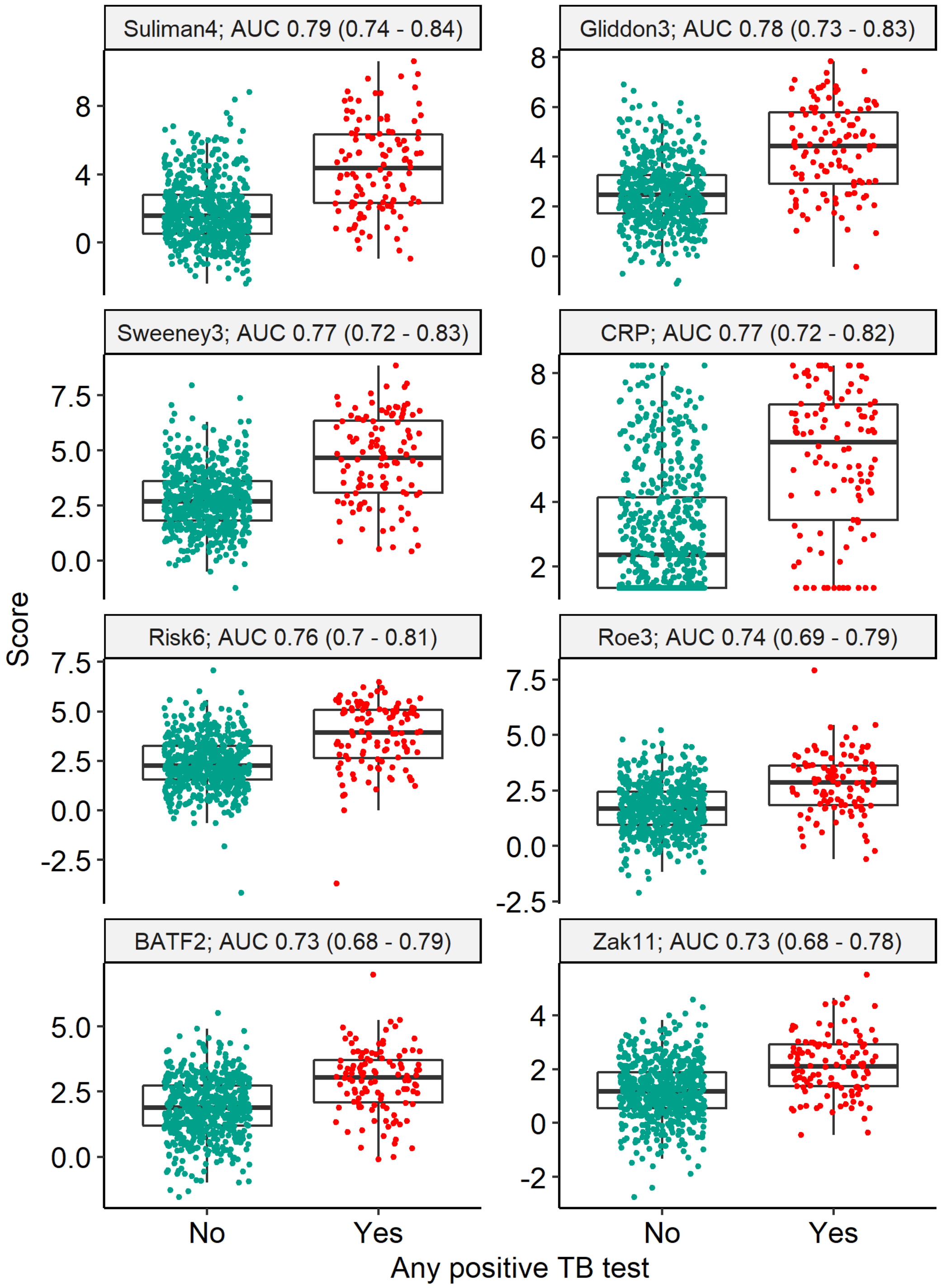

Sensitivity analysis: Any positive TB test

Blood RNA signature Z scores and log-2 transformed CRP (mg/L) measurements, and discrimination AUROC with 95% confidence intervals for any positive TB test, including urine LAM and urine Ultra (N = 699 participants).

Supplementary Figure 16

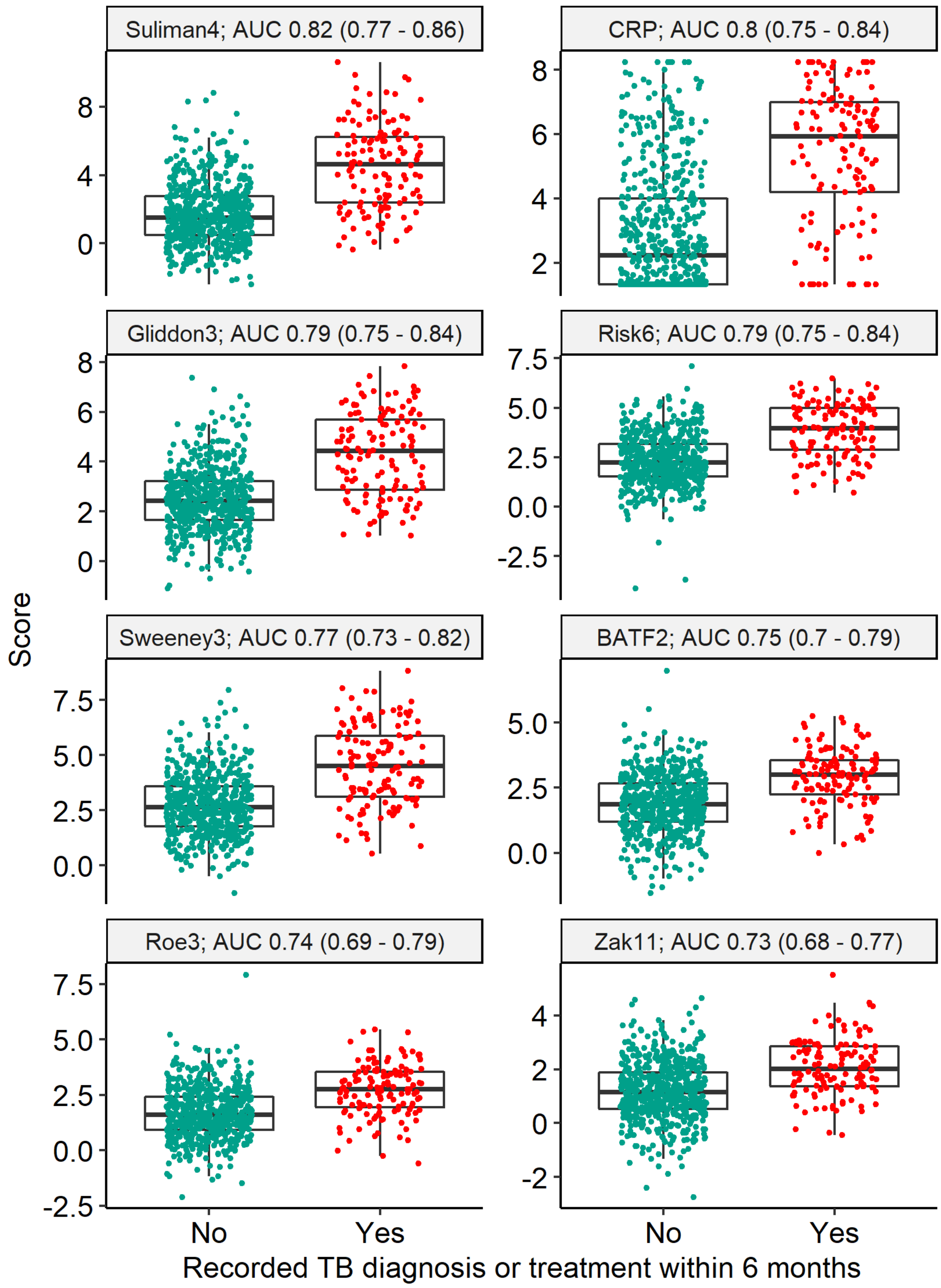

Sensitivity analysis: Recorded TB diagnosis or treatment within 6 months

Blood RNA signature Z scores and log-2 transformed CRP (mg/L) measurements, and discrimination AUROC with 95% confidence intervals for outcome of recorded TB diagnosis or treatment within 6 months (n = 707 participants).

Supplementary Figure 17

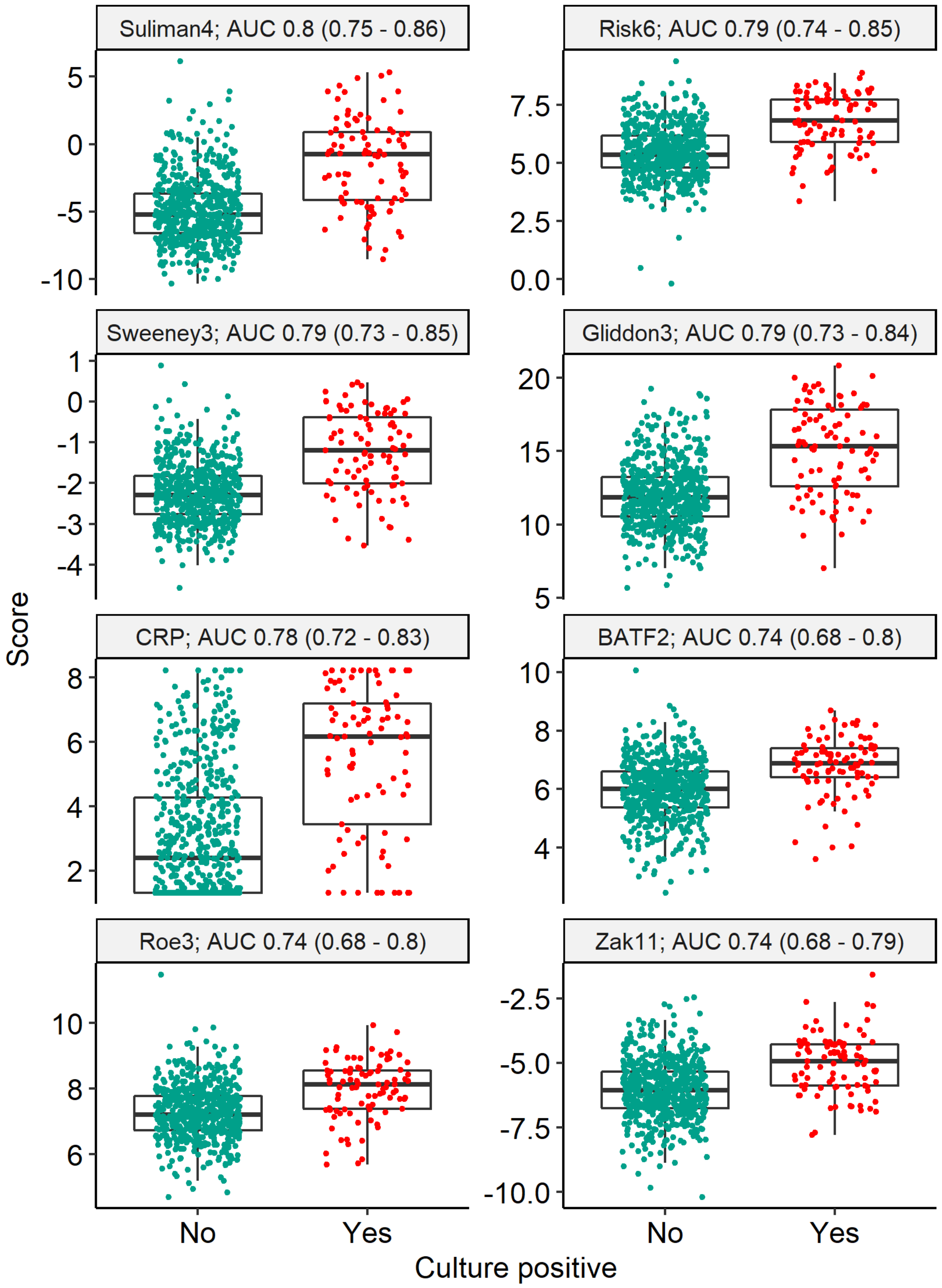

Sensitivity analysis: Using reference RNA-normalised data

Blood RNA signature scores and log-2 transformed CRP (mg/L) measurements, and discrimination AUROC with 95% confidence intervals for sputum culture (n = 676 participants), using reference RNA normalisation of Nanostring data as sensitivity analysis.

References

1 Roe JK, Thomas N, Gil E, *et al.* Blood transcriptomic diagnosis of pulmonary and extrapulmonary tuberculosis. *JCI insight* 2016; **1**: e87238.

2 Gliddon HD, Kaforou M, Alikian M, *et al.* Identification of reduced host transcriptomic signatures for tuberculosis and digital PCR-based validation and quantification. *bioRxiv* 2019; : 583674.

3 Penn-Nicholson A, Mbandi SK, Thompson E, *et al.* RISK6, a 6-gene transcriptomic signature of TB disease risk, diagnosis and treatment response. *Sci Rep* 2020; **10**: 1–21.

4 Roe J, Venturini C, Gupta RK, *et al.* Blood transcriptomic stratification of short-term risk in contacts of tuberculosis. *Clinical infectious diseases : an official publication of the Infectious Diseases Society of America* 2019; published online March 28. DOI:10.1093/cid/ciz252.

5 Suliman S, Thompson E, Sutherland J, *et al.* Four-gene Pan-African Blood Signature Predicts Progression to Tuberculosis. *American journal of respiratory and critical care medicine* 2018; **197**: 1198–208.

6 Sweeney TE, Braviak L, Tato CM, Khatri P. Genome-wide expression for diagnosis of pulmonary tuberculosis: a multicohort analysis. *The Lancet Respiratory Medicine* 2016; **4**: 213–24.

7 Darboe F, Mbandi SK, Thompson EG, *et al.* Diagnostic performance of an optimized transcriptomic signature of risk of tuberculosis in cryopreserved peripheral blood mononuclear cells. *Tuberculosis* 2018; **108**: 124–6.

8 Zak DE, Penn-Nicholson A, Scriba TJ, *et al.* A blood RNA signature for tuberculosis disease risk: a prospective cohort study. *The Lancet* 2016; **387**: 2312–22.

9 Turner CT, Gupta RK, Tsaliki E, *et al.* Blood transcriptional biomarkers for active pulmonary tuberculosis in a high-burden setting: a prospective, observational, diagnostic accuracy study. *Lancet Respir Med* 2020; **8**. DOI:10.1016/S2213-2600(19)30469-2.

10 Gupta RK, Turner CT, Venturini C, *et al.* Concise whole blood transcriptional signatures for incipient tuberculosis: a systematic review and patient-level pooled meta-analysis. *Lancet Respir Med* 2020; **8**. DOI:10.1016/S2213-2600(19)30282-6.
