# Supplementary material for: Blood RNA biomarkers for tuberculosis screening in people living with HIV before antiretroviral therapy initiation: a diagnostic accuracy study
Source: Lancet Glob Health. Author manuscript; Available in PMC 2026 Jul 21. (PMC7619259; doi:10.1016/S2214-109X(24)00029-9)
Supplement: Supplementary appendix 2 [file EMS196790-supplement-Supplementary_appendix_2.pdf]

THE LANCET
Global Health

Supplementary appendix 2

This appendix formed part of the original submission and has been peer reviewed. We post it as supplied by the authors.

Supplement to: Mann T, Gupta RK, Reeve BWP, et al. Blood RNA biomarkers for tuberculosis screening in people living with HIV before antiretroviral therapy initiation: a diagnostic accuracy study. *Lancet Glob Health* 2024; published online April 4. https://doi.org/10.1016/S2214-109X(24)00029-9.

HUGO Gen	Ensemble(	Probe NSID	BATF2	Gliddon3	RISK6	RISK11	Roe3	Suliman4	Sweeney3
BATF2	ENSG0000	NM_13845	1	0	0	1	1	0	0
GBP5	ENSG0000	NM_05294	0	0	0	1	1	0	1
SCARF1	ENSG0000	NM_00369	0	0	0	1	1	0	0
FCGR1A	ENSG0000	NM_00056	0	1	1	1	0	0	0
GBP2	ENSG0000	NM_00412	0	0	1	1	0	0	0
SERPING1	ENSG0000	NM_00006	0	0	1	1	0	0	0
SDR39U1	ENSG0000	NM_00129	0	0	1	0	0	0	0
TRMT2A	ENSG0000	NM_00125	0	0	1	0	0	0	0
TUBGCP6	ENSG0000	NM_02046	0	0	1	0	0	0	0
ETV7	ENSG0000	NM_00120	0	0	0	1	0	0	0
GBP1	ENSG0000	NM_00205	0	0	0	1	0	0	0
STAT1	ENSG0000	NM_13926	0	0	0	1	0	0	0
TAP1	ENSG0000	NM_00059	0	0	0	1	0	0	0
TRAFD1	ENSG0000	NM_00114	0	0	0	1	0	0	0
DUSP3	ENSG0000	NM_00409	0	0	0	0	0	0	1
KLF2	ENSG0000	NM_01627	0	0	0	0	0	0	1
C1QB	ENSG0000	NM_00049	0	1	0	0	0	0	0
ZNF296	ENSG0000	NM_14528	0	1	0	0	0	0	0
	ENSG0000	NM_00197	0	0	0	0	0	0	0
BLK	ENSG0000	NM_00171	0	0	0	0	0	1	0
CD177	ENSG0000	NM_02040	0	0	0	0	0	0	0
CD1C	ENSG0000	NM_00176	0	0	0	0	0	1	0
CTSB	ENSG0000	NM_14778	0	0	0	0	0	0	0
GAPDH	ENSG0000	NM_00125	0	0	0	0	0	0	0
GAS6	ENSG0000	NM_00082	0	0	0	0	0	1	0
GPAA1	ENSG0000	NM_00380	0	0	0	0	0	0	0
HK3	ENSG0000	NM_00211	0	0	0	0	0	0	0
IFI27	ENSG0000	NM_00553	0	0	0	0	0	0	0
	ENSG0000	NM_00682	0	0	0	0	0	0	0
JUP	ENSG0000	NM_00223	0	0	0	0	0	0	0
LAX1	ENSG0000	NM_00113	0	0	0	0	0	0	0
SEPTIN4	ENSG0000	NM_00119	0	0	0	0	0	1	0
TBP	ENSG0000	NM_00117	0	0	0	0	0	0	0
TNIP1	ENSG0000	NM_00125	0	0	0	0	0	0	0

Accession	Position	Target Seq	Total Isofo	Isoforms Hit By Probe
NM_13845	2005-2104	GGAGAGA	3	NM_001300808.2;NM_001300807.2;NM_138456.4
NM_05294	1084-1183	TTCTGCTT	3	NM_001134486.4;NM_001391920.1;NM_052942.5
NM_00369	1831-1930	CCAGCCT	4	NM_145350.3;NR_102409.2;NM_003693.4;NR_028075.3
NM_00056	843-942	GAGGATG	12	NM_001378805.1;NR_166123.1;NM_001378804.1;NM_00137881
NM_00412	781-880	GAGCTTTT	1	NM_004120.5
NM_00006	306-405	GACAGAG	2	NM_001032295.2;NM_000062.3
NM_00129	137-236	GGGCCCG	12	NM_001308075.2;NM_001387326.1;NM_001290293.3;NM_00129
NM_00125	2503-2602	GTGGACT	6	NM_182984.5;NM_001257994.2;NM_022727.6;XM_011530139.4;
NM_02046	321-420	GGGCGTG	4	XR_938347.3;XR_007067982.1;XR_001755343.3;NM_020461.4
NM_00120	577-676	GTATGAGC	9	NM_001207040.2;NM_001207038.2;XM_011514659.2;NM_00120
NM_00205	2111-2210	CCAGATG	1	NM_002053.3
NM_13926	456-555	ACAGTGG	15	NM_001384887.1;NM_001384880.1;NM_001384889.1;NM_0013
NM_00059	2076-2175	GTGGCTG	2	NM_000593.6;NM_001292022.2
NM_00114	1476-1575	GACGAGA	2	NM_006700.3;NM_001143906.2
NM_00409	3431-3530	AATCTTAA	1	NM_004090.4
NM_01627	1016-1115	GGAAGTTT	1	NM_016270.4
NM_00049	820-919	AACTCACT	3	NM_000491.5;NM_001371184.3;NM_001378156.1
NM_14528	527-626	TGAGCTG	1	NM_145288.3
NM_00197	1521-1620	TTCAAAGA	6	XM_011527794.2;NM_001256252.2;NM_001256254.2;NM_0019
NM_00171	991-1090	AGCTTCTT	11	XM_047422081.1;XM_047422083.1;NM_001330465.2;XM_047422
NM_02040	1105-1204	ATTGTTAT	3	XM_017027022.2;XM_017027021.3;NM_020406.4
NM_00176	751-850	AGGCTGT	2	NM_001765.3;XM_005245579.6
NM_14778	1055-1154	ACAAAAAC	13	NM_147783.4;NM_001384727.1;NM_001384726.1;NM_0013847
NM_00125	387-486	GAACGGG	6	NM_001256799.3;NM_001357943.2;NM_002046.7;NM_0012897
NM_00082	1340-1439	ACAGGGA	1	NM_000820.4
NM_00380	633-732	TATTGGGC	1	NM_003801.4
NM_00211	496-595	ACTTTGCT	4	XM_047417134.1;XM_011534540.3;XR_941102.3;NM_002115.3
NM_00553	391-490	TCACTGG	11	NM_001366994.1;XM_047431349.1;NM_001130080.3;NM_00128
NM_00682	941-1040	ATCTCTGC	7	NM_006820.4;NM_001375647.1;NM_001375646.1;XM_0067103
NM_00223	1076-1175	CTCGTGC	19	XM_047435941.1;XM_047435942.1;XM_006721874.4;NM_001352
NM_00113	316-415	GAATTGGA	4	NM_001282878.1;NM_001136190.2;NM_017773.4;XM_0067113
NM_00119	1031-1130	TTGGAATC	23	XM_047436309.1;XM_006721952.4;NM_080416.4;NM_00136877
NM_00117	588-687	ACAGTGA	2	NM_001172085.2;NM_003194.5
NM_00125	395-494	AGCCAGC	27	NM_006058.5;XM_006714752.4;XM_047416627.1;NM_0012523

).1;NM_001378809.1;NM_000566.4;NM_001378808.1;NR_166122.1;NR_166121.1;NM_001378807.1

4888.1;NM_001384885.1;NM_001384884.1;XM_006712718.2;NM_139266.3;NM_007315.4;NM_001384881.1;N

082.1;NM_001715.3;XM_011543827.2;XM_047422084.1;XM_011543824.2;XM_011543825.4;XM_011543828.4

8.1;NM_147780.4;NM_147781.4;NM_001384725.1;NM_001384724.1;NM_147782.4;NM_001317237.2;NM_001

3956.2;XM_047431346.1;NM_001366993.1;NM_001288959.2;XM_047431348.1;XM_047431347.1;NM_00128[illegible]

777.2;XM_047435940.1;NM_001352775.2;NM_001352774.2;XM_006721875.2;XM_017024590.2;XM_0474359[illegible]

2.2;XM_024450808.2;NM_080415.4;XM_006721950.5;NM_001256822.2;NM_001368771.2;NM_001256782.2;X[illegible]

5.2;NM_001252385.2;NM_001252392.2;XM_047416620.1;NM_001364487.2;NM_001252390.2;XM_047416624.

38.1;XM_047435939.1;XM_011524758.2;NM_002230.4;XM_047435935.1;NM_001352773.2;XM_047435934.1;X

M_006721955.4;NR_037155.3;NR_104197.2;NM_001198713.2;NM_001363803.2;NR_104196.2;XM_00672194[illegible]

1;NM_001252391.2;NM_001364486.2;XM_047416617.1;XM_047416618.1;NM_001258454.2;NM_001258456.1

.;XM_047416626.1;XM_047416616.1;XM_005268355.3;NM_001258455.1;XM_047416615.1;XM_047416621.1;X

[illegible]M_047416625.1;XM_047416623.1;XM_047416619.1;XM_047416622.1;NM_001252393.2
