# Supplementary material for: Blood RNA biomarkers for tuberculosis screening in people living with HIV before antiretroviral therapy initiation: a diagnostic accuracy study
Source: Lancet Glob Health. Author manuscript; Available in PMC 2026 Jul 21. (PMC7619259; doi:10.1016/S2214-109X(24)00029-9)
Supplement: Supplementary appendix 3 [file EMS196790-supplement-Supplementary_appendix_3.pdf]

THE LANCET
Global Health

Supplementary appendix 3

This appendix formed part of the original submission and has been peer reviewed. We post it as supplied by the authors.

Supplement to: Mann T, Gupta RK, Reeve BWP, et al. Blood RNA biomarkers for tuberculosis screening in people living with HIV before antiretroviral therapy initiation: a diagnostic accuracy study. *Lancet Glob Health* 2024; published online April 4. https://doi.org/10.1016/S2214-109X(24)00029-9.

UIN	Age (years)	Gender	CD4 (cells/	WHO 4-syr	CRP (mg/L	BATF2	GBP5	DUSP3	KLF2
ARTTB046	39	Female	844	1	2.5	0.843927	-1.98848	-5.25468	-1.37928
ARTTB047	28	Female	563	0	2.5	2.45591	-1.25456	-5.14233	-1.67845
ARTTB048	21	Female	371	0	5.61	1.106038	-2.21274	-4.89721	-1.71972
ARTTB049	38	Female	614	1	2.5	2.088955	-1.4992	-5.30574	-1.22453
ARTTB050	18	Female	653	1	2.5	1.957899	-2.01906	-5.11169	-1.60624
ARTTB051	39	Female	1234	0	8.41	-0.99085	-3.4971	-5.02999	-1.31738
ARTTB052	33	Male	240	1	53.28	2.600071	-1.31572	-5.06063	-1.55465
ARTTB054	23	Female	385	0	2.5	1.410273	-1.97265	-5.31139	-1.22548
ARTTB056	36	Female	257	0	7.55	2.075849	-1.24437	-4.72359	-1.51339
ARTTB057	34	Male	107	0	3.9	1.721999	-1.89674	-4.83593	-1.54434
ARTTB059	44	Female	223	0	2.5	1.472994	-2.01906	-5.02999	-1.34833
ARTTB060	31	Male	365	0	4.45	1.237094	-2.09042	-5.40787	-1.97763
ARTTB061	42	Male	199	0	2.5	1.577838	-2.29428	-5.9696	-1.96731
ARTTB062	24	Male	326	0	2.5	0.660449	-2.51854	-5.82662	-1.73003
ARTTB063	34	Male	402	0	2.5	2.416593	-2.05984	-5.44873	-1.61655
ARTTB064	26	Female	536	0	2.5	2.835971	-1.16282	-5.05041	-1.32769
ARTTB065	35	Female	169	0	81.67	0.241072	-2.5695	-4.90743	-2.81326
ARTTB066	22	Female	710	0	2.5	2.259327	-1.39727	-4.60103	-1.90541
ARTTB067	35	Female	629	0	6.92	2.875288	-0.80606	-4.56018	-2.18395
ARTTB068	24	Female	583	0	2.64	1.486099	-1.84578	-5.00956	-1.82288
ARTTB069	27	Female	389	0	2.5	2.062743	-2.09042	-5.26489	-1.43086
ARTTB070	30	Female	106	0	53.35	2.547649	-1.55017	-4.85636	-2.13237
ARTTB072	34	Male	306	0	7.89	3.150504	-0.70412	-4.91764	-1.74035
ARTTB073	49	Female	217	1	9.03	4.015471	0.152117	-3.84525	-2.37997
ARTTB075	42	Female	198	1	3.16	1.420571	-2.81414	-4.887	-1.6475
ARTTB076	26	Female	443	1	2.5	0.948772	-2.7224	-4.94828	-1.33801
ARTTB077	52	Male	162	1	85.02	3.019449	-0.49006	-4.44783	-2.18395
ARTTB078	32	Female	193	0	63.16	1.237094	-2.80395	-5.08105	-2.20459
ARTTB079	43	Male	510	0	5.77	3.255349	-0.80606	-4.66231	-1.38959
ARTTB080	37	Male	395	0	2.5	3.320877	-1.0405	-4.6521	-1.78162
ARTTB081	44	Female	433	0	2.5	1.474633	-1.40975	-4.95075	-2.16805
ARTTB082	29	Male	583	1	2.66	1.813738	-1.56036	-5.28532	-1.66813
ARTTB083	35	Male	437	0	2.5	1.270825	-1.69597	-4.55216	-1.13307
ARTTB086	25	Male	296	0	15.89	0.319705	-2.97724	-4.86657	-2.41092
ARTTB088	27	Male	299	0	2.5	1.001194	-2.75298	-5.07084	-1.26579
ARTTB089	42	Female	328	0	7.14	2.298643	-1.29534	-4.95849	-1.49276
ARTTB090	33	Female	443	0	4.55	2.534543	-0.80606	-4.82572	-1.38959
ARTTB091	26	Female	264	0	2.5	2.626282	-1.08128	-5.14233	-1.71972
ARTTB092	24	Male	351	0	2.5	0.359051	-2.66912	-5.43477	-1.91854
ARTTB093	37	Male	438	0	2.5	0.463866	-2.67144	-4.95849	-1.45149
ARTTB094	46	Female	655	0	3.1	0.267283	-2.34525	-5.41809	-1.53402
ARTTB096	37	Female	404	1	2.5	0.348324	-2.81223	-5.52018	-2.0664
ARTTB097	30	Female	105	1	20.59	2.600071	-1.94771	-4.86657	-2.08079
ARTTB098	42	Male	305	0	13.01	2.965652	-0.56063	-4.704	-2.29742
ARTTB099	37	Male	326	0	5.32	0.830822	-2.64086	-5.92875	-1.56497
ARTTB100	27	Female	585	0	2.5	-0.45618	-2.15393	-4.91279	-1.49346
ARTTB101	32	Male	43	1	73	2.10206	-2.71221	-5.00956	-3.49414

ARTTB102	25	Female	200	1	3.99	2.442805	-1.87636	-5.14233	-1.85383
ARTTB103	24	Female	201	0	79.17	3.779571	-0.276	-4.50911	-2.08079
ARTTB104	28	Female	566	0	8.69	2.351066	-1.09147	-5.00956	-1.62687
ARTTB105	29	Female	354	0	2.5	0.831028	-1.84862	-5.25445	-1.52119
ARTTB106	27	Male	409	0	2.53	0.909455	-2.4268	-4.46826	-1.84351
ARTTB107	24	Female	253	0	2.9	2.547649	-1.5094	-4.87679	-1.73003
ARTTB108	38	Female	59	1	195.95	3.229138	-1.24437	-4.27421	-3.44256
ARTTB109	23	Female	874	1	33.48	1.787527	-2.17196	-4.86657	-1.86415
ARTTB110	44	Male	236	1	20.42	5.181865	0.926811	-3.55928	-2.63788
ARTTB111	29	Male	452	0	2.5	2.324855	-1.31572	-5.1219	-2.15301
ARTTB112	38	Female	99	0	9.48	2.141377	-1.51959	-4.70316	-1.16263
ARTTB113	23	Female	280	0	4.7	3.425721	-0.55122	-4.90743	-2.19427
ARTTB114	20	Male	96	1	72	4.094104	-0.10272	-3.58992	-2.51408
ARTTB115	24	Female	101	0	12.74	1.433677	-1.68268	-4.94828	-1.53402
ARTTB116	35	Female	671	0	2.5	0.896349	-2.58989	-5.44873	-1.22453
ARTTB117	26	Male	233	0	2.5	1.171566	-2.14138	-5.24446	-1.0182
ARTTB119	42	Male	364	1	3.75	1.39436	-1.60114	-5.01977	-1.55465
ARTTB120	37	Female	410	0	2.5	1.538521	-2.04964	-4.83593	-1.7094
ARTTB121	34	Female	100	0	2.5	2.272432	-1.77442	-5.28532	-1.39991
ARTTB122	49	Male	390	1	2.5	2.180693	-1.40746	-4.75423	-1.34833
ARTTB123	25	Male	537	1	2.5	1.368149	-2.53892	-5.25468	-1.65782
ARTTB124	50	Male	241	0	2.88	2.835971	-0.92838	-4.90743	-1.09042
ARTTB125	33	Male	173	0	20.95	0.463866	-2.90588	-5.59171	-1.67845
ARTTB126	51	Female	1311	0	5.18	0.791505	-2.38602	-5.22404	-1.65782
ARTTB127	26	Female	594	0	2.5	1.656471	-1.71326	-5.07084	-1.39991
ARTTB128	56	Female	411	1	2.5	1.590944	-1.9681	-5.00956	-2.16332
ARTTB129	30	Male	25	0	19.78	1.997216	-2.40641	-4.887	-1.79193
ARTTB131	22	Male	206	1	3.23	0.241072	-2.38602	-5.75512	-1.25548
ARTTB132	29	Female	958	0	2.5	1.721999	-1.70307	-4.64188	-1.17295
ARTTB133	27	Female	772	0	2.76	1.132249	-2.55931	-4.7338	-1.68877
ARTTB134	50	Female	136	1	19.88	2.049638	-1.89674	-5.02999	-1.93636
ARTTB135	42	Female	856	0	17.26	1.708894	-1.89674	-4.95849	-1.76098
ARTTB136	32	Female	127	1	7.22	1.132249	-2.2637	-5.44873	-1.28643
ARTTB137	28	Female	542	1	2.5	2.141377	-1.46862	-5.13212	-1.68877
ARTTB138	28	Male	297	0	10.09	2.652493	-1.21379	-5.1219	-1.6475
ARTTB139	47	Female	407	0	69.26	1.328833	-2.01906	-5.22404	-1.47212
ARTTB140	34	Male	317	1	142.34	0.555605	-2.48796	-4.69295	-2.53471
ARTTB142	32	Female	19	0	2.5	0.857033	-3.06898	-6.54154	-1.94668
ARTTB143	27	Female	171	1	10.22	1.721999	-1.5094	-5.41809	-1.29674
ARTTB144	29	Female	340	0	16.76	0.961877	-2.62047	-5.36702	-2.60693
ARTTB145	43	Male	229	0	11.72	0.843927	-2.29428	-5.06063	-1.48244
ARTTB146	38	Female	410	1	2.5	2.062743	-1.17302	-4.77466	-1.10073
ARTTB148	24	Female	1413	0	7.28	-0.65011	-3.60922	-4.98913	-1.65782
ARTTB151	36	Male	570	0	2.5	-0.57418	-3.06983	-5.56763	-1.32713
ARTTB152	22	Male	297	1	2.5	1.345912	-2.29704	-5.41579	-1.90006
ARTTB153	45	Female	232	1	7.59	2.654576	-1.23802	-4.91279	-1.48422
ARTTB155	39	Male	498	1	106.94	3.652164	-0.35073	-4.3149	-2.15881
ARTTB156	23	Male	108	0	5.81	3.910626	0.243857	-4.62146	-2.02921

ARTTB157	29	Female	471	1	177.89	3.189821	-0.60219	-4.37634	-2.58629
ARTTB158	23	Female	351	0	6.27	1.813738	-1.4992	-4.69295	-1.74035
ARTTB159	32	Male	211	0	2.5	1.813738	-2.50834	-4.97892	-1.47212
ARTTB160	21	Female	729	0	2.5	0.219603	-2.57372	-5.52967	-1.16079
ARTTB161	39	Male	112	0	4.38	2.442805	-1.64191	-4.87679	-1.97763
ARTTB162	44	Male	262	1	93.67	4.054788	-0.06194	-3.90653	-2.82357
ARTTB163	26	Female	8	1	37.63	4.526587	-0.1333	-3.20182	-1.95699
ARTTB165	52	Female	413	0	6.68	1.903703	-0.9995	-5.31139	-1.40106
ARTTB166	19	Male	327	1	71.4	2.933471	-0.58925	-4.52369	-1.98323
ARTTB167	25	Female	611	0	2.5	1.120651	-2.32566	-5.16903	-1.69676
ARTTB169	30	Female	1193	0	2.91	0.487772	-2.24933	-5.11209	-1.44726
ARTTB170	46	Female	337	1	19.01	4.177775	-0.76098	-4.54267	-1.207
ARTTB171	35	Male	333	0	2.5	2.128271	-1.32592	-5.51001	-1.19358
ARTTB172	35	Female	538	0	9.2	4.631432	-0.7347	-4.32527	-1.06978
ARTTB174	32	Male	447	0	11.27	0.863209	-2.2875	-4.91279	-1.53043
ARTTB175	26	Female	735	0	2.5	2.182599	-0.87547	-4.66604	-1.59511
ARTTB178	28	Male	134	0	32.5	4.038327	0.383899	-3.80241	-1.92779
ARTTB179	21	Female	538	1	2.5	0.251784	-2.72637	-4.83687	-1.37333
ARTTB180	39	Female	694	0	2.5	0.830822	-2.74279	-6.54154	-1.90541
ARTTB181	32	Female	57	1	80.32	4.945965	0.784104	-3.47758	-2.04984
ARTTB182	48	Male	216	0	4.53	3.111188	-0.54103	-4.62146	-1.18326
ARTTB183	45	Female	236	0	73.66	3.491263	-0.30303	-4.23897	-2.1126
ARTTB184	29	Male	73	0	5.24	3.229138	-0.82644	-4.39677	-2.53471
ARTTB185	24	Female	428	0	2.5	1.94661	-1.03766	-4.81789	-1.26244
ARTTB187	19	Female	207	0	4.57	4.067893	-0.40852	-4.74402	-1.53402
ARTTB188	44	Male	486	0	2.5	1.710622	-1.39067	-4.84636	-1.80765
ARTTB189	52	Female	245	0	17.93	4.198949	-1.48901	-4.1925	-2.04984
ARTTB190	37	Male	481	0	2.5	1.892371	-1.56036	-5.00956	-1.54434
ARTTB191	24	Female	681	0	2.5	0.817716	-2.16177	-5.33638	-0.88409
ARTTB192	21	Female	913	0	16.09	0.319705	-2.60008	-4.7338	-1.43086
ARTTB194	25	Female	22	0	60.65	3.609199	-1.39727	-4.12101	-1.60624
ARTTB195	31	Male	63	1	300	3.412615	-0.31678	-4.53975	-1.80225
ARTTB196	37	Male	194	1	33.26	2.77257	-1.06629	-4.4098	-1.65056
ARTTB197	39	Male	205	1	3.52	-0.05929	-2.0299	-6.00419	-2.02019
ARTTB199	29	Male	184	0	35.12	4.306496	0.326654	-3.97324	-1.76145
ARTTB200	25	Female	101	0	2.5	2.257686	-1.01858	-4.8084	-1.05914
ARTTB202	34	Male	265	1	31.98	2.890564	-0.56063	-4.5047	-1.49346
ARTTB203	23	Female	696	0	2.5	-0.09147	-2.18255	-5.07413	-1.10535
ARTTB205	32	Male	261	0	2.79	0.541406	-2.57372	-4.93177	-1.33637
ARTTB206	57	Male	367	1	85.7	3.019285	-0.75144	-4.63757	-1.40106
ARTTB209	39	Male	163	1	37.43	2.023427	-1.32592	-5.00956	-1.43086
ARTTB212	29	Male	486	0	2.5	2.324855	-1.08128	-5.23425	-1.21421
ARTTB213	33	Female	576	0	4.42	2.141377	-1.71326	-4.70316	-1.50307
ARTTB214	22	Male	453	1	37.22	2.088955	-1.74384	-4.80529	-1.13168
ARTTB215	21	Female	1004	0	15.35	2.233116	-1.89674	-4.86657	-1.30706
ARTTB216	26	Female	401	0	6.36	4.094104	0.090957	-4.18229	-1.27611
ARTTB217	53	Male	260	0	10.21	2.731127	-1.72346	-5.62235	-1.47212
ARTTB218	48	Female	1474	0	2.5	1.590944	-1.37688	-4.63167	-1.54434

ARTTB219	32	Male	62	0	3.64	2.757338	-1.23418	-4.86657	-2.22522
ARTTB220	26	Male	253	0	9.03	1.853055	-2.51854	-5.56107	-1.17295
ARTTB221	37	Male	209	0	69.44	3.687832	-0.34736	-4.41719	-1.82288
ARTTB223	33	Male	8	1	75.56	3.137399	-1.29534	-5.09126	-3.29813
ARTTB224	29	Female	406	0	2.5	2.450768	-0.79915	-4.98872	-1.23472
ARTTB225	25	Female	331	0	2.5	0.935666	-2.77337	-5.0402	-1.60624
ARTTB226	32	Male	235	0	5.69	1.826844	-1.52978	-4.70316	-1.54434
ARTTB227	40	Female	205	0	2.5	2.796654	-1.08128	-5.13212	-1.22453
ARTTB228	30	Female	772	1	141.38	-0.24383	-2.96704	-4.14144	-2.28712
KTB0001	23	Female	453	0	3.62	3.687832	-0.4391	-4.1925	-1.80225
KTB0002	36	Male	297	0	2.5	2.835971	-1.00992	-4.85636	-2.5244
KTB0004	31	Female	415	1	12.59	3.661621	-0.57161	-4.37634	-2.56566
KTB0006	31	Male	50	1	241.52	0.791505	-2.28409	-5.02999	-1.48244
KTB0007	25	Male	96	0	7.21	2.504401	-0.9041	-4.79891	-1.66904
KTB0008	31	Male	266	0	4.28	1.787527	-1.89674	-5.29553	-2.31807
KTB0009	41	Male	313	0	13.9	0.92256	-1.72346	-5.35681	-1.88478
KTB0010	33	Male	685	0	11.48	1.997216	-1.26476	-4.91764	-1.79193
KTB0011	36	Female	349	0	2.5	1.74821	-0.66335	-4.67252	-2.22522
KTB0012	25	Female	384	0	110.77	3.28156	-0.79586	-4.63167	-2.31807
KTB0013	42	Male	699	1	4.8	1.079827	-1.25456	-5.08105	-2.53471
KTB0014	28	Female	490	1	2.5	3.229138	-0.63277	-4.61124	-1.23485
KTB0015	39	Male	134	1	3.26	1.689168	-2.37336	-5.01719	-1.706
KTB0016	24	Male	336	0	2.69	1.643366	-1.77442	-6.44962	-2.4625
KTB0017	28	Female	521	1	2.69	2.822866	-2.03945	-5.25468	-2.09111
KTB0018	43	Male	750	1	2.5	1.328833	-2.10061	-4.93807	-1.37928
KTB0025	47	Female	457	1	81.04	1.74821	-1.75404	-4.92785	-1.98794
KTB0026	52	Male	323	1	2.5	1.590944	-1.90694	-4.89721	-2.50376
KTB0027	20	Male	244	1	14.16	0.948772	-2.68163	-5.28532	-2.36965
KTB0028	47	Female	804	0	6.38	-0.13899	-2.60008	-5.15254	-1.85383
KTB0029	37	Male	730	1	2.5	0.961877	-2.10061	-5.49979	-1.142
KTB0030	27	Male	101	1	12.49	2.22001	-2.58989	-4.82572	-2.20459
KTB0031	25	Male	604	0	2.5	1.564732	-1.85597	-5.92875	-1.80225
KTB0032	32	Male	295	1	2.5	1.486099	-1.6521	-5.23425	-1.85383
KTB0033	27	Female	805	1	2.5	1.51231	-1.68268	-4.97892	-1.68877
KTB0034	46	Male	264	1	2.5	1.710622	-1.81046	-5.23547	-1.11459
KTB0035	51	Female	776	0	10.33	2.285538	-1.36669	-4.72359	-1.62687
KTB0036	77	Female	170	1	9.42	1.388819	-1.99173	-5.15005	-1.28092
KTB0037	19	Female	401	1	2.5	0.477045	-2.25887	-5.47273	-1.24396
KTB0038	61	Female	2257	1	2.5	0.080156	-2.36382	-5.38731	-1.85386
KTB0039	44	Female	684	0	2.5	2.128965	-1.28572	-4.58063	-1.97399
KTB0040	28	Female	640	1	2.5	2.032424	-1.19986	-4.61859	-1.75221
KTB0042	39	Male	321	1	4.21	1.195738	-1.84862	-5.72897	-1.26244
KTB0043	29	Female	767	0	10.32	0.916842	-1.86771	-5.27343	-1.80765
KTB0044	50	Male	409	1	4.25	3.008559	-0.76098	-5.21649	-1.77993
KTB0045	28	Male	986	0	29.2	1.367366	-2.04898	-5.44426	-2.05716
KTB0046	42	Female	279	1	20.01	3.491263	-0.06451	-4.55216	-2.22349
KTB0047	25	Male	166	1	10.34	2.107512	-2.01082	-4.86534	-2.36211
KTB0048	26	Female	507	0	5.47	1.764256	-1.69597	-4.58063	-2.43603

KTB0049	37	Male	92	1	84.19	2.933471	-0.78961	-4.79891	-1.84462
KTB0050	37	Male	653	0	2.5	0.616493	-2.68821	-5.08362	-2.16805
KTB0051	31	Male	523	0	2.5	0.841755	-2.04898	-5.13107	-1.60435
KTB0052	19	Male	851	0	2.5	1.367366	-1.74368	-4.99821	-1.76145
KTB0054	19	Female	154	0	3.47	2.043151	-1.48608	-5.21649	-2.38059
KTB0055	38	Male	236	1	9.5	3.544896	0.212166	-4.24846	-2.62085
KTB0056	29	Female	285	1	18.43	2.225506	-0.79915	-5.02668	-2.15881
KTB0057	29	Female	644	1	2.5	2.032424	-1.19986	-4.75146	-1.88158
KTB0058	27	Female	155	0	3.39	1.067017	-2.22071	-5.52967	-1.84462
KTB0059	29	Female	389	0	10.53	0.562859	-2.44015	-5.0077	-1.71525
KTB0060	43	Female	384	0	2.5	2.000244	-1.73414	-4.94126	-2.19577
KTB0061	33	Female	299	1	93.31	2.193326	-2.13484	-4.91279	-2.49148
KTB0062	52	Male	261	1	2.5	0.713034	-2.14439	-5.28292	-1.74297
KTB0063	33	Male	184	1	2.5	2.300593	-1.35251	-5.02668	-1.53043
KTB0064	23	Male	166	1	29.46	2.954925	-1.66735	-4.39082	-3.18455
KTB0065	25	Female	470	0	2.5	1.528267	-1.52424	-5.47273	-1.44726
KTB0066	51	Male	299	0	2.5	0.219603	-2.93626	-5.04566	-1.56739
KTB0067	33	Female	714	0	25.64	1.238645	-2.13484	-4.77993	-2.12184
KTB0068	44	Male	447	1	2.5	1.335186	-1.69597	-4.85585	-2.32514
KTB0069	53	Female	41	1	5.53	4.82138	0.1263	-5.3019	-2.10336
KTB0070	31	Female	248	0	5.2	2.300593	-1.33343	-4.57114	-1.96475
KTB0071	39	Female	677	1	2.5	0.155243	-2.2875	-5.95674	-2.14957
KTB0072	38	Male	89	1	44.22	2.633122	-1.53378	-4.67553	-2.22349
KTB0074	41	Male	239	1	25.4	2.088955	-1.48901	-5.25468	-1.48244
KTB0075	24	Female	244	1	2.5	0.280388	-2.69182	-6.69474	-1.55465
KTB0076	49	Male	255	1	19.5	2.92771	-0.60219	-4.27421	-1.50307
KTB0077	29	Female	275	1	4.13	3.425721	-1.16282	-4.67252	-1.23485
KTB0078	20	Female	304	1	2.5	1.853055	-1.75404	-5.52022	-1.35864
KTB0079	57	Male	386	0	2.5	2.953921	-1.29534	-5.00956	-1.47212
KTB0080	49	Male	373	0	49.5	3.058766	-0.66335	-4.68274	-1.41022
KTB0081	24	Female	227	0	6.46	3.373299	-1.13224	-4.20272	-1.44117
KTB0082	30	Female	554	0	19.4	2.298643	-1.77442	-4.94828	-1.67845
KTB0083	24	Female	346	0	7.73	2.45591	-1.37688	-4.44783	-1.76098
KTB0084	25	Female	406	0	2.5	2.560755	-1.24437	-4.79508	-1.50307
KTB0085	30	Female	386	0	5.94	0.765294	-2.28409	-4.887	-1.11105
KTB0086	24	Female	259	1	5.53	3.084977	-1.03031	-4.18229	-1.26579
KTB0101	28	Male	280	1	7.08	0.372127	-2.64086	-5.41809	-1.35864
KTB0102	51	Male	565	1	56.89	2.10206	-1.4992	-4.80529	-2.04984
KTB0104	40	Male	3	1	46.38	2.993238	-1.76423	-5.00956	-2.36965
KTB0105	25	Female	253	0	2.59	1.787527	-1.63172	-5.24446	-1.61655
KTB0106	28	Female	658	0	2.5	0.56871	-2.43699	-5.54065	-1.81256
KTB0107	43	Female	499	1	50.04	1.63026	-1.84578	-5.52022	-1.80225
KTB0108	20	Female	649	1	4.71	4.209955	0.479305	-4.75146	-1.40106
KTB0109	36	Male	98	0	12.56	3.094373	-1.70551	-5.1026	-1.51195
KTB0110	28	Male	343	1	37.71	1.525416	-1.63172	-4.44783	-2.18395
KTB0111	31	Male	916	1	2.5	2.233116	-1.69288	-5.13212	-1.7713
KTB0112	27	Female	69	1	7	3.740254	-0.55122	-4.71338	-1.85383
KTB0113	34	Male	60	1	138.45	3.405449	-0.36982	-4.33388	-2.27894

KTB0115	25	Female	774	1	2.5	0.516288	-2.5797	-5.65299	-1.46181
KTB0116	29	Female	443	1	2.5	3.051466	-0.59879	-4.43827	-1.16079
KTB0117	25	Female	446	1	19.57	3.412615	-1.14244	-4.20272	-2.36965
KTB0119	47	Female	571	1	3.46	0.739083	-2.15158	-5.1934	-1.99826
KTB0120	18	Female	385	0	42.32	2.652493	-1.40746	-4.42741	-2.28712
KTB0121	38	Female	493	0	40.02	3.320877	-0.35755	-4.21293	-1.78162
KTB0122	51	Male	94	1	6.11	1.223988	-2.39622	-5.38745	-1.65782
KTB0123	31	Male	103	1	6	3.176715	-0.98954	-4.70316	-2.00858
KTB0125	32	Female	352	1	2.5	2.794024	-0.37936	-4.30541	-1.29941
KTB0126	25	Male	842	1	2.5	0.725977	-2.67144	-4.98913	-1.86415
KTB0127	34	Male	203	0	28.57	2.508332	-1.00992	-4.71338	-1.53402
KTB0128	36	Female	184	0	7.52	3.111188	-0.8978	-4.59082	-1.54434
KTB0129	26	Female	777	0	2.5	1.237094	-1.40746	-4.64188	-1.60624
KTB0130	33	Male	941	1	2.5	-0.55272	-3.57549	-5.1026	-1.4565
KTB0131	23	Female	466	0	5	2.088955	-2.10061	-5.22404	-1.57529
KTB0132	51	Male	30	1	15.59	2.944198	-1.36205	-4.30541	-1.85386
KTB0134	28	Male	61	1	32.78	4.381583	0.002271	-4.05865	-1.88158
KTB0135	36	Male	120	0	10.47	2.311749	-1.51959	-4.54996	-1.76098
KTB0136	22	Female	385	0	2.5	1.421	-1.57194	-5.56763	-1.53043
KTB0138	25	Female	332	0	2.5	1.577838	-0.92838	-4.75423	-1.92604
KTB0139	22	Male	265	0	2.5	1.407466	-2.46757	-4.91764	-1.86415
KTB0140	28	Female	84	1	2.5	1.356639	-2.08714	-4.92228	-1.67828
KTB0141	22	Female	408	0	2.5	1.15846	-1.6521	-5.17297	-1.87446
KTB0142	35	Female	399	0	2.5	2.049638	-1.8152	-4.70316	-1.2039
KTB0143	29	Female	200	0	6.05	0.345916	-2.63066	-5.05041	-2.28712
KTB0144	30	Male	164	1	27.92	1.879266	-0.82644	-4.52954	-2.31807
KTB0145	25	Female	365	0	15.97	2.80976	-1.41766	-5.21382	-1.63719
KTB0147	29	Female	570	0	2.5	0.56871	-2.7224	-5.1219	-1.56497
KTB0150	29	Female	523	1	52.25	1.971005	-1.62152	-4.66231	-1.95699
KTB0153	27	Male	345	1	2.5	3.268454	-0.276	-4.74402	-1.55465
KTB0156	25	Female	517	1	2.5	1.903703	-1.58148	-5.07413	-1.78917
KTB0157	35	Female	277	1	3.05	2.761843	-0.82777	-4.91279	-1.12383
KTB0159	48	Male	280	1	300	4.539693	-0.02117	-3.90653	-3.19496
KTB0160	26	Male	456	1	63.91	1.486099	-1.84578	-5.11169	-1.93636
KTB0162	19	Male	7	0	2.5	0.509226	-3.24157	-5.29241	-2.1126
KTB0163	40	Female	629	1	300	-0.88601	-4.50624	-4.74402	-3.31876
KTB0164	26	Female	476	1	2.5	3.019449	-0.34736	-5.24446	-1.00788
KTB0165	38	Male	380	1	25.86	1.184671	-2.1108	-5.30574	-2.25617
KTB0166	45	Female	623	0	23.92	4.290687	0.233664	-4.35591	-1.7713
KTB0167	24	Male	464	1	6.46	1.197777	-2.51854	-5.37723	-1.48244
KTB0170	28	Female	657	1	300	2.770443	-1.48901	-4.25378	-3.50445
KTB0171	41	Female	857	1	7.08	-0.91222	-3.1913	-4.94828	-1.69908
KTB0172	29	Male	482	1	7.2	1.289516	-2.00887	-5.21382	-2.56566
KTB0173	34	Female	91	1	2.5	1.223988	-2.29428	-5.00956	-1.54434
KTB0174	47	Male	283	1	34.68	-0.00793	-3.59903	-4.82572	-2.45218
KTB0175	56	Male	373	1	50.69	1.860796	-1.93449	-4.58063	-2.76871
KTB0176	25	Female	522	1	16.87	2.744232	-0.70412	-4.7338	-2.09111
KTB0177	36	Male	382	1	8.37	1.892371	-2.01906	-5.16276	-1.98794

KTB0178	28	Female	118	1	300	3.478143	-0.85702	-3.86568	-3.10212
KTB0179	24	Male	43	1	4.43	1.669577	-2.23312	-5.05041	-1.99826
KTB0181	19	Female	909	0	2.5	1.971005	-1.76423	-4.76444	-1.87446
KTB0182	36	Female	143	1	16.83	2.272432	-1.3463	-4.96871	-1.96731
KTB0183	37	Female	594	0	2.5	1.860796	-1.37159	-4.87483	-1.16079
KTB0184	34	Male	59	0	34.73	4.041682	-0.11291	-3.89632	-2.00858
KTB0186	20	Female	377	0	4.59	2.442805	-1.47882	-5.1934	-1.6475
KTB0187	32	Male	571	1	2.5	1.971005	-1.4992	-5.7347	-1.7713
KTB0189	37	Male	81	1	236.8	4.710065	0.274437	-3.46736	-2.19427
KTB0190	24	Female	14	1	42.07	4.356215	-0.4391	-3.51843	-2.58629
KTB0191	28	Female	201	0	4.54	3.268454	-0.66335	-4.57039	-2.22522
KTB0192	26	Female	145	1	300	1.667715	-2.8981	-4.36235	-2.75022
KTB0193	46	Female	242	1	16.51	2.515128	-1.16169	-4.88432	-2.42679
KTB0197	31	Female	977	1	300	1.303005	-1.93449	-4.94126	-2.22349
KTB0198	27	Male	89	1	5.2	2.676029	-1.47654	-4.88432	-2.23273
KTB0199	22	Male	256	1	2.5	2.536582	-0.98996	-4.49521	-1.706
KTB0200	45	Male	128	1	25.64	2.139692	-1.67689	-4.704	-2.17729
KTB0201	40	Male	418	1	146.42	3.56635	-0.63696	-4.59961	-2.42679
KTB0202	32	Female	205	1	25.46	1.378093	-1.76276	-4.88432	-2.21425
KTB0203	21	Female	69	1	5.58	1.957337	-1.05675	-5.42528	-2.48224
KTB0204	26	Female	192	1	300	2.654576	-1.40975	-4.23897	-2.61161
KTB0205	36	Female	541	1	6.2	2.268413	-0.94226	-4.57114	-1.66904
KTB0206	29	Female	238	0	7.37	2.225506	-0.88501	-4.63757	-1.74297
KTB0207	33	Male	393	0	2.5	4.381583	0.288492	-4.02069	-2.6763
KTB0210	31	Female	332	1	59.65	3.16361	-1.12205	-4.97892	-1.85383
KTB0211	30	Male	354	1	2.5	2.613177	-0.72451	-5.06063	-1.51339
KTB0212	44	Female	85	1	10.52	3.268454	-1.02012	-4.5806	-1.44117
KTB0213	21	Female	556	1	8.8	3.491249	-0.60219	-4.60103	-1.28643
KTB0214	39	Male	24	0	244.63	3.412615	-0.98954	-3.68184	-2.49345
KTB0216	26	Male	262	1	2.5	2.536582	-1.81046	-4.83687	-1.34561
KTB0218	29	Female	274	1	5.16	2.010321	-2.02926	-5.06063	-2.00858
KTB0219	29	Female	634	1	91.04	1.98411	-1.9681	-4.57039	-2.41092
KTB0220	32	Male	206	1	300	1.905477	-2.23312	-4.4989	-2.55535
KTB0221	32	Male	36	1	75.77	2.796654	-1.45843	-5.09126	-2.49345
KTB0222	36	Female	207	0	2.5	3.596093	-0.31678	-4.97892	-2.09111
KTB0223	21	Female	387	1	2.73	0.306599	-2.58989	-4.84615	-1.65782
KTB0224	25	Female	349	1	9.25	2.976378	-0.86593	-4.58063	-1.36409
KTB0225	31	Male	343	1	22.81	1.39436	-2.30448	-5.36702	-2.10142
KTB0226	44	Female	503	1	11.7	2.967027	-0.60219	-4.13122	-1.98794
KTB0227	31	Female	556	0	4.19	1.499205	-2.32486	-5.00956	-2.13237
KTB0228	24	Female	453	1	2.5	1.879266	-2.5797	-4.75423	-1.80225
KTB0229	25	Female	478	1	2.63	2.036532	-1.06089	-4.51932	-2.02921
KTB0230	41	Male	157	1	66.5	3.831993	-0.44929	-3.74312	-1.95699
KTB0231	33	Female	380	0	6.56	3.28156	-0.60219	-4.80529	-2.2768
KTB0232	38	Female	801	1	19.25	1.328833	-2.1108	-4.76444	-1.78162
KTB0233	48	Female	427	1	2.5	1.355044	-1.52978	-5.1934	-1.76098
KTB0234	51	Male	57	1	255.88	3.936838	0.090957	-3.60014	-2.57598
KTB0235	37	Male	408	1	2.5	2.69181	-0.65316	-4.09037	-1.81256

KTB0236	20	Male	393	1	241.37	3.137399	-0.63277	-4.51932	-1.79193
KTB0237	51	Male	98	1	10.99	1.944794	-1.1934	-5.62235	-2.28712
KTB0239	25	Female	316	1	22.17	0.68666	-2.23312	-4.81551	-2.09111
KTB0240	32	Male	18	1	300	3.448356	-0.67512	-4.02069	-2.42679
KTB0242	38	Female	822	0	2.78	1.142104	-1.59103	-5.05515	-1.36409
KTB0243	35	Female	117	0	12.27	3.030012	-0.85639	-4.77044	-1.17927
KTB0244	30	Female	616	1	2.5	-0.02104	-2.76318	-4.98913	-1.42054
KTB0245	34	Female	406	1	10.83	2.976378	-0.94226	-4.47623	-1.6136
KTB0246	30	Female	1102	1	17.97	0.280388	-2.62047	-5.07084	-1.71972
KTB0247	35	Female	327	0	2.5	2.036532	-1.98848	-4.72359	-1.67845
KTB0248	21	Female	450	0	7.26	3.451932	-0.80606	-4.69295	-1.50307
KTB0249	28	Female	457	0	2.5	2.182599	-1.88679	-5.23547	-2.19577
KTB0250	26	Male	232	0	2.78	2.062743	-1.86616	-5.2751	-2.00858
KTB0251	30	Female	127	1	100.65	1.892977	-1.74368	-4.93177	-1.81689
KTB0252	26	Female	376	1	8.97	1.45318	-1.33343	-4.8084	-0.76343
KTB0253	25	Female	564	1	2.5	1.643366	-1.88655	-4.99935	-1.33801
KTB0254	61	Female	917	0	4.21	2.10206	-1.24437	-4.89721	-1.45149
KTB0255	31	Male	396	1	5.83	1.27641	-2.20254	-4.52954	-2.24585
KTB0256	37	Male	38	1	73.31	3.320877	-0.98954	-4.0393	-2.32838
KTB0257	22	Male	580	1	2.5	2.586966	-1.40746	-5.01977	-1.73003
KTB0258	31	Female	243	0	56.81	2.180693	-1.28514	-4.99935	-2.42123
KTB0259	39	Male	1052	1	11.84	1.879266	-1.46862	-4.60103	-1.8951
KTB0260	51	Male	99	1	300	2.22001	-2.61028	-4.46826	-1.67845
KTB0261	41	Female	237	0	8.09	1.787527	-1.89674	-5.11169	-1.68877
KTB0262	26	Female	210	1	10.07	1.879266	-1.77442	-5.69384	-1.66813
KTB0263	26	Male	348	1	2.5	2.940816	-1.73365	-5.26489	-1.09042
KTB0264	39	Female	344	1	2.57	1.708894	-1.92732	-5.41809	-2.37997
KTB0265	29	Female	195	0	5.93	2.246221	-1.5094	-4.69295	-1.76098
KTB0266	32	Female	1493	0	16.72	0.699766	-2.60008	-5.02999	-1.62687
KTB0267	48	Male	19	1	21.6	3.202927	-1.18321	-5.00956	-2.20459
KTB0269	31	Female	273	1	2.5	2.469016	-0.82644	-4.77466	-2.26649
KTB0270	39	Female	743	0	2.5	-0.06036	-2.62047	-4.68274	-2.50376
KTB0271	26	Male	437	0	2.5	2.115166	-1.71326	-4.15165	-2.16332
KTB0272	30	Female	217	0	2.5	3.478143	-0.17407	-4.5806	-2.25617
KTB0273	35	Female	1022	1	5.15	1.682682	-1.98848	-5.02999	-1.57529
KTB0274	23	Male	80	1	22.54	2.154482	-2.70202	-4.54996	-2.57598
KTB0276	35	Male	176	0	57.61	2.534543	-1.4992	-4.40698	-2.49345
KTB0277	27	Female	245	1	2.5	2.088955	-1.71326	-5.15254	-2.08079
KTB0289	44	Male	599	1	18.4	2.075849	-1.71326	-4.97892	-2.24585
KTB0290	28	Female	976	1	8.23	0.974983	-2.25351	-5.02999	-1.59592
KTB0292	41	Male	237	1	2.5	0.988088	-2.17196	-5.29553	-1.97763
KTB0293	37	Female	480	0	10.38	1.368149	-2.5797	-4.50911	-0.97694
KTB0294	59	Male	418	1	5.67	1.092933	-2.07003	-5.49979	-2.11174
KTB0296	23	Female	385	0	2.5	2.495227	-0.78567	-5.13212	-1.96731
KTB0297	41	Female	886	0	2.5	-0.20452	-3.24226	-5.08105	-1.61655
KTB0298	28	Female	208	0	2.96	2.141377	-1.97829	-5.01977	-1.74035
KTB0299	26	Male	89	1	2.5	6.990431	-0.61238	-0.87321	-1.31738
KTB0300	28	Female	345	1	4.39	2.324855	-1.45843	-4.83593	-2.02921

KTB0301	33	Female	36	1	98.8	1.525416	-2.93646	-4.887	-2.26649
KTB0302	37	Male	53	1	2.5	1.525416	-2.16177	-4.7338	-2.16332
KTB0303	18	Female	267	1	3.12	1.525416	-2.14138	-4.7338	-2.00858
KTB0304	39	Male	341	0	2.5	1.800632	-1.56036	-5.20361	-1.91573
KTB0305	35	Female	216	1	89.56	1.341938	-2.55931	-5.17297	-1.76098
KTB0306	30	Female	468	1	5.25	1.538521	-1.60114	-4.95849	-1.48244
KTB0307	54	Male	978	1	13.17	0.096911	-2.4268	-5.62235	-1.69908
KTB0308	30	Female	245	1	2.5	1.643366	-1.47882	-5.10148	-1.60624
KTB0309	32	Female	197	1	5.69	2.311749	-0.98954	-5.40787	-1.61655
KTB0310	30	Female	991	1	2.5	2.049638	-2.08022	-4.5806	-1.57529
KTB0311	28	Male	225	1	2.5	1.604049	-1.90694	-5.48958	-1.18326
KTB0313	33	Female	363	0	12.53	3.451932	-0.45948	-4.33549	-0.96662
KTB0314	30	Male	125	0	2.72	1.669577	-1.77442	-5.37723	-1.74035
KTB0315	25	Female	230	1	2.5	1.643366	-1.73365	-5.85726	-1.37928
KTB0316	19	Female	115	1	9.5	2.888393	-1.60114	-4.38655	-2.15301
KTB0317	25	Female	437	0	11.93	0.280388	-3.0384	-4.91764	-1.95699
KTB0318	37	Female	1153	0	9.12	1.302621	-1.87636	-4.96871	-1.6475
KTB0319	38	Male	521	0	2.5	0.725977	-2.50834	-4.84615	-1.37928
KTB0320	21	Male	120	1	270.38	3.373268	-0.49385	-3.88783	-2.68554
KTB0321	30	Female	395	1	2.5	1.195738	-2.20163	-5.09311	-1.22548
KTB0322	28	Male	560	1	7.32	0.555605	-2.92627	-5.43851	-1.22453
KTB0323	38	Male	110	1	8.67	2.010321	-2.01906	-5.15254	-1.99826
KTB0324	36	Female	285	0	180.7	1.853055	-2.15158	-4.77466	-1.79193
KTB0325	29	Female	692	1	14.34	3.543671	-0.71432	-4.47847	-1.91573
KTB0326	28	Male	278	1	27.47	0.555605	-2.66124	-5.35681	-1.47212
KTB0328	42	Female	334	0	2.5	1.368149	-2.121	-4.59082	-1.92604
KTB0329	36	Female	487	0	2.5	1.592628	-1.66735	-4.51419	-1.5027
KTB0330	44	Male	134	1	12.15	2.37568	-1.50516	-4.58063	-2.16805
KTB0333	27	Female	NA	1	18.04	1.289516	-2.35544	-5.20361	-1.60624
KTB0334	33	Female	239	1	2.5	0.319705	-2.31467	-5.25468	-1.53402
KTB0335	35	Male	274	0	10.6	2.469016	-0.94876	-5.48958	-1.95699
KTB0336	27	Female	608	1	3.86	-0.73508	-2.71683	-6.5926	-2.74098
KTB0338	42	Male	23	1	116.57	3.405449	-1.35251	-3.86885	-2.32514
KTB0340	22	Female	434	0	13.43	3.04566	-1.22398	-3.88611	-2.11174
KTB0341	24	Female	161	0	23.69	1.51231	-2.55931	-5.06063	-2.29744
KTB0342	22	Female	511	1	2.55	2.901499	-0.57161	-4.80529	-1.7094
KTB0343	34	Female	450	1	2.5	0.843927	-3.01801	-5.45894	-2.68946
KTB0344	38	Male	21	1	300	2.508332	-1.69288	-4.82572	-3.30844
KTB0345	23	Female	213	1	5.84	2.822866	-1.11186	-4.64188	-1.60624
KTB0346	33	Male	366	1	3.7	1.302621	-2.25351	-4.66231	-1.93636
KTB0347	48	Male	9	1	2.5	2.665599	-2.21274	-4.83593	-2.51408
KTB0348	29	Female	627	1	75.25	-0.53216	-4.24121	-5.69384	-3.50445
KTB0349	39	Female	249	1	16.62	2.980132	-0.7347	-5.07084	-1.65782
KTB0350	30	Female	61	1	132.88	2.731127	-2.08022	-4.48869	-3.10212
KTB0351	39	Female	907	1	2.79	1.86616	-1.26476	-5.01977	-1.92604
KTB0352	25	Female	213	1	207.93	3.63541	-1.0507	-3.34481	-2.68946
KTB0353	44	Female	142	1	44.77	3.28156	-0.54103	-4.39677	-1.97763
KTB0354	27	Female	432	1	2.5	2.472221	-1.58148	-4.76095	-1.09611

KTB0355	26	Female	216	1	13.25	3.478143	-0.592	-4.41719	-2.32838
KTB0356	44	Female	421	1	23.53	3.51746	-0.33716	-4.6521	-2.18395
KTB0358	55	Female	242	1	3.21	2.45591	-1.73365	-5.38745	-2.24585
KTB0359	22	Male	146	0	26.73	1.74821	-1.87636	-5.1219	-2.22522
KTB0360	48	Female	115	1	61.54	2.31132	-1.82	-4.82738	-1.63208
KTB0361	49	Male	507	0	3.17	0.791505	-2.92627	-5.10148	-1.79193
KTB0362	46	Male	876	1	3.68	1.459888	-2.17196	-4.57039	-2.4625
KTB0363	36	Male	335	1	9	1.197777	-1.77442	-5.59171	-2.61724
KTB0364	30	Female	425	0	4.79	1.45318	-2.32566	-5.05515	-1.4103
KTB0365	28	Female	178	1	2.5	1.871523	-1.95357	-4.93177	-1.40106
KTB0366	33	Male	294	1	2.5	1.989517	-1.40021	-4.97923	-1.38257
KTB0367	43	Female	296	1	7.22	2.849077	-0.57161	-5.10148	-1.57529
KTB0368	40	Female	578	0	2.5	1.604049	-1.43804	-4.68274	-1.56497
KTB0369	23	Female	519	0	8.58	1.74821	-1.55017	-4.74402	-1.74035
KTB0370	32	Male	199	0	3.59	1.174284	-2.72637	-4.93177	-1.77069
KTB0371	35	Female	94	0	3.13	5.518618	-3.2034	-2.03719	-2.15881
KTB0372	59	Male	42	1	2.5	3.008559	-1.72459	-4.92228	-1.81689
KTB0373	41	Female	276	1	3.08	1.63026	-1.8152	-5.84704	-2.18395
KTB0374	29	Female	74	1	2.5	2.128965	-1.5147	-4.42878	-1.85386
KTB0375	27	Female	385	1	4.51	1.931688	-1.58075	-4.43762	-1.97763
KTB0376	31	Male	644	1	153.33	1.538521	-1.75404	-4.96871	-1.81256
KTB0377	23	Female	618	1	11.14	2.639388	-1.58075	-4.92785	-2.43155
KTB0379	26	Female	740	1	2.5	1.560447	-2.45923	-5.25445	-1.91854
KTB0380	32	Female	170	0	133.27	1.86616	-2.67144	-4.77466	-2.99895
KTB0381	29	Male	212	1	138.42	2.954925	-1.06629	-4.24846	-2.66706
KTB0382	41	Female	313	0	6.64	0.293494	-2.63066	-4.75423	-2.17364
KTB0383	26	Female	252	0	2.5	0.509226	-3.27973	-5.82387	-2.13108
KTB0384	33	Male	562	1	5.24	0.424549	-3.00782	-4.60103	-1.87446
KTB0385	27	Male	487	1	2.82	1.560447	-1.48608	-5.12158	-1.2532
KTB0386	26	Female	87	1	168.65	5.247393	0.55985	-3.8044	-3.36003
KTB0387	35	Female	27	1	76.16	3.504354	-1.25456	-4.33549	-3.05053
KTB0388	31	Female	225	1	2.5	1.145355	-2.28409	-5.43851	-3.00927
KTB0389	39	Female	688	1	2.5	1.092933	-1.83558	-4.79508	-1.32769
KTB0390	66	Female	165	0	28.99	3.556777	-0.57161	-4.38655	-2.20459
KTB0391	42	Female	436	1	19.78	2.757338	-1.38708	-5.20361	-1.90541
KTB0392	40	Male	373	0	2.97	1.315727	-1.94771	-5.56107	-2.14269
KTB0393	23	Male	24	0	9.54	1.971005	-2.13119	-4.83593	-2.72041
KTB0394	33	Female	252	0	6.89	1.420571	-1.93752	-4.99935	-2.13237
KTB0395	25	Female	259	0	23.1	0.380505	-2.90764	-5.18801	-2.66706
KTB0396	39	Male	273	0	2.5	1.185011	-2.1253	-4.52369	-2.09412
KTB0398	29	Female	138	0	5.02	3.255274	-0.81823	-4.68502	-1.55815
KTB0400	28	Male	217	1	5.5	1.51231	-1.88655	-4.85636	-1.97763
KTB0401	54	Male	150	1	4.65	2.940816	-0.4289	-4.76444	-1.93636
KTB0403	19	Female	796	1	38.4	3.491249	-0.37794	-4.3457	-1.90541
KTB0404	18	Female	316	0	2.5	2.92771	-0.65316	-4.97892	-2.04984
KTB0405	35	Male	272	0	2.5	2.115166	-1.40746	-4.77466	-1.5856
KTB0406	37	Female	210	1	106.64	4.34311	0.090957	-3.4163	-2.65851
KTB0407	27	Male	258	0	105.96	2.416593	-1.2036	-4.92785	-3.0918

KTB0408	58	Female	310	1	2.5	3.373299	-0.61238	-4.69295	-2.14269
KTB0409	32	Female	667	1	2.5	0.044488	-3.26265	-4.67252	-2.15301
KTB0410	23	Male	580	1	2.5	0.096911	-2.62047	-5.06063	-1.33801
KTB0411	34	Female	691	1	2.5	1.184671	-2.10061	-6.44962	-2.60693
KTB0413	25	Female	290	0	47.85	1.001194	-2.44718	-4.887	-2.63788
KTB0414	23	Female	520	0	5.47	1.328833	-1.87636	-5.2751	-1.92604
KTB0415	36	Male	317	1	101.06	0.45076	-2.92627	-4.82572	-2.71009
KTB0416	45	Female	513	0	4.09	2.796654	-0.8978	-4.90743	-1.43086
KTB0417	55	Female	1601	0	10.67	0.267283	-2.50834	-4.94828	-1.28643
KTB0418	35	Male	71	1	95.51	0.909455	-2.75298	-5.1934	-2.58629
KTB0419	24	Female	272	1	3.36	4.002365	-0.02117	-4.53975	-1.81256
KTB0420	44	Female	77	0	23.14	3.190913	-0.86593	-4.93177	-2.37135
KTB0422	41	Female	609	0	3.5	1.421	-2.18255	-5.16903	-1.56739
KTB0424	21	Male	728	1	84.48	1.077744	-2.1253	-4.86534	-2.60237
KTB0425	27	Male	439	0	77.89	3.189821	-0.54103	-4.33549	-2.2149
KTB0426	27	Female	239	1	12.31	0.713034	-2.47831	-4.44776	-1.58587
KTB0428	29	Female	1005	0	3.69	-0.24383	-3.0384	-4.91764	-1.63719
KTB0430	19	Male	86	1	75.23	1.604049	-3.11994	-4.29463	-2.54503
KTB0431	57	Female	447	1	24.77	3.189821	-0.79586	-4.23335	-1.99826
KTB0432	43	Female	197	0	2.5	1.446783	-2.32486	-5.23425	-1.94668
KTB0433	33	Female	514	0	2.96	0.434138	-2.48785	-5.0077	-2.03868
KTB0434	37	Female	393	1	79.34	2.80976	-0.81625	-4.81551	-2.60693
KTB0435	42	Male	436	0	2.5	0.552133	-1.93449	-5.64356	-1.32713
KTB0436	39	Male	269	1	55.52	-0.1022	-2.71683	-5.14056	-1.83538
KTB0437	33	Male	25	1	66.12	1.002656	-3.58503	-5.92827	-2.56541
KTB0440	34	Male	112	0	8.09	2.27914	-2.24933	-5.22598	-1.81689
KTB0441	23	Female	401	1	10.56	3.150504	-0.70412	-4.22314	-1.8951
KTB0442	29	Female	510	1	3.13	0.605766	-2.3829	-5.62457	-2.07564
KTB0443	29	Female	307	1	2.5	1.324459	-2.85994	-5.28292	-1.95551
KTB0444	37	Female	68	1	5.75	1.94661	-1.84862	-5.52018	-2.15881
KTB0445	41	Female	344	1	25.29	3.019285	-1.70551	-4.84636	-1.80765
KTB0446	30	Female	214	0	2.5	1.132249	-2.52873	-5.83683	-1.8951
KTB0447	44	Female	160	1	33.64	3.448356	-0.50339	-4.61859	-1.6598
KTB0449	50	Male	540	1	91.04	0.713034	-2.42107	-5.38731	-1.78917
KTB0450	37	Male	83	1	100.71	3.347088	-0.276	-4.08016	-2.30775
KTB0452	29	Male	237	1	70.49	2.586966	-1.2036	-4.57039	-2.87515
KTB0453	36	Female	55	1	163.82	3.072919	-0.87547	-3.66955	-2.71326
KTB0454	24	Female	251	0	4.15	2.835971	-1.0507	-4.7338	-2.08079
KTB0455	39	Male	182	0	2.5	1.270825	-2.58326	-5.54865	-2.21425
KTB0456	35	Female	417	0	2.76	1.617155	-1.6623	-5.41809	-2.07047
KTB0457	24	Male	169	1	5.05	1.399546	-2.01082	-4.94126	-2.17729
KTB0458	22	Female	254	1	2.5	0.385233	-2.55931	-5.65299	-2.67914
KTB0459	37	Female	85	1	172.11	3.111188	-1.06089	-3.82483	-2.95769
KTB0460	38	Male	613	0	2.5	1.027405	-1.82539	-4.84615	-2.03953
KTB0461	56	Female	181	1	72.67	-0.64926	-3.94758	-4.8084	-2.84263
KTB0462	51	Male	264	1	100.57	2.141377	-1.17302	-4.30485	-2.31807
KTB0463	39	Male	303	0	2.5	1.839949	-1.59094	-5.01977	-1.96731
KTB0464	29	Male	295	0	2.5	2.064605	-1.62919	-4.96024	-2.24198

KTB0465	26	Female	73	1	129.48	3.648515	-0.00078	-4.02909	-2.98863
KTB0466	26	Female	297	1	2.5	2.088955	-1.90694	-4.96871	-1.86415
KTB0468	42	Male	206	1	2.5	0.959749	-2.53555	-5.28292	-1.58587
KTB0469	33	Female	227	0	2.65	1.839343	-2.29704	-5.48222	-1.8631
KTB0470	56	Male	531	1	23.9	1.617155	-2.4268	-5.89811	-2.87515
KTB0471	25	Male	554	0	2.5	1.800632	-1.27495	-4.98913	-1.7094
KTB0472	34	Female	93	1	227.63	2.233116	-2.36564	-4.44783	-3.44256
KTB0473	30	Female	138	1	8.82	1.48536	-2.04898	-5.52967	-1.75221
KTB0475	49	Female	204	1	4.42	1.796436	-1.67689	-5.51069	-1.67828
KTB0478	35	Male	16	1	130.4	3.63541	-0.61238	-3.43672	-3.11243
KTB0481	36	Female	341	1	171.51	3.56635	-0.41752	-3.90681	-2.58389
KTB0482	35	Male	28	1	101.53	3.619984	-1.09491	-4.51419	-1.96475
KTB0484	35	Female	267	0	5.31	1.957337	-1.31435	-5.19751	-2.29742
KTB0485	33	Male	48	0	2.5	1.034837	-2.41152	-5.25445	-2.01095
KTB0486	47	Male	357	0	6	2.901499	-0.51045	-4.51932	-2.06016
KTB0487	30	Female	70	1	300	4.526587	0.447724	-3.48779	-2.77199
KTB0488	41	Male	274	0	3.61	2.22001	-0.95896	-5.2751	-2.03953
KTB0489	26	Female	140	1	2.5	3.062192	-1.35251	-4.6091	-1.62284
KTB0490	25	Male	355	1	31.87	1.826844	-2.03945	-4.53975	-2.26649
KTB0491	27	Female	306	0	3.2	2.45591	-1.61133	-5.07084	-1.66813
KTB0492	48	Male	192	1	90.11	2.233116	-2.07003	-4.39677	-2.88547
KTB0494	41	Male	192	1	300	-1.26607	-4.91397	-5.75512	-3.22591
KTB0499	29	Female	350	1	2.65	2.469016	-1.15263	-5.1219	-1.60624
KTB0500	26	Female	386	1	9.38	3.582988	-0.38813	-4.57039	-1.67845
KTB0501	35	Male	700	1	133.43	0.56871	-2.49815	-4.56018	-1.84351
KTB0502	31	Female	873	1	40.74	2.901499	-1.0405	-4.3457	-2.11174
KTB0503	26	Female	678	0	2.89	2.718021	-0.76528	-4.66231	-2.03953
KTB0504	32	Female	334	1	2.5	3.04566	-0.79586	-4.3457	-1.88478
KTB0505	31	Male	308	1	25.58	4.054788	-0.37794	-4.3457	-2.15301
KTB0506	23	Female	60	0	26.83	3.530565	-1.77442	-4.76444	-2.99895
KTB0507	40	Male	214	1	16.57	2.875288	-0.53084	-4.39677	-1.94668
KTB0509	27	Female	249	0	6.03	3.150504	-1.0405	-4.887	-1.5237
KTB0510	30	Male	683	0	2.5	0.935666	-2.85492	-4.95849	-1.55465
KTB0511	34	Male	302	1	77.09	0.110016	-3.1811	-5.32617	-2.56566
KTB0512	37	Female	288	1	9.91	2.967027	-0.93857	-4.54996	-1.60624
KTB0513	35	Female	154	1	300	1.735105	-1.72346	-4.50911	-2.43155
KTB0516	33	Female	46	1	120.61	2.744232	-2.89569	-4.86657	-2.65851
KTB0517	33	Female	730	0	2.5	-0.29626	-2.95685	-5.05041	-1.91573
KTB0518	35	Female	586	1	2.5	1.171566	-2.19235	-5.0402	-1.80225
KTB0519	42	Male	146	1	33.83	3.216032	-0.72451	-4.3457	-2.18395
KTB0520	43	Male	158	1	118.63	2.364954	-2.29704	-4.59012	-2.27894
KTB0521	26	Male	192	1	92	4.434849	0.141924	-3.46736	-2.42123
KTB0523	31	Female	76	1	73.4	2.560755	-1.67249	-4.69295	-2.02921
KTB0524	22	Female	524	0	8.38	0.584313	-2.79315	-4.63757	-2.03868
KTB0525	32	Female	486	0	24.61	1.817889	-1.61011	-4.95075	-1.97399
KTB0526	50	Female	776	0	5.38	2.021698	-1.31435	-4.64706	-2.41755
KTB0527	44	Female	278	1	2.5	3.126553	-0.6942	-4.51419	-1.81689
KTB0528	25	Female	169	0	9.1	1.614081	-1.95357	-5.05515	-1.83538

KTB0531	37	Male	93	1	20.72	2.107512	-1.31435	-4.29591	-1.42878
KTB0532	42	Male	93	1	86.95	3.083646	-1.40975	-4.43827	-2.25122
KTB0533	27	Female	321	1	2.88	3.126553	-0.88501	-4.9033	-1.92779
KTB0534	59	Male	NA	1	67.84	3.072919	-1.18078	-4.62808	-2.07564
KTB0535	44	Male	17	0	3.15	-1.32505	-3.81401	-4.96024	-2.79643
KTB0536	29	Female	12	1	125.78	3.695071	-0.97088	-4.00171	-2.98125
KTB0537	31	Male	428	1	6.19	0.788121	-2.02036	-4.93177	-2.01095
KTB0538	41	Male	330	1	13.18	1.603354	-1.89633	-5.11209	-1.9093
KTB0539	31	Male	154	0	15.8	2.654576	-1.29526	-4.94126	-2.03868
KTB0540	33	Male	43	1	238.43	4.05978	-0.0836	-4.10611	-2.86111
KTB0541	22	Female	350	0	3.94	1.538521	-2.09042	-5.31596	-2.47281
KTB0543	29	Female	530	0	2.5	1.302621	-1.74384	-4.99935	-1.6475
KTB0544	27	Female	230	1	54.67	2.193799	-1.67249	-4.7338	-3.06085
KTB0545	33	Male	493	0	2.5	1.260098	-1.89633	-4.64706	-1.99247
KTB0546	30	Male	264	1	5.26	1.689168	-1.60057	-5.16903	-1.52119
KTB0547	33	Female	1214	1	2.5	0.254177	-2.78356	-4.95849	-1.5856
KTB0549	39	Male	215	1	232.45	-1.52818	-4.62856	-4.81551	-3.17433
KTB0550	30	Female	491	1	103.35	2.718936	-2.0776	-5.36833	-2.36211
KTB0551	23	Female	216	1	11.33	2.351066	-1.67249	-4.95849	-2.2768
KTB0552	60	Female	291	1	14.2	3.111188	-0.4289	-4.54996	-2.89579
KTB0553	23	Male	164	0	2.5	2.311749	-1.88655	-4.80529	-2.4625
KTB0554	48	Female	102	1	11.09	1.635535	-2.53555	-4.8084	-1.94627
KTB0555	53	Male	58	1	106.52	3.858204	-0.34736	-4.78487	-2.77199
KTB0556	34	Female	110	0	2.5	1.152831	-1.96311	-4.8084	-1.88158
KTB0557	33	Male	478	1	3.61	2.246221	-0.93857	-5.09126	-2.32838
KTB0558	34	Male	687	0	2.5	-0.92816	-2.65958	-5.33037	-1.35485
KTB0559	45	Female	523	1	14.26	1.98411	-1.59094	-4.90743	-1.87446
KTB0560	21	Female	368	1	3.58	2.351066	-0.83664	-4.69295	-1.76098
KTB0561	42	Male	300	1	4.21	4.91792	0.317114	-4.63757	-2.16805
KTB0562	44	Female	284	1	138.8	3.582988	-0.70412	-4.40698	-2.83389
KTB0564	30	Male	288	1	27.6	1.092933	-2.1108	-5.6632	-2.5244
KTB0565	42	Female	111	0	5.3	1.826844	-1.87636	-4.78487	-2.08079
KTB0566	33	Male	740	0	5.79	1.525416	-1.88655	-6.78666	-2.79262
KTB0567	41	Female	39	1	44.44	2.469016	-1.63172	-5.01977	-2.3387
KTB0568	36	Male	59	1	108.73	2.849077	-0.5818	-3.98824	-3.01958
KTB0569	32	Female	16	1	257.7	2.686756	-2.44015	-5.16903	-3.02745
KTB0570	32	Female	87	1	2.5	1.732075	-1.60057	-4.96974	-2.5192
KTB0571	37	Male	257	1	300	3.748705	-1.24756	-4.38133	-2.75022
KTB0572	35	Male	230	1	300	4.875013	0.40298	-3.83088	-2.13108
KTB0573	40	Male	50	1	122.62	3.16946	-0.87547	-4.91279	-2.18653
KTB0574	32	Female	81	1	34.32	2.997832	-1.55286	-4.704	-2.53768
KTB0575	49	Male	96	1	149.16	4.016873	-0.18854	-3.93528	-2.49148
KTB0576	18	Male	462	1	116.78	1.925157	-1.94403	-5.207	-2.473
KTB0577	28	Female	696	0	2.5	0.62722	-3.06029	-5.44426	-1.99247
KTB0578	24	Female	283	1	4.79	2.354227	-2.13484	-5.32088	-2.19577
KTB0579	39	Female	136	1	229.34	3.126553	-1.24756	-4.39082	-2.38983
KTB0580	19	Female	1008	0	34.02	2.74039	-1.84862	-5.05515	-2.02019
KTB0582	31	Male	311	1	13.26	2.010971	-1.74368	-5.42528	-2.35287

KTB0583	40	Male	1042	0	9.59	0.326871	-2.57372	-5.207	-1.77069
KTB0584	19	Female	494	0	2.6	1.968064	-1.61011	-5.15954	-2.32514
KTB0585	37	Female	97	1	5.1	3.319634	-1.01858	-4.68502	-2.01095
KTB0586	43	Female	109	1	98.22	3.051466	-1.73414	-4.59012	-2.83339
KTB0587	49	Male	242	0	15.13	1.646261	-2.44969	-5.37782	-2.26046
KTB0588	38	Male	44	1	34.81	3.53417	-1.41929	-4.64706	-2.70402
KTB0589	27	Female	227	0	51.25	2.697483	-0.80869	-4.704	-2.27894
KTB0591	25	Male	29	1	280.8	3.330361	-2.1253	-4.25795	-3.74824
KTB0592	36	Male	274	1	9.95	-0.27383	-2.55464	-5.15005	-2.29742
KTB0593	28	Female	375	1	29.19	2.075331	-2.05852	-5.05515	-2.33438
KTB0594	36	Female	19.15	1	7.85	3.105099	-1.35251	-4.72298	-2.63933
KTB0596	39	Female	183	1	149.82	2.815477	-1.22848	-4.74196	-3.14758
KTB0597	46	Female	451	1	300	-0.16656	-3.56595	-6.00419	-3.18455
KTB0598	35	Male	32	1	109.16	4.338676	0.021352	-4.04916	-2.80567
KTB0599	22	Female	523	1	8.14	2.611669	-1.59103	-4.82738	-2.58389
KTB0601	32	Male	40	1	128.27	3.523443	-0.60833	-4.13458	-2.61161
KTB0602	50	Female	417	1	9.98	3.083646	-1.22848	-5.08362	-1.92779
KTB0603	35	Female	325	1	7.36	2.086058	-1.38113	-5.67203	-2.71326
KTB0604	24	Female	276	0	2.5	3.223094	-0.66558	-4.83687	-1.82614
KTB0605	40	Male	287	1	28.97	2.139692	-1.49562	-5.03617	-2.25122
KTB0606	42	Male	489	1	2.5	1.871523	-1.24756	-4.66604	-1.9093
KTB0607	26	Male	425	0	2.5	2.139692	-1.65781	-4.88432	-2.32514
KTB0608	38	Male	393	1	18.84	1.813738	-1.5094	-4.99935	-1.93636
KTB0609	27	Female	522	1	40.52	1.486099	-2.07003	-5.30574	-2.16332
KTB0610	59	Male	514	0	197.86	1.119144	-2.18216	-4.74402	-2.3387
KTB0611	48	Male	476	1	93.84	2.967027	-0.99973	-4.94828	-2.22522
KTB0612	32	Male	153	1	38.9	2.332773	-1.86771	-4.62808	-2.53768
KTB0613	45	Female	149	0	4.45	3.04566	-0.72451	-5.02999	-1.94668
KTB0614	30	Female	380	0	195.6	2.442805	-1.0507	-5.09126	-2.31807
KTB0615	26	Female	43	1	81.07	4.552799	0.570044	-4.10058	-3.04022
KTB0616	54	Female	306	1	2.5	2.075849	-1.47882	-4.99935	-1.98794
KTB0617	32	Female	45	1	27.73	3.936838	0.13173	-4.56018	-2.00858
KTB0619	40	Male	160	1	14.76	2.107512	-2.71683	-5.15005	-3.06441
KTB0620	38	Female	264	1	11.55	2.835971	-1.13224	-4.44783	-0.85314
KTB0621	39	Male	471	1	2.5	2.547308	-0.97088	-5.02668	-2.05716
KTB0622	29	Female	275	1	25.06	1.368149	-1.85597	-5.63256	-2.47281
KTB0623	32	Male	272	0	2.5	1.74821	-2.01906	-5.63256	-2.81326
KTB0624	56	Female	189	0	182.41	2.914604	-1.62152	-4.80529	-1.85383
KTB0625	33	Male	167	0	2.5	2.922745	-1.15215	-5.18801	-1.91854
KTB0626	30	Female	1038	0	2.5	0.660449	-2.46757	-5.43851	-1.36896
KTB0627	38	Male	522	0	2.5	2.154482	-1.0405	-6.06152	-2.4625
KTB0629	31	Female	299	0	3.1	2.508332	-1.43804	-5.08105	-1.43086
KTB0631	29	Female	274	0	3.12	1.617155	-2.1108	-5.60193	-2.66883
KTB0632	40	Male	388	0	2.7	1.800632	-1.18321	-5.23425	-1.68877
KTB0633	36	Female	246	1	2.5	3.189821	-0.41871	-4.59082	-2.12206
KTB0634	59	Male	380	0	2.5	1.001194	-2.2637	-5.20361	-1.79193
KTB0635	50	Male	1092	0	2.5	-0.03414	-3.0384	-5.34659	-1.79193
KTB0636	40	Male	125	0	39.6	2.075331	-1.37159	-5.29241	-1.92779

KTB0638	60	Male	248	1	2.5	3.504354	-0.46968	-4.21293	-2.13237
KTB0640	41	Male	773	1	2.5	1.74821	-1.56036	-4.94828	-1.79193
KTB0641	30	Female	363	1	36.57	5.003734	0.574712	-2.90082	-2.02019
KTB0642	26	Male	154	1	175.09	3.976154	0.029797	-4.33549	-2.54503
KTB0643	26	Female	226	0	7.86	3.242243	-1.3565	-4.61124	-2.30775
KTB0645	51	Female	243	1	2.5	1.682682	-1.8152	-4.67252	-2.31807
KTB0646	47	Female	275	1	2.5	4.356215	0.080764	-4.35591	-1.92604
KTB0647	32	Female	184	0	2.5	0.725977	-2.28409	-5.2751	-1.67845
KTB0648	45	Female	NA	1	300	1.014299	-2.30448	-5.2751	-2.2149
KTB0649	45	Male	450	0	2.5	1.98411	-1.31572	-4.93807	-1.6475
KTB0650	54	Female	37	1	39.62	3.973966	-0.73236	-4.68502	-2.63009
KTB0651	28	Female	326	1	9.77	2.888393	-1.64191	-4.7338	-2.79262
KTB0652	30	Male	199	1	102.87	0.660449	-2.46757	-5.30574	-1.28643
KTB0653	42	Female	163	1	45.54	1.315727	-2.2637	-5.61214	-1.67845
KTB0654	35	Male	372	1	2.5	1.892371	-1.39727	-4.91764	-1.57529
KTB0655	38	Male	217	1	50.73	3.307771	-0.65316	-4.72359	-3.14338
KTB0656	24	Female	280	0	209.4	3.019285	-1.12353	-4.76095	-2.5192
KTB0657	34	Female	129	1	16.7	1.15846	-2.15158	-5.78576	-2.11174
KTB0658	26	Female	521	1	85.39	1.761316	-1.3565	-5.21382	-2.06016
KTB0660	48	Male	176	1	5.25	0.92256	-2.46757	-5.94917	-2.69978
KTB0661	36	Male	513	1	2.5	3.098082	-0.95896	-4.46826	-1.56497
KTB0662	40	Male	624	1	101.58	2.023427	-1.42785	-4.35591	-3.45287
KTB0663	26	Female	316	0	2.5	-0.55837	-3.29323	-5.2751	-1.94668
KTB0664	26	Male	5	0	99.81	2.45591	-1.6623	-4.75423	-1.92604
KTB0665	24	Female	945	1	2.5	3.51746	-0.85702	-5.05041	-2.15301
KTB0666	36	Male	247	1	51.41	1.27641	-2.98743	-4.99935	-2.47281
KTB0670	41	Male	521	1	3.22	1.142104	-0.79915	-5.35884	-1.42878
KTB0671	39	Female	104	1	21.57	2.622396	-1.49562	-4.89381	-2.18653
KTB0672	20	Female	446	0	30.42	3.040739	-1.39067	-4.43827	-2.0664
KTB0673	41	NA	NA	0	2.5	-0.50982	-3.9285	-6.05164	-1.4565
KTB0674	24	Female	428	0	2.5	2.300593	-1.64827	-5.15005	-2.08488
KTB0675	28	Female	409	0	201.47	3.823792	-1.23802	-4.52369	-2.39907
KTB0676	28	Male	257	0	4.28	2.579489	-1.467	-4.97923	-1.53967
KTB0677	32	Female	79	0	2.57	4.102687	-0.75144	-4.44776	-2.23273
KTB0678	31	Female	460	0	21.56	1.721349	-1.76276	-4.65655	-1.68752
KTB0679	45	Male	NA	1	10.84	2.901291	-0.66558	-4.76095	-2.07564

GAS6	SEPTIN4	CD1C	BLK	GBP2	SERPING1	TUBGCP6	TRMT2A	SDR39U1	FCGR1A
-7.9645	-7.17581	-5.61899	-6.48835	-1.40227	-4.97529	-8.41753	-9.42433	-7.76694	-2.73919
-6.77487	-6.27696	-6.4558	-6.65296	-1.28045	-2.90042	-6.69984	-7.51607	-7.71619	-2.53576
-7.79016	-8.25029	-5.90789	-6.98217	-1.5647	-4.73238	-7.45197	-8.43002	-7.73649	-3.26811
-8.36446	-7.67172	-5.99755	-7.16615	-1.55455	-4.22632	-7.26902	-7.7772	-7.67559	-3.23759
-7.93373	-6.56625	-5.86804	-7.6987	-1.77788	-3.59879	-7.08607	-8.28942	-7.9395	-3.5936
-8.09782	-7.0415	-5.3002	-7.06932	-1.74743	-6.91859	-6.21198	-7.5462	-7.8989	-3.35965
-7.12355	-6.13232	-7.12326	-7.96013	-1.44288	-2.45508	-7.27918	-8.33963	-7.67559	-2.44422
-8.27837	-7.71588	-4.63173	-7.13977	-1.69658	-3.8873	-7.5915	-8.2333	-8.23896	-3.50758
-7.45173	-7.15514	-7.41216	-6.8563	-1.04696	-3.72025	-6.9641	-7.90777	-8.33535	-2.07804
-8.53881	-6.95884	-6.55542	-7.55345	-1.63576	-3.58867	-7.62475	-8.2392	-7.59439	-3.56308
-8.64136	-7.83702	-6.0872	-7.52441	-1.62561	-4.6919	-6.54739	-7.95798	-7.74664	-3.50205
-7.80041	-7.83702	-6.0872	-8.02791	-2.20426	-5.08663	-7.63492	-8.27937	-8.041	-3.55291
-7.39019	-7.0105	-7.02364	-7.97949	-2.21441	-3.91255	-7.75688	-8.79159	-8.13235	-3.57325
-8.25165	-8.26062	-6.00751	-6.88535	-2.29562	-4.8842	-8.29557	-8.90207	-8.61955	-4.80401
-8.17987	-7.0105	-6.12705	-7.85362	-1.64591	-3.41661	-7.20804	-8.29946	-7.99025	-3.35965
-8.0568	-7.41343	-5.94773	-7.61155	-0.85407	-3.1737	-7.00476	-7.96803	-7.53349	-1.14226
-7.92348	-6.63857	-7.56159	-9.73207	-1.76773	-6.39228	-10.0133	-9.18328	-9.45186	-2.14924
-7.71837	-6.74188	-5.29024	-7.35012	-1.51394	-4.0745	-7.5841	-7.68681	-7.31019	-2.96296
-8.04654	-5.8637	-5.93777	-7.72774	-1.15863	-3.42673	-8.14311	-8.97237	-7.8989	-1.89495
-8.46702	-6.19431	-6.50561	-7.3598	-1.51394	-4.09474	-7.95	-8.73133	-9.80711	-1.59998
-7.94399	-7.72338	-5.86804	-7.44694	-1.46318	-4.41862	-7.49262	-8.43002	-8.4876	-2.83073
-8.27216	-6.03934	-6.28645	-7.26297	-1.3109	-3.27491	-7.40115	-8.01824	-8.37595	-2.38319
-7.69786	-5.38845	-5.71861	-7.55345	-0.97589	-2.66763	-7.62475	-7.64664	-9.2184	-2.35267
-7.8722	-4.43795	-6.03739	-6.64328	-0.71195	-1.42271	-7.14705	-7.88768	-7.28989	-0.90832
-7.70811	-6.6489	-6.31633	-7.15646	-1.99107	-3.94292	-7.90934	-8.10863	-7.8989	-3.72583
-7.74914	-8.93217	-5.91785	-7.05963	-1.51394	-5.44087	-6.30345	-7.67677	-7.58424	-3.69531
-6.6518	-4.46895	-7.193	-7.43726	-1.07741	-1.89841	-7.9195	-8.50033	-8.6297	-1.15243
-6.70308	-7.12415	-7.53171	-9.94509	-2.14335	-3.7911	-8.21426	-9.41428	-8.5891	-2.71885
-7.64658	-5.36779	-5.91785	-6.82725	-0.9962	-2.4146	-6.03919	-7.23486	-7.62484	-2.57645
-7.55428	-6.40094	-6.42592	-8.01823	-1.23984	-2.91054	-7.25885	-8.33963	-8.11205	-2.21027
-8.47547	-7.55932	-5.80783	-8.50702	-0.56753	-4.66609	-6.96635	-8.34289	-8.56725	-2.04864
-9.39001	-7.28945	-5.94773	-6.7982	-1.16878	-4.37813	-7.00476	-8.86189	-7.42184	-2.8409
-7.70582	-7.46722	-5.78738	-8.37029	-0.95353	-3.93597	-6.95673	-7.46611	-7.60169	-2.65333
-7.51326	-7.0105	-6.9041	-9.16079	-1.80834	-6.34167	-8.03131	-8.7715	-8.244	-2.57645
-7.75939	-8.27095	-5.67876	-6.56582	-1.96062	-4.63117	-6.72017	-8.01824	-7.31019	-3.8072
-8.92851	-6.82454	-5.75845	-6.90471	-1.2906	-4.36801	-7.36049	-7.95798	-8.19325	-2.82056
-7.54403	-6.77288	-5.39982	-6.86598	-1.43272	-3.63928	-6.46607	-7.79729	-7.37109	-2.52559
-8.29268	-6.42161	-5.76842	-6.99186	-1.2703	-3.83158	-7.01492	-8.10863	-7.77709	-2.30181
-8.18451	-9.14335	-5.48057	-7.6297	-2.37208	-5.07496	-7.50494	-8.21337	-7.75618	-4.22746
-8.02603	-8.39493	-5.07107	-6.22692	-2.47836	-5.41051	-7.03525	-7.75711	-7.8989	-4.50903
-7.93373	-7.78537	-4.79213	-6.37216	-2.09259	-6.77689	-7.34017	-8.19903	-7.8177	-3.37999
-8.04372	-8.53553	-5.50102	-7.02583	-2.39138	-5.86349	-7.84156	-8.66172	-8.58656	-4.16987
-7.13381	-5.61575	-7.85049	-8.39585	-1.46318	-2.72836	-7.72639	-8.45011	-9.52291	-1.68135
-8.13758	-6.61074	-6.03283	-7.56134	-0.94388	-3.89703	-7.76462	-8.38275	-8.13275	-1.10801
-7.61581	-8.45692	-6.12705	-7.33075	-2.36669	-5.28905	-7.6959	-8.45011	-7.88875	-4.50903
-8.71951	-7.37513	-5.7976	-7.2651	-1.47463	-6.72989	-6.49508	-7.81483	-7.35064	-3.81473
-7.82092	-5.22315	-10.0123	-9.24793	-1.92001	-2.6069	-8.11262	-9.39419	-10.071	-1.21346

-7.44147	-7.13448	-5.90789	-8.01823	-1.57485	-3.11297	-7.50279	-8.20907	-8.37595	-2.66799
-6.40567	-3.92138	-8.7172	-9.2092	-1.2906	-1.81744	-7.43164	-9.17324	-8.4267	-0.99986
-7.15432	-6.02901	-6.25656	-7.08868	-1.32105	-3.59879	-7.16738	-8.34968	-8.0004	-2.09838
-8.18451	-8.43422	-4.7749	-6.31942	-1.86063	-5.02628	-7.24526	-8.2333	-7.57272	-3.23883
-8.12859	-6.72122	-5.81823	-7.74711	-1.78804	-4.81335	-7.40115	-7.90777	-7.74664	-3.31897
-7.66709	-6.48359	-5.9577	-7.06932	-1.24999	-3.63928	-6.41526	-7.91781	-7.58424	-2.93245
-7.45173	-4.65492	-10.5303	-10.1678	-1.41242	-2.52593	-8.45819	-9.93654	-10.9642	-0.51163
-8.08757	-6.69023	-6.87421	-8.33776	-1.41242	-3.90243	-7.84836	-8.46016	-8.041	-2.08821
-7.92348	-2.75392	-6.61519	-7.56314	-0.09269	-1.21016	-7.89918	-8.51037	-8.3861	0.169861
-8.34395	-6.97951	-6.11709	-8.87999	-1.61546	-3.88219	-7.49262	-8.19903	-8.07145	-2.98331
-7.43122	-5.53309	-6.52554	-6.48835	-1.12817	-2.54618	-6.1205	-7.84751	-7.32034	-2.67816
-7.95425	-5.15083	-6.00751	-7.66965	-0.77286	-2.44496	-7.35033	-9.32389	-8.74135	-0.67437
-6.43644	-3.59077	-7.80068	-9.01555	-0.61043	-1.40247	-7.18771	-8.83176	-9.14735	-0.81677
-7.47224	-6.8452	-6.69489	-6.94344	-1.52409	-3.48746	-7.43164	-8.89202	-8.3049	-2.25096
-8.22089	-8.39493	-5.72857	-7.00154	-1.82864	-5.785	-7.42148	-7.7772	-7.51319	-3.74617
-9.24643	-7.65106	-6.33626	-6.61423	-1.19923	-4.75262	-5.90706	-6.87329	-7.23914	-3.42068
-7.92348	-6.67989	-6.05732	-7.88267	-1.49363	-4.48947	-6.36444	-7.66672	-7.71619	-2.85108
-7.71837	-6.52492	-6.0872	-7.82457	-1.47333	-4.54008	-7.04541	-8.06846	-7.26959	-2.39336
-7.6876	-7.12415	-6.29641	-6.68201	-1.66622	-3.11297	-7.15722	-7.91781	-8.11205	-3.57325
-7.70811	-8.05399	-4.68255	-6.96281	-1.13832	-4.39838	-6.97427	-7.53616	-7.70604	-2.51542
-8.11833	-7.60973	-5.82819	-7.83425	-1.8591	-4.71214	-7.9195	-8.37981	-7.60454	-4.0208
-7.21585	-6.74188	-6.64508	-6.93376	-0.91498	-3.00164	-5.88674	-7.11433	-6.96509	-1.95598
-7.8722	-8.00233	-5.92781	-7.34043	-2.77276	-5.03602	-8.19393	-9.27367	-7.73649	-4.24457
-8.20038	-8.3536	-5.92781	-7.43726	-1.66622	-6.22022	-7.25885	-8.10863	-7.84815	-3.37999
-8.67213	-8.39493	-5.21054	-7.28234	-1.72713	-5.68379	-7.24869	-8.27937	-7.68574	-3.27828
-8.6106	-7.32045	-6.03739	-8.10537	-1.8591	-5.05626	-8.46835	-9.09289	-8.1831	-2.71885
-7.91322	-7.5684	-6.31633	-7.76647	-1.89971	-3.63928	-7.97032	-8.57063	-8.49775	-3.25794
-7.74914	-7.91968	-5.29024	-6.74979	-2.28547	-5.57245	-7.61459	-7.90777	-7.66544	-4.74298
-7.99527	-7.27912	-5.75845	-7.97949	-1.23984	-3.8417	-7.08607	-8.15885	-7.39139	-3.28845
-8.67213	-8.72554	-5.19062	-7.75679	-1.33121	-1.97938	-7.1064	-8.35972	-7.82785	-3.25794
-7.45173	-6.50426	-7.05353	-8.13442	-1.8388	-3.21418	-8.02114	-9.02259	-8.23385	-2.16959
-8.02603	-7.33078	-6.26652	-7.96013	-1.23984	-4.61093	-7.72639	-8.01824	-7.39139	-2.1289
-7.76965	-7.41343	-5.08103	-6.87566	-1.8794	-4.99554	-7.07591	-8.24924	-7.71619	-4.10217
-7.85169	-6.03934	-7.6911	-8.23125	-1.38197	-3.12309	-6.2628	-7.42568	-7.78725	-2.18993
-8.20038	-6.40094	-5.56918	-6.55613	-1.16878	-2.91054	-7.54344	-8.53046	-8.45715	-2.04753
-7.75939	-7.62006	-5.93777	-6.8563	-1.90986	-4.67165	-7.39098	-8.49029	-7.77709	-3.5936
-8.0568	-6.4526	-6.99375	-8.23125	-2.1332	-6.4024	-8.70212	-9.36406	-8.244	-2.90193
-8.00552	-7.77504	-7.6911	-7.12741	-2.75245	-3.90243	-7.76705	-8.85185	-9.26915	-4.26492
-7.55428	-7.0415	-6.77459	-7.94076	-1.70682	-4.20607	-6.84214	-8.14881	-7.92935	-3.09519
-7.76965	-8.23995	-7.12326	-8.41522	-2.34638	-4.47935	-8.16344	-9.45446	-8.37595	-3.46137
-7.22611	-7.5994	-6.51557	-7.47599	-2.02153	-4.39838	-7.05558	-8.37981	-7.60454	-3.79703
-7.63633	-7.57874	-6.44584	-7.95045	-0.67134	-4.03401	-6.42542	-7.60646	-7.22899	-1.82375
-7.79016	-8.5189	-5.63891	-6.98217	-2.4479	-6.88822	-7.57393	-8.66102	-7.9192	-3.53257
-8.54118	-7.89086	-5.94078	-8.30193	-1.70623	-6.93433	-6.65858	-8.29308	-8.29689	-3.53638
-8.40977	-8.37896	-5.61352	-7.41322	-1.61938	-4.92893	-6.79323	-8.24326	-7.86239	-3.87232
-7.30222	-6.38971	-6.47259	-6.7068	-1.10793	-2.86513	-6.87979	-7.89454	-7.89136	-1.7415
-7.0488	-4.336	-8.24186	-8.05127	-0.51928	-1.27834	-7.62036	-8.63183	-8.51897	-0.49372
-8.17987	-5.18182	-6.42592	-7.96013	-0.448	-2.11096	-6.82181	-7.92785	-7.9192	-2.07804

-7.60556	-5.02685	-7.3225	-8.19252	-1.15863	-2.51581	-8.09229	-8.90207	-8.96465	-0.65403
-7.25687	-7.14481	-5.25039	-6.97249	-1.71697	-3.94292	-7.63492	-7.83746	-8.3049	-2.70868
-8.06706	-8.22962	-6.48569	-8.03759	-1.63576	-3.77086	-6.66935	-8.11868	-7.72634	-3.31897
-8.81337	-8.17635	-5.07149	-6.60426	-1.77378	-5.1431	-6.47584	-7.80487	-7.13822	-3.82433
-7.79016	-7.14481	-6.69489	-6.3528	-1.55455	-3.27491	-6.91328	-8.36976	-7.55379	-2.71885
-7.07227	-3.66309	-8.37848	-8.59919	-0.56982	-1.53404	-8.74278	-9.61515	-9.34021	-0.21666
-6.96972	-3.00187	-9.67356	-8.59919	-0.43785	-0.70409	-6.71001	-8.50033	-8.2846	-0.0946
-8.02494	-6.73046	-6.84076	-7.39043	-1.35883	-3.274	-6.40852	-7.61556	-7.65962	-3.4116
-7.22713	-5.0175	-6.401	-7.84618	-1.04038	-2.69964	-7.52418	-8.41264	-8.3838	-1.11761
-9.30145	-7.29224	-6.79985	-8.21078	-0.94388	-4.95814	-6.90864	-8.7215	-8.0555	-3.05646
-8.28775	-7.79877	-5.33739	-7.85757	-1.36848	-5.80508	-6.5624	-7.88458	-8.20034	-3.9683
-6.10081	-4.03208	-7.61801	-7.0714	-0.78948	-1.00577	-6.73552	-8.00414	-7.81411	-1.7319
-7.86195	-7.60973	-5.7983	-7.3598	-1.37181	-4.73238	-6.99459	-7.89772	-7.74664	-3.18674
-5.8929	-3.46679	-7.38228	-6.80788	-0.7221	-0.66361	-6.75066	-8.43002	-7.54364	-1.54912
-7.29283	-8.56315	-5.76692	-6.87771	-1.79308	-5.79534	-6.87017	-8.20341	-7.49547	-3.23883
-7.856	-6.07659	-5.93056	-7.50437	-1.09828	-3.83862	-6.64896	-7.68531	-7.76583	-2.16382
-6.8423	-4.12418	-6.61576	-6.78656	-0.44208	-1.08365	-7.101	-8.27315	-8.18103	-0.5897
-7.63073	-8.1211	-5.30671	-7.60691	-1.69658	-5.73693	-6.67781	-7.97425	-7.1189	-3.53638
-7.17483	-7.93001	-6.72478	-6.75947	-2.66109	-4.47935	-7.43164	-8.73133	-8.0207	-4.69212
-6.24158	-2.22701	-9.72337	-9.28666	-0.32618	-0.28912	-6.67952	-8.32959	-8.55865	-0.1658
-7.74914	-4.99586	-5.88796	-7.40821	-0.81347	-2.35387	-4.89068	-7.4759	-6.89404	-2.00684
-6.72028	-4.58465	-7.09643	-7.4474	-0.82808	-1.88191	-7.428	-8.27315	-7.9686	-1.40555
-9.23618	-5.46077	-7.05353	-7.3598	-0.78301	-2.61702	-8.39721	-8.06846	-7.63499	-1.18295
-8.43793	-6.22394	-5.98169	-7.2537	-0.93423	-3.81915	-6.69705	-7.68531	-7.52444	-2.01985
-7.15432	-4.89254	-7.18303	-7.29202	-0.37694	-1.3721	-7.41131	-8.20907	-8.68045	-1.83393
-8.03433	-7.13568	-5.9101	-8.26775	-1.05003	-4.48113	-6.71629	-7.93439	-7.56306	-3.30602
-7.67735	-4.63425	-6.54546	-7.8633	-0.98605	-1.88829	-8.11262	-8.39989	-9.056	-1.90513
-8.49778	-7.35144	-6.07724	-6.98217	-1.34136	-4.39838	-6.38476	-7.60646	-7.80755	-2.71885
-9.43103	-7.51675	-5.47952	-6.43994	-1.8794	-6.21009	-6.16116	-7.51607	-6.97524	-4.35646
-7.34917	-7.39277	-5.7983	-6.48835	-1.54439	-5.85585	-6.16116	-7.59642	-7.43199	-2.98331
-6.09801	-3.59077	-8.33864	-7.65996	-0.79316	-1.12919	-7.37066	-8.24924	-7.9598	-1.35586
-7.58505	-4.61359	-8.88655	-8.55078	-0.89468	-1.57453	-7.72639	-9.2335	-8.84285	-1.21346
-7.35854	-5.71742	-6.69758	-7.6183	-0.96318	-2.57309	-6.8317	-7.78494	-7.91067	-1.7223
-7.64012	-7.66983	-7.28052	-8.24496	-2.13083	-3.85809	-7.5915	-8.313	-8.41276	-5.04331
-6.55134	-3.13876	-6.59531	-6.91189	-0.68333	-0.74292	-7.19717	-7.83476	-7.86239	-0.72408
-7.57441	-6.23315	-6.20668	-7.66388	-0.73158	-2.62176	-6.49508	-7.39637	-7.56306	-2.2694
-7.35854	-4.94382	-6.98394	-7.76642	-1.03073	-2.2421	-6.96635	-8.28311	-8.11344	-1.78949
-8.02494	-7.5409	-4.8874	-7.04862	-1.78343	-5.44488	-6.43737	-7.43622	-7.14787	-3.9491
-8.47547	-7.90928	-5.81806	-8.38169	-1.60008	-4.83159	-7.12023	-7.92443	-7.64997	-3.57477
-6.54195	-5.04513	-7.43392	-7.35625	-1.10793	-2.53415	-6.93749	-7.69527	-8.00722	-1.7319
-8.03629	-6.75221	-6.9041	-7.32107	-1.36166	-3.61904	-6.75066	-8.45011	-7.838	-2.17976
-8.56957	-7.22746	-6.91406	-8.39585	-1.40227	-3.78098	-6.40509	-7.79729	-7.43199	-4.96675
-8.12859	-6.93818	-5.76842	-6.93376	-1.06726	-4.25668	-5.85624	-7.75711	-7.48274	-2.30181
-6.94921	-6.89686	-5.09099	-6.31406	-1.44288	-3.23443	-6.50673	-7.82742	-7.48274	-3.11554
-7.39019	-6.49393	-6.47573	-7.19519	-1.51394	-3.33564	-6.18149	-7.7069	-7.42184	-2.50524
-7.55428	-5.34713	-5.5891	-7.03059	-0.02163	-2.19193	-5.68346	-7.41564	-7.41169	-1.24398
-6.80563	-6.70056	-7.39224	-6.80788	-1.04696	-2.8903	-6.23231	-7.71694	-7.36094	-2.92228
-8.52855	-7.35144	-6.30637	-9.41254	-0.87438	-6.16961	-5.11428	-7.3152	-7.43199	-3.39017

-8.49778	-6.091	-7.65125	-7.59219	-1.59515	-3.3154	-7.39098	-8.65098	-8.041	-2.67816
-8.40549	-8.13664	-5.85808	-6.78852	-1.57485	-4.34777	-6.20181	-8.12872	-7.9801	-3.48171
-7.06202	-4.58259	-6.5355	-7.26297	-0.59013	-1.71623	-5.88674	-8.14881	-7.76694	-0.83712
-6.86717	-5.04751	-10.859	-10.6519	-1.11802	-2.76884	-8.62081	-9.73567	-10.071	-0.97952
-7.41485	-6.12264	-5.25557	-6.5245	-0.99213	-3.22533	-6.81246	-7.74509	-7.621	-2.06784
-7.23636	-8.27095	-5.82819	-7.36948	-1.65606	-4.39838	-6.72017	-7.84751	-8.17295	-2.50524
-8.73366	-7.15514	-6.10713	-8.31839	-1.30075	-5.06638	-6.38476	-7.84751	-7.56394	-2.87142
-7.97476	-6.54558	-5.98758	-6.7401	-1.17893	-3.15345	-6.151	-7.51607	-7.72634	-2.60696
-7.39019	-7.83702	-6.97383	-9.08333	-1.43272	-5.51172	-7.99065	-8.97237	-8.5282	-2.60696
-7.33892	-4.62392	-6.3761	-7.68901	-0.65104	-1.97938	-6.89296	-7.94794	-7.63499	-0.99986
-8.15935	-5.58475	-6.25656	-8.11505	-1.18908	-3.16358	-7.81787	-9.93654	-9.41126	-2.08821
-7.06202	-3.50812	-9.64367	-9.19952	-0.81347	-0.97737	-8.5395	-8.89202	-10.1319	-1.01003
-8.66187	-7.54774	-5.83815	-8.39585	-1.65606	-5.98743	-7.4418	-7.83746	-7.59439	-3.55291
-8.0531	-6.56469	-6.34986	-7.88036	-1.03073	-3.29347	-7.62036	-8.22333	-8.00722	-2.35579
-8.94903	-7.77504	-6.22667	-7.63092	-2.08244	-4.87408	-8.23458	-8.51037	-8.4876	-3.00365
-9.25669	-8.16763	-5.52933	-7.3017	-2.51896	-5.87609	-7.43164	-8.37981	-8.90375	-4.83452
-9.22592	-7.31012	-4.81206	-6.99186	-1.08756	-5.14736	-6.76083	-7.52612	-7.46244	-3.69531
-8.27216	-6.43194	-5.13084	-6.47867	-1.49363	-4.35789	-7.12672	-7.37546	-7.06659	-2.6273
-8.47727	-5.06818	-7.33247	-8.53141	-0.66119	-2.66763	-7.60443	-8.76146	-8.25415	-1.04055
-8.43625	-7.36177	-5.83815	-6.62391	-1.76773	-4.7425	-7.46213	-7.71694	-7.59439	-3.39017
-6.88768	-6.30796	-5.2205	-6.95312	-0.85407	-2.64739	-6.28313	-7.86759	-7.05644	-2.59679
-7.12388	-7.17252	-6.81008	-7.93733	-1.92818	-3.09877	-7.2645	-8.08384	-7.99757	-3.73794
-8.6106	-7.58907	-8.39841	-8.91872	-1.95046	-3.7911	-7.36049	-8.65098	-8.19325	-3.23759
-6.69282	-5.56409	-6.12705	-8.15378	-1.36166	-2.12108	-8.12278	-9.09289	-8.3252	-1.81358
-7.72862	-8.04365	-5.39982	-7.85362	-1.79819	-5.13723	-6.93361	-7.65668	-8.41655	-3.31897
-8.93877	-7.31012	-6.10713	-7.80521	-1.34136	-4.10486	-7.19787	-8.55055	-8.3252	-3.67497
-8.30293	-6.97951	-6.63512	-8.62824	-1.74743	-3.66964	-8.50901	-8.7012	-8.76165	-2.35267
-8.35421	-8.21929	-6.92402	-8.11505	-2.11289	-4.17571	-7.96016	-9.44441	-8.3252	-3.46137
-8.50804	-8.41559	-5.83815	-8.43458	-1.95046	-5.80524	-7.8077	-9.26363	-8.2237	-3.75634
-7.9645	-7.97133	-5.85808	-7.35012	-1.95046	-4.75262	-7.49262	-8.41998	-7.08689	-3.39017
-7.08253	-6.13232	-6.40599	-8.39585	-1.86925	-2.36399	-8.43786	-8.40994	-9.06615	-2.64765
-8.49778	-8.28128	-5.68872	-7.06932	-1.8794	-4.83359	-7.36049	-8.34968	-7.92935	-3.64445
-8.68238	-7.2378	-5.28027	-8.29903	-1.93016	-4.95505	-7.6959	-7.52612	-8.76165	-2.71885
-9.16439	-8.11598	-6.05732	-8.56046	-1.30075	-5.1676	-6.99459	-8.05842	-8.041	-2.33233
-8.18451	-7.31987	-5.63397	-7.90315	-1.32023	-3.4687	-7.16832	-7.97425	-7.73687	-3.3924
-7.56454	-6.75221	-6.4558	-7.00154	-1.41242	-4.27692	-6.8523	-7.97807	-7.78725	-1.80341
-8.47547	-6.54627	-6.8203	-7.83479	-1.47463	-3.48817	-7.18756	-8.08384	-7.74652	-2.67253
-8.28775	-7.80798	-5.65443	-6.99165	-1.86063	-5.46435	-6.38928	-7.61556	-7.08028	-4.49621
-8.55056	-8.39738	-6.71803	-8.24496	-2.07293	-6.7591	-7.01444	-8.39271	-7.95895	-3.68035
-8.25021	-7.57774	-5.49079	-7.54994	-0.93423	-4.80238	-6.86055	-7.92443	-7.95895	-2.24061
-7.79968	-7.02517	-5.22489	-7.812	-1.27198	-4.42272	-6.89902	-8.03403	-7.73687	-2.17342
-8.37223	-7.36592	-6.49304	-7.39043	-1.66763	-4.1112	-6.96635	-8.40267	-7.8817	-3.88192
-8.30653	-7.65141	-6.05328	-8.31332	-1.71588	-5.52276	-6.74514	-8.2333	-7.57272	-3.03727
-7.69643	-6.23315	-6.68735	-7.84618	-0.94388	-2.75805	-7.46647	-8.65176	-7.9686	-2.45177
-8.54118	-7.71588	-6.94303	-8.83744	-1.47463	-5.26966	-7.60112	-9.10011	-8.26793	-2.20221
-7.46178	-4.69517	-7.98618	-6.94607	-0.86668	-2.02793	-7.76462	-8.16355	-8.03619	-1.36716
-7.9217	-7.1541	-6.26805	-8.49562	-1.86063	-3.85809	-7.9666	-8.63183	-8.17137	-3.09486
-8.28775	-7.08963	-6.14532	-8.08545	-1.53253	-4.2767	-7.79348	-8.76136	-8.44173	-2.22141

-6.80476	-5.23853	-6.8203	-8.05127	-1.20443	-1.71641	-7.65883	-8.52224	-8.28724	-1.79908
-8.96355	-8.1211	-5.84874	-8.3475	-2.25628	-5.91216	-7.84156	-8.48238	-7.82377	-3.44039
-8.33468	-8.1211	-5.71579	-7.88036	-1.86063	-5.54223	-7.00482	-8.25322	-7.65962	-3.51718
-8.56933	-8.14873	-5.17376	-6.92329	-1.47463	-5.68826	-6.7259	-7.79491	-7.50513	-3.54598
-7.97801	-6.62916	-8.09868	-8.11963	-1.84133	-3.64393	-8.38978	-8.39271	-8.67346	-2.64374
-7.26468	-5.07276	-6.30895	-6.8891	-1.22373	-2.89434	-8.06277	-8.76136	-7.90101	-1.1368
-7.48055	-6.38971	-6.32941	-7.20813	-1.32023	-3.75101	-7.94736	-8.03403	-7.68859	-2.31739
-8.03433	-6.67521	-6.11464	-7.51576	-1.21408	-3.85809	-7.01444	-8.09381	-7.86239	-2.14462
-7.69643	-7.68825	-6.05328	-7.2537	-2.08258	-4.32537	-8.22628	-9.01044	-8.18103	-3.54598
-7.66827	-7.91849	-6.176	-7.51576	-1.98608	-4.90946	-7.39915	-9.41894	-7.94929	-2.73012
-8.2596	-7.10805	-5.89987	-8.29054	-1.85098	-4.56874	-8.00507	-8.55213	-8.02654	-3.08526
-7.43362	-6.68442	-6.81008	-8.5298	-2.07293	-3.78995	-8.27436	-9.10011	-8.44173	-2.90289
-8.26898	-8.64604	-5.82829	-7.78921	-1.82203	-5.87322	-6.95673	-8.40267	-8.03619	-3.54598
-7.80907	-6.72125	-5.02035	-5.92064	-1.41673	-3.6634	-6.7259	-7.36648	-7.09959	-2.8645
-7.46178	-5.20169	-7.46461	-8.98555	-1.45533	-2.90407	-8.34169	-9.13997	-8.9921	-1.53033
-7.62134	-6.5739	-6.52372	-8.15381	-1.68693	-3.26426	-7.38953	-8.19344	-7.82377	-3.07566
-8.4567	-7.91849	-5.83851	-8.03987	-2.12118	-5.62985	-7.50494	-8.71154	-8.35483	-3.81473
-8.09065	-7.93691	-5.81806	-8.56399	-1.47463	-5.81481	-7.63959	-9.46876	-8.24862	-1.6935
-7.78091	-7.06201	-5.83851	-7.37904	-1.89923	-4.5298	-7.48571	-8.62187	-7.77549	-2.92209
-8.36284	-4.44651	-8.51799	-7.37904	-0.20083	-1.75535	-8.84181	-9.25953	-9.23349	-0.45532
-8.36284	-6.43576	-5.62375	-6.66123	-1.05003	-4.082	-7.2645	-8.58202	-8.11344	-2.05824
-8.41916	-7.75272	-6.73849	-8.14242	-2.35278	-4.64662	-8.12048	-8.7215	-8.50932	-3.25803
-6.72028	-5.2109	-6.92257	-7.84618	-1.55183	-2.09608	-8.30322	-9.1599	-8.43207	-1.90467
-7.7799	-6.87619	-5.65883	-7.45663	-1.35151	-3.56843	-7.1064	-8.0785	-8.4876	-2.67816
-8.2414	-9.3041	-6.27648	-6.56582	-2.69154	-6.12912	-7.27918	-8.55055	-8.1222	-5.60755
-8.50804	-5.53309	-6.49565	-8.483	-0.83377	-3.06236	-6.47624	-7.51607	-7.94965	-1.76272
-7.22611	-5.67774	-6.10713	-6.78852	-0.60028	-2.48545	-5.54116	-7.55625	-7.65529	-1.66101
-7.90297	-7.02083	-6.07724	-7.63092	-1.44288	-3.68988	-7.50279	-8.09859	-8.61955	-2.81039
-8.15935	-5.96702	-6.79451	-7.28234	-0.97589	-3.20406	-6.8523	-7.74707	-7.67559	-2.83073
-7.09279	-5.88437	-6.94394	-8.02791	-0.7424	-3.05224	-6.95394	-7.92785	-7.69589	-0.81677
-7.36968	-6.62824	-5.83815	-7.38885	-0.71195	-3.30527	-6.30345	-7.64664	-7.9598	-1.80341
-7.97476	-7.11382	-6.20675	-7.90203	-1.49363	-3.68988	-6.80148	-7.92785	-7.51319	-2.37302
-7.81067	-6.21498	-6.2466	-7.70838	-1.16878	-2.90042	-6.76083	-7.35538	-8.15265	-2.10856
-7.51326	-6.40094	-7.31254	-7.40821	-1.39212	-3.24455	-6.38476	-7.65668	-7.43199	-2.01701
-7.31841	-7.25846	-4.80209	-6.37216	-1.2703	-6.32143	-5.68346	-7.40559	-7.55379	-3.22742
-7.10304	-5.3368	-4.87183	-6.49804	-0.90483	-2.62715	-5.6428	-7.39555	-7.31019	-1.67118
-8.07731	-7.73371	-5.54925	-6.91439	-1.92001	-4.85384	-8.13295	-8.63089	-8.3861	-4.3768
-7.80041	-5.90503	-6.17686	-8.22156	-1.40227	-3.2243	-8.39721	-9.15315	-8.59925	-1.62032
-6.60052	-5.02685	-7.17307	-8.97682	-1.55455	-1.80732	-8.43786	-9.15315	-9.42141	-1.76272
-9.16439	-6.2563	-5.76842	-6.62391	-1.38197	-3.43685	-7.97032	-9.38415	-8.2237	-3.06468
-8.92851	-8.58089	-5.38986	-7.22424	-2.00122	-5.91658	-8.2854	-8.41998	-7.8989	-4.5192
-8.16961	-7.51675	-6.19679	-7.04995	-1.49363	-4.5502	-7.89918	-9.27367	-9.16765	-2.07804
-7.856	-4.79647	-6.11464	-7.03722	0.146565	-2.20316	-6.36043	-8.22333	-7.75618	-0.88725
-7.54626	-5.2109	-8.00664	-8.82604	-1.05003	-2.03767	-7.39915	-8.30304	-8.43207	-1.78949
-8.1491	-6.56625	-6.65504	-8.67665	-1.09772	-3.65952	-7.87885	-8.56059	-9.04585	-1.33552
-8.50804	-7.39277	-5.60902	-7.28234	-1.30075	-3.59879	-7.35033	-8.26933	-7.80755	-3.19691
-7.63633	-5.01652	-7.26273	-8.1441	-0.49876	-1.72635	-7.08607	-8.20907	-8.09175	-1.53895
-6.92678	-3.78343	-8.56912	-7.45879	-0.87633	-1.47304	-7.85118	-9.06026	-8.49966	-0.91604

-9.41052	-8.61189	-6.63512	-7.35012	-1.36166	-6.14937	-7.5841	-8.89202	-7.838	-4.0208
-7.0488	-5.57928	-5.78738	-7.41322	-0.63508	-2.26157	-6.2354	-7.46611	-7.2058	-2.279
-6.48771	-4.53094	-7.50182	-8.16347	-1.2906	-1.47332	-7.19787	-8.14881	-8.2034	-1.17278
-8.00552	-8.12631	-5.68872	-11.0973	-1.41242	-4.66153	-7.93983	-8.95228	-8.29475	-1.80341
-6.85691	-6.091	-6.42592	-7.61155	-1.19923	-3.06236	-8.0618	-8.83176	-8.17295	-1.14226
-7.30815	-5.6984	-6.04736	-7.49536	-0.64089	-2.64739	-7.61459	-8.28942	-7.72634	-1.67118
-7.12355	-8.07465	-6.43588	-6.92408	-2.41745	-3.83158	-6.72017	-8.51037	-8.3252	-4.12251
-7.09279	-5.41945	-7.35239	-8.04727	-1.48348	-2.09072	-7.11656	-7.9379	-8.17295	-2.47473
-7.06757	-6.58311	-5.41921	-7.65249	-0.39383	-4.10147	-6.05266	-8.09381	-7.22512	-1.93346
-7.36968	-7.47542	-5.51936	-7.45663	-2.02153	-6.75665	-7.42148	-8.08855	-8.55865	-4.17337
-7.021	-5.8947	-6.19679	-6.43994	-1.23984	-2.61702	-6.65919	-7.34533	-7.13764	-2.06787
-7.90297	-5.92569	-4.82202	-6.00422	-0.80331	-3.05224	-6.84214	-6.84316	-7.24929	-1.61015
-7.73888	-7.27912	-5.2205	-6.60455	-1.2906	-5.56233	-7.15722	-7.56629	-7.34064	-2.44422
-7.63073	-7.53169	-5.38852	-7.8006	-2.35278	-6.63254	-6.54317	-8.02406	-7.39892	-4.16027
-8.60034	-7.57874	-5.67876	-6.05263	-1.75758	-3.27491	-6.77099	-8.02829	-7.37109	-2.99348
-6.53256	-5.51481	-7.48506	-7.69806	-1.12723	-2.09608	-7.82233	-9.30935	-8.12309	-1.86627
-6.17589	-3.70975	-7.11689	-7.2537	-0.22013	-0.57743	-7.67806	-8.29308	-8.49001	-0.88725
-8.45676	-7.0105	-6.75466	-8.24093	-1.26014	-3.11297	-7.59426	-8.2392	-7.84815	-2.28147
-7.83722	-8.24082	-6.34986	-7.13977	-1.60973	-4.26696	-6.75476	-8.10377	-7.33133	-4.43862
-6.90819	-7.09315	-6.1669	-6.87566	-1.04696	-3.02188	-6.52706	-7.21477	-6.60984	-2.54593
-7.47224	-6.88652	-5.39982	-8.39585	-1.73728	-4.73238	-7.40115	-8.05842	-7.59439	-3.91908
-8.30653	-6.55548	-5.97146	-7.77782	-1.78343	-4.34484	-7.50494	-8.8311	-8.42242	-3.24843
-8.46702	-8.67388	-5.15077	-6.2947	-1.70682	-5.62306	-7.65524	-8.20907	-8.0004	-3.43085
-7.82092	-7.15514	-5.70864	-6.52708	-1.5647	-4.34777	-6.3746	-7.63659	-7.43199	-3.11554
-9.07209	-7.44443	-5.92781	-8.29903	-1.66622	-5.68379	-7.46213	-8.67107	-8.03085	-3.28845
-7.503	-7.5684	-5.96766	-7.97949	-1.2906	-3.51782	-6.95394	-7.90777	-7.838	-2.37302
-7.39019	-5.92569	-6.69489	-7.32107	-1.20939	-3.12309	-6.25263	-7.99816	-7.74664	-2.26113
-9.05158	-7.14481	-5.34005	-7.37917	-2.07229	-5.67366	-6.76083	-7.76716	-7.44214	-3.57325
-8.07731	-7.09315	-5.24043	-6.38184	-1.19923	-4.4085	-6.60837	-7.87764	-7.96995	-2.4849
-8.43625	-6.04967	-6.12705	-7.48568	-0.91498	-2.698	-7.4723	-8.10863	-7.70604	-2.72902
-7.94047	-6.59232	-6.32941	-8.11963	-1.13688	-3.36161	-6.73552	-8.24326	-8.07481	-2.06784
-8.0531	-6.40813	-5.43966	-6.38778	-1.01143	-3.18639	-6.5624	-8.10377	-7.75618	-2.74932
-6.6518	-2.83657	-9.62375	-9.14142	-0.79316	-1.07858	-7.74672	-10.0771	-9.1981	-0.7354
-7.84143	-6.86586	-5.82819	-6.75947	-1.72713	-4.27692	-6.93361	-7.66672	-7.88875	-2.6273
-7.28345	-7.75272	-6.14532	-7.96012	-2.45893	-4.84132	-7.62997	-8.63183	-8.33552	-3.68995
-8.94903	-8.93217	-7.22288	-8.89936	-2.38699	-7.05016	-8.20409	-9.45446	-8.9342	-1.7729
-8.11833	-6.57658	-6.13701	-6.91439	-0.39724	-3.28503	-5.62247	-7.36542	-7.06659	-2.61713
-8.2414	-7.38244	-5.74849	-7.48568	-1.82864	-5.95706	-7.29951	-8.14881	-8.3252	-3.53257
-7.97476	-4.86155	-6.07724	-7.3017	-0.35664	-2.31339	-6.95394	-8.7012	-8.65	-0.84729
-7.40045	-7.71305	-6.47573	-8.26998	-2.30578	-4.41862	-6.7405	-8.43002	-7.61469	-4.39715
-7.53377	-5.8637	-9.11568	-9.39317	-1.57485	-3.59879	-8.15327	-9.60511	-9.90861	-1.15243
-8.65162	-7.97133	-5.47952	-6.54645	-2.54942	-8.00157	-7.4723	-8.37981	-8.43685	-4.1632
-7.86195	-8.04365	-5.70864	-7.9214	-2.1738	-4.96517	-7.42148	-8.48024	-8.1831	-3.27828
-7.73888	-8.26062	-6.22667	-7.36948	-1.67637	-5.14736	-6.99459	-8.45011	-8.07145	-3.32914
-7.22611	-7.36177	-6.20675	-8.38617	-2.64078	-5.41051	-7.81787	-8.89202	-8.76165	-4.41749
-7.49933	-6.55548	-6.60554	-8.79186	-1.98608	-4.10147	-7.72615	-8.97059	-8.43207	-2.47097
-8.99005	-6.14265	-6.02743	-7.84394	-1.3109	-3.65952	-7.43164	-8.32959	-8.1831	-2.36284
-7.83118	-7.0105	-6.40599	-7.67933	-2.06213	-3.32552	-7.55361	-8.68111	-8.2643	-2.90193

-6.32363	-4.06602	-9.10572	-8.89936	-1.14847	-1.44295	-7.12672	-9.26363	-8.9951	-0.29803
-7.37994	-7.41343	-5.3002	-8.44427	-2.26517	-3.8417	-7.45197	-8.39989	-8.35565	-3.79703
-8.19012	-7.71305	-5.64887	-7.81489	-1.46318	-4.58056	-7.37066	-8.25929	-7.76694	-3.34948
-7.98501	-6.55592	-7.66121	-7.26297	-1.41242	-2.78909	-7.30967	-8.61081	-8.13235	-2.60696
-7.86538	-6.09501	-6.06351	-7.812	-1.32023	-3.58552	-6.3989	-7.97425	-7.4472	-3.14285
-8.0568	-5.04751	-6.97383	-7.29202	-0.35664	-3.16358	-6.57788	-8.10863	-8.27445	-0.99986
-8.03629	-6.87619	-5.81823	-7.4179	-1.30075	-3.7911	-6.54739	-7.84751	-7.66544	-2.14924
-8.86698	-7.00017	-6.12705	-7.38885	-1.55455	-4.6919	-7.43164	-8.03833	-7.21884	-3.32914
-6.1903	-2.87789	-7.52175	-7.90203	-0.64089	-0.98749	-6.86246	-8.7715	-8.53835	-0.43026
-7.18508	-4.02469	-8.97621	-8.49268	-0.57998	-1.14943	-8.24475	-8.85185	-9.77666	0.169861
-8.01578	-5.64674	-5.84811	-6.26565	-1.12817	-2.65751	-7.35033	-8.28942	-8.3455	-0.99986
-7.62134	-6.8594	-8.05777	-9.19064	-1.68693	-3.94571	-8.68793	-9.0503	-9.09831	-1.29037
-7.84661	-6.10422	-6.18623	-7.47019	-1.72553	-3.84836	-6.96635	-8.04399	-7.76583	-2.8165
-8.12819	-7.26461	-6.73849	-8.47284	-1.75448	-4.62715	-7.17794	-8.61191	-8.25827	-2.42297
-7.46178	-7.21857	-6.05328	-6.36499	-1.58078	-3.39082	-7.19717	-8.55213	-8.07481	-3.49798
-7.84661	-6.46339	-5.63397	-7.29928	-1.26233	-3.14745	-7.05291	-8.25322	-8.10378	-2.74932
-8.04372	-7.01596	-7.07598	-7.59552	-1.67728	-3.40055	-7.74539	-8.47242	-8.33552	-2.50936
-7.56503	-4.77805	-8.07823	-8.24496	-0.84738	-2.60229	-7.91851	-9.03037	-8.72174	-0.79126
-8.10942	-6.94228	-6.31918	-8.32472	-1.87993	-4.33511	-7.27412	-8.52224	-8.0555	-2.45177
-8.23144	-7.14489	-7.38279	-8.3475	-1.53253	-4.16961	-7.38953	-8.22333	-7.87205	-2.70133
-7.67766	-5.10038	-8.55889	-8.32472	-1.32988	-2.72884	-8.61099	-9.26949	-9.33971	-0.96403
-8.0531	-6.7673	-5.6442	-7.4474	-1.26233	-4.28643	-6.41813	-7.84472	-7.41823	-3.07566
-8.53179	-6.72125	-6.1351	-7.00304	-1.30093	-3.76075	-7.29335	-8.11373	-7.7079	-2.52856
-6.87985	-2.86248	-6.38054	-6.58147	-0.85703	-1.26861	-8.33207	-8.71154	-8.72174	-0.20577
-7.64658	-5.57442	-6.54546	-7.23392	-1.12817	-2.99151	-7.51295	-8.2392	-7.9192	-1.95598
-9.07209	-6.72122	-6.60523	-7.6987	-1.46318	-3.26479	-6.0087	-7.58638	-7.66544	-2.79005
-7.73888	-5.57442	-6.52554	-7.43726	-1.0165	-1.83768	-6.67952	-8.17894	-7.9598	-2.38319
-7.56454	-5.64674	-6.09717	-7.06932	-0.21451	-2.23241	-5.93755	-7.80733	-7.69589	-0.92866
-5.4314	-4.31398	-8.15932	-8.40554	-1.08756	-1.95914	-7.2182	-8.0785	-8.2034	-1.41689
-8.26898	-6.56469	-6.0226	-8.18799	-0.96318	-3.82889	-6.33157	-7.58567	-7.74652	-2.61494
-7.93373	-6.98984	-6.1669	-7.3017	-1.40227	-3.65952	-6.61853	-8.0785	-7.73649	-2.40353
-8.36446	-6.44227	-7.98996	-8.58951	-1.07741	-3.29515	-7.23853	-8.49029	-8.7718	-1.66101
-9.70793	-6.67989	-8.41833	-8.45395	-1.22969	-3.6494	-7.67557	-9.57498	-9.93906	-1.11175
-8.03629	-6.091	-8.17924	-7.70838	-1.10787	-3.01176	-7.90934	-9.15315	-9.259	-1.7729
-8.50804	-5.61575	-7.193	-8.23125	-1.16878	-2.54618	-7.65524	-8.27937	-7.8786	-1.96616
-8.0568	-7.05183	-4.55304	-6.46899	-2.08244	-6.52386	-6.18149	-7.83746	-7.30004	-4.48869
-7.63073	-6.00291	-5.6442	-7.13977	-0.71228	-3.03063	-5.92763	-7.48604	-7.37961	-2.52856
-7.80041	-7.33078	-6.73474	-7.24361	-1.99107	-4.13522	-6.84214	-8.18898	-7.77709	-3.13588
-7.23636	-5.75006	-5.84811	-7.67933	-0.83377	-3.01176	-6.46607	-7.88768	-7.838	-1.65084
-8.1491	-6.18398	-5.46955	-6.87566	-1.79819	-4.32753	-7.13689	-7.68681	-7.66544	-3.54274
-8.11833	-7.5684	-5.23046	-6.55613	-1.54439	-4.38826	-7.27918	-8.3095	-7.33049	-3.28845
-7.85169	-8.01266	-5.54925	-7.67933	-1.09772	-4.57044	-6.5982	-8.01824	-8.03085	-2.82056
-6.48771	-4.9132	-6.70485	-6.86598	-0.86423	-1.60489	-6.61853	-7.83746	-7.30004	-1.31518
-8.15935	-6.47326	-5.89792	-8.00854	-1.08756	-3.99352	-7.03525	-8.06846	-8.2643	-1.9153
-7.97476	-7.00017	-5.98758	-6.93376	-1.55455	-4.41862	-6.53722	-8.29946	-7.9801	-2.87142
-8.11833	-7.54774	-5.56918	-6.8563	-1.62561	-5.89633	-7.28935	-8.13877	-7.67559	-2.77988
-6.44669	-3.9937	-6.81444	-7.55345	-0.4277	-1.52392	-7.4418	-9.25359	-9.00525	-0.25734
-8.28242	-6.39061	-6.22667	-7.62123	-0.31603	-3.65952	-5.95788	-7.51607	-7.16809	-1.93564

-7.17483	-5.31613	-6.09717	-6.82725	-0.86423	-2.49557	-7.04541	-7.99816	-8.05115	-1.7729
-8.39523	-6.72122	-6.9041	-7.45663	-1.71697	-3.74049	-7.5841	-8.61081	-9.31991	-2.95279
-8.10808	-7.39277	-6.27648	-8.27966	-1.48348	-5.785	-7.68574	-8.9322	-8.3455	-2.33233
-6.74844	-4.21627	-8.65094	-7.58412	-1.33953	-1.71641	-7.8608	-8.62187	-8.8183	-0.84885
-7.80907	-7.50406	-6.20668	-7.53855	-1.24303	-5.5617	-6.13922	-7.77498	-7.34098	-2.77811
-7.7152	-5.38588	-5.98169	-6.50172	-0.95353	-2.70938	-6.22578	-7.46611	-7.24443	-2.44217
-7.89271	-7.0105	-5.19062	-6.44962	-1.70682	-7.33356	-7.04541	-8.33963	-7.54364	-3.4512
-7.93108	-5.88319	-6.45213	-7.36764	-0.73158	-3.05983	-6.98558	-7.96428	-8.23896	-1.42475
-7.92348	-8.36393	-5.68872	-6.86598	-1.96062	-5.65342	-7.56377	-8.38985	-7.9801	-3.51222
-7.36968	-6.8142	-5.5891	-7.42758	-1.12817	-3.76073	-7.4418	-9.02259	-8.07145	-2.71885
-7.48249	-5.63641	-6.66501	-8.32807	-0.49876	-2.75872	-6.63886	-7.60646	-7.33049	-1.13209
-8.44732	-7.6422	-5.89987	-7.37904	-1.86063	-3.48817	-7.57227	-7.81483	-8.4707	-3.23883
-8.35421	-7.38244	-5.67876	-6.65296	-2.03168	-3.97328	-7.31984	-8.59072	-7.92935	-3.78685
-6.91739	-6.67521	-6.79985	-7.812	-1.46498	-3.99438	-6.68743	-7.99417	-7.8817	-2.73012
-7.46178	-6.75809	-5.16353	-5.74973	-1.09828	-3.93597	-5.60062	-7.04765	-6.54922	-2.77811
-8.53881	-7.32045	-5.09099	-6.32375	-1.43272	-3.58867	-7.56377	-8.15885	-8.25415	-2.57645
-8.07731	-6.44227	-5.48948	-6.70137	-0.87438	-3.80122	-6.6287	-7.16455	-7.8989	-3.54274
-8.66187	-7.07249	-6.39603	-8.25061	-1.6053	-4.90444	-8.16344	-8.51037	-8.1222	-1.48809
-7.06202	-4.82022	-7.4022	-7.99886	-0.80331	-2.15144	-8.20409	-8.58068	-9.47216	-0.55232
-8.04654	-6.43194	-6.72478	-8.56046	-1.26014	-3.30527	-7.57393	-7.62655	-7.82785	-2.83073
-7.79016	-6.38028	-6.81444	-8.86062	-1.59515	-3.54819	-7.83819	-8.35972	-7.63499	-2.22044
-8.79519	-7.82669	-6.0872	-7.83425	-1.36166	-5.17772	-7.64508	-7.73703	-7.838	-2.29164
-6.71333	-4.89254	-8.14936	-7.42758	-0.90483	-1.96926	-7.35033	-9.02259	-7.8989	-0.72523
-7.91322	-7.42376	-6.02743	-7.85362	-1.66622	-4.80323	-6.61853	-8.84181	-8.1019	-2.6273
-8.48753	-7.2068	-6.58531	-7.94076	-1.59515	-3.9328	-7.63492	-8.27937	-8.66015	-2.66799
-7.62607	-6.48359	-6.20675	-8.46363	-1.23984	-2.32351	-7.54344	-7.83746	-7.88875	-3.50205
-7.99527	-7.12415	-5.86804	-8.35712	-1.81849	-4.65141	-8.99687	-8.60076	-8.23385	-3.25794
-7.79016	-6.76255	-5.93777	-7.74711	-1.33121	-4.11498	-7.45197	-7.65668	-7.838	-2.25096
-7.56454	-7.50642	-5.27031	-7.27266	-2.18396	-5.89633	-7.59426	-7.72698	-8.43685	-3.43085
-6.68257	-4.9132	-7.76084	-8.62824	-1.75758	-1.74659	-8.51917	-9.25359	-8.0004	-2.45439
-7.57479	-6.04967	-6.12705	-7.80521	-1.40227	-3.8417	-8.32606	-7.98811	-8.4267	-2.59679
-8.86698	-8.59123	-6.09717	-7.0209	-2.48851	-6.28094	-8.23458	-8.17894	-7.92935	-2.04753
-7.66709	-7.10349	-5.03122	-7.04027	-1.62561	-3.58867	-8.11262	-7.81738	-7.58424	-2.70868
-7.65684	-5.3058	-6.20675	-7.37917	-0.97589	-2.62715	-7.43164	-8.33963	-8.8124	-1.8441
-7.60556	-7.48575	-5.35997	-6.73042	-1.46318	-4.52995	-6.87263	-7.79729	-7.69589	-1.56947
-7.89271	-6.02901	-7.16311	-8.23125	-1.8794	-2.98139	-7.50279	-8.38985	-8.31505	-2.10856
-7.46198	-5.93602	-6.41595	-8.95745	-1.73728	-3.75061	-7.27918	-8.40994	-8.8124	-2.26113
-8.54906	-6.91752	-4.54308	-6.62391	-1.8388	-4.04413	-8.25491	-8.13877	-8.19325	-3.04433
-7.6876	-6.22531	-7.30258	-8.47331	-1.70682	-3.39637	-8.4277	-8.11868	-8.45715	-2.4849
-8.34395	-8.47758	-6.0872	-8.37649	-1.47333	-5.29918	-6.98443	-8.78155	-8.0207	-2.58662
-8.71315	-8.45692	-5.81823	-7.68901	-2.04183	-5.13723	-7.67557	-8.64094	-7.92935	-3.68514
-7.25687	-7.25846	-5.54925	-6.95312	-0.81347	-4.78299	-6.77099	-7.13442	-6.09218	-1.9153
-8.89775	-7.41343	-6.55542	-8.13442	-2.01138	-4.81335	-7.60443	-8.81168	-8.39625	-3.22742
-7.43122	-6.01868	-5.68872	-7.45663	-1.33121	-3.91255	-6.72017	-7.65668	-8.17295	-2.31199
-8.55932	-8.73587	-4.73236	-6.45931	-2.16365	-7.1615	-7.15722	-9.20337	-8.13235	-4.15303
-7.67735	-7.41343	-5.90789	-7.32107	-1.23984	-1.9895	-8.20409	-8.39989	-8.25415	-3.54274
-0.7344	-0.68761	-0.98661	-1.1919	-1.19923	-0.72434	-0.45924	-0.7468	-0.7228	0.56655
-7.65684	-7.39277	-6.22667	-7.89235	-1.22969	-3.60891	-7.36049	-8.53046	-7.78725	-2.11873

-7.05176	-6.41127	-8.58769	-9.62556	-1.75758	-2.42472	-8.98671	-10.037	-9.0966	-1.99667
-7.30815	-7.15514	-6.46576	-9.30603	-1.80834	-3.18382	-7.62475	-8.03833	-8.09175	-2.70868
-7.79016	-8.22962	-6.40599	-8.26029	-1.48348	-4.21619	-8.21426	-9.08285	-8.8124	-1.94581
-8.04654	-7.88868	-5.9577	-7.35012	-1.39212	-4.05425	-6.73033	-7.99816	-8.05115	-2.39336
-7.99527	-7.49608	-7.07345	-8.28934	-1.88955	-4.2668	-7.68574	-8.27937	-8.19325	-2.01701
-7.85169	-7.29979	-6.1669	-7.79552	-1.09772	-4.31741	-6.63886	-8.01824	-8.01055	-2.6985
-7.99527	-7.7647	-6.96387	-8.41522	-2.05198	-4.71214	-6.80148	-7.91781	-7.84815	-3.96994
-7.58505	-8.27095	-5.68872	-6.82725	-1.22969	-4.85384	-7.71623	-7.74707	-7.50304	-3.06468
-8.99005	-7.14481	-6.44584	-8.35712	-1.35151	-3.04212	-7.62475	-8.08855	-8.3252	-3.03416
-8.44651	-6.56625	-5.41974	-7.40821	-1.72713	-3.95304	-7.22836	-7.71694	-7.66544	-2.65782
-7.52352	-7.37211	-5.9577	-6.41089	-1.61546	-3.67976	-7.1064	-8.35972	-7.40154	-3.36982
-7.1133	-6.08067	-4.83198	-5.31674	-0.75256	-3.61904	-6.09001	-6.70255	-6.24443	-1.72204
-8.21063	-8.49824	-6.73474	-7.65028	-1.64591	-3.9328	-7.15722	-8.40994	-8.39625	-3.40034
-8.13884	-7.34111	-6.15694	-6.80788	-1.80834	-3.77086	-6.94378	-8.72128	-7.8989	-3.64445
-7.5953	-6.41127	-6.99375	-7.55345	-0.92514	-3.18382	-8.18376	-8.92215	-8.70075	-1.28466
-7.35943	-8.13664	-5.52933	-7.73743	-2.02153	-7.07041	-7.54344	-8.52042	-7.78725	-3.12571
-7.81067	-6.88652	-5.51936	-7.09837	-1.99107	-5.3599	-7.04541	-8.68111	-7.50304	-3.11554
-7.43122	-8.93217	-4.99137	-7.48568	-2.08244	-5.87609	-6.93361	-8.14881	-7.42184	-3.69531
-6.99248	-4.41888	-7.56688	-7.56134	-0.92458	-1.52172	-8.2936	-9.07022	-8.35483	-0.80086
-7.62134	-7.29224	-6.04305	-7.53855	-1.56148	-3.60499	-7.14909	-7.9045	-7.75618	-3.63236
-8.16961	-8.00233	-6.04736	-6.67233	-2.25502	-5.05626	-7.82803	-7.7772	-7.99025	-4.5192
-8.01578	-5.92569	-7.93019	-6.97249	-2.00122	-2.39436	-8.11262	-8.37981	-8.5079	-2.59679
-7.26713	-7.72338	-7.15315	-8.79284	-1.38197	-3.89231	-6.90312	-7.82742	-8.2237	-2.97313
-8.03629	-5.23348	-7.22288	-7.51472	-0.59013	-2.42472	-7.2182	-8.18898	-7.9192	-0.99986
-7.97476	-7.73371	-6.05732	-7.21456	-1.82864	-4.17571	-7.34017	-8.03833	-8.0207	-3.9496
-8.21063	-7.63039	-5.7983	-7.4179	-1.48348	-4.59068	-7.01492	-7.92785	-7.74664	-2.85108
-8.20328	-7.38434	-5.07149	-7.65249	-1.44568	-5.25992	-6.45661	-7.75505	-7.37961	-2.8549
-7.93108	-7.51327	-5.2658	-7.48158	-1.33953	-4.3059	-7.07214	-8.41264	-7.61134	-2.06784
-7.32866	-7.5684	-5.76842	-6.80788	-2.08244	-4.01377	-7.64508	-8.40994	-8.0816	-2.86125
-7.75939	-8.08498	-5.51936	-7.27266	-2.01138	-6.62507	-7.28935	-8.18898	-8.39625	-3.28845
-8.52855	-6.67989	-6.95391	-7.9214	-1.82864	-3.47734	-7.70606	-8.40994	-8.23385	-2.87142
-9.06679	-8.69209	-7.44415	-9.20204	-2.60368	-5.93163	-8.44748	-9.08019	-8.69278	-4.28505
-6.67335	-5.12801	-8.16004	-8.31332	-1.01143	-2.11555	-8.01468	-8.85103	-8.68312	-0.78166
-8.3337	-5.76039	-7.06349	-9.65461	-0.84392	-2.67775	-7.67557	-8.13877	-8.03085	-1.69152
-7.16457	-7.44443	-7.50182	-8.05696	-1.53424	-2.79921	-8.49884	-9.78589	-9.0357	-1.72204
-8.19012	-6.46293	-6.27648	-7.079	-1.08756	-2.99151	-7.4418	-8.1689	-7.73649	-1.86444
-8.30293	-7.47542	-9.25515	-8.90904	-1.8388	-4.58056	-8.18376	-9.1632	-9.30976	-2.46456
-7.57479	-5.59508	-9.33485	-10.2743	-1.30075	-2.63727	-8.23458	-9.13307	-10.1116	-1.43724
-8.01578	-6.21498	-6.07724	-7.91172	-0.68149	-3.74049	-6.42542	-8.08855	-7.8989	-1.69152
-8.2414	-6.8142	-5.90789	-8.2022	-1.98092	-4.95505	-7.1064	-8.5405	-8.25415	-3.37999
-7.81067	-6.35962	-6.27648	-8.69602	-1.6053	-2.99151	-8.11262	-8.75141	-8.72105	-2.5561
-6.44669	-7.58907	-9.14557	-9.47064	-3.19913	-6.35179	-8.32606	-9.65532	-9.47216	-4.03097
-8.69264	-5.8637	-6.03739	-7.62123	-1.2906	-3.26479	-6.76083	-8.32959	-8.19325	-2.88159
-7.61581	-4.23132	-9.49424	-8.87031	-1.585	-1.9389	-8.19393	-9.65532	-9.82741	-0.85746
-8.97979	-7.57874	-5.38986	-7.1371	-1.28045	-4.80323	-7.26902	-8.37981	-7.88875	-1.83393
-7.1133	-4.16933	-7.92023	-8.71538	-1.15863	-1.41259	-7.35033	-8.80163	-8.84285	-0.22683
-7.93373	-4.54127	-7.15315	-8.15378	-1.26014	-1.97938	-6.79132	-8.58068	-8.6906	-1.30501
-7.5181	-7.1541	-6.04305	-6.85492	-1.05968	-3.3032	-6.00457	-7.34655	-7.04166	-2.70133

-7.21585	-4.93387	-7.20296	-7.24361	-1.42257	-2.29314	-7.63492	-8.91211	-8.74135	-1.49827
-7.34917	-5.59508	-6.5355	-7.77616	-0.83377	-2.09072	-7.20804	-8.62085	-8.47745	-0.85746
-6.97998	-6.59724	-7.18303	-7.57282	-2.19411	-2.81945	-7.18771	-8.63089	-8.5079	-3.8072
-7.66709	-7.07249	-6.57535	-8.04727	-2.03168	-3.75061	-7.83819	-8.79159	-8.47745	-3.07485
-7.81845	-6.73967	-7.03507	-7.90315	-1.70623	-3.17665	-7.47609	-8.47242	-8.0555	-2.8645
-8.39523	-8.21929	-5.55921	-6.99186	-2.29562	-6.25058	-7.4418	-8.32959	-7.24929	-3.93943
-7.92348	-6.97951	-6.43588	-8.46363	-1.69667	-4.42874	-8.12278	-9.10294	-8.15265	-2.74936
-7.36968	-6.88652	-6.92402	-7.27266	-1.81849	-3.08261	-8.14311	-10.0872	-8.88345	-2.70868
-8.60688	-7.20936	-5.73624	-7.48158	-1.46498	-4.28643	-6.94711	-8.44253	-8.03619	-2.8165
-7.94047	-6.79493	-5.13285	-8.42726	-1.48428	-3.86783	-6.98558	-8.08384	-8.20034	-2.66293
-7.87477	-6.84098	-6.10441	-7.77782	-1.22373	-3.11824	-7.01444	-8.20341	-8.10378	-2.47097
-8.31319	-7.2378	-7.44205	-8.17315	-1.11802	-3.35588	-6.71001	-7.94794	-7.77709	-2.43404
-7.95425	-7.48575	-5.68872	-7.45663	-1.06726	-4.22632	-7.82803	-8.18898	-7.55379	-2.13907
-8.01578	-7.02083	-5.45959	-7.25329	-1.69667	-4.48947	-6.94378	-7.87764	-7.70604	-2.66799
-8.51302	-7.6422	-5.7976	-8.19939	-1.87993	-4.082	-7.49532	-8.58202	-8.41276	-3.9683
-1.97095	-2.9822	-1.75794	-2.41136	-2.46858	-3.60499	-1.84007	-2.34491	-2.0014	-0.69528
-6.80476	-5.65295	-7.43392	-8.22217	-1.47463	-1.99873	-7.60112	-8.65176	-8.1424	-2.78771
-7.18508	-7.65106	-7.10334	-7.81489	-2.19411	-3.78098	-7.88901	-9.03263	-8.41655	-3.89874
-8.38161	-7.10805	-6.45213	-7.90315	-1.45533	-3.80942	-7.27412	-8.48238	-8.1424	-2.01025
-7.9645	-7.83702	-6.42592	-7.50504	-1.06726	-4.0745	-7.42148	-8.53046	-7.7974	-1.95598
-7.39019	-7.81636	-5.36993	-7.66965	-1.44288	-4.89432	-6.76083	-8.60076	-7.60454	-1.56947
-8.47727	-6.74188	-6.4558	-7.47599	-1.37181	-4.13522	-8.00082	-9.03263	-8.7718	-1.34569
-8.14696	-8.23161	-5.38852	-7.88036	-1.78343	-4.33511	-7.78386	-8.76136	-8.37414	-3.46919
-7.56454	-5.99801	-7.76084	-8.6476	-1.92001	-3.01176	-8.43786	-9.65532	-9.51276	-2.22044
-7.14266	-5.82793	-7.12711	-7.9829	-1.44568	-2.77752	-7.76462	-8.79125	-8.24862	-1.32877
-7.45173	-7.7647	-5.65883	-8.19252	-1.8794	-5.09675	-7.11656	-8.46016	-8.01055	-2.89176
-8.16573	-8.68288	-6.24759	-8.70071	-2.69053	-5.06522	-8.1301	-8.75139	-8.59622	-4.05469
-8.39523	-6.78321	-5.38986	-7.80521	-1.99107	-7.74854	-7.23853	-8.61081	-8.3049	-3.41051
-7.82784	-7.6422	-5.81806	-6.54729	-1.01143	-5.07496	-5.65833	-7.10743	-7.032	-3.09486
-6.93895	-2.74358	-7.97004	-9.08333	-0.37694	-0.83567	-8.15327	-8.88198	-8.7718	0.169861
-7.9645	-4.01436	-8.00989	-9.06396	-1.10787	-2.35387	-8.7936	-9.94658	-9.47216	-1.07106
-7.65684	-8.12631	-7.73095	-8.95745	-1.98092	-3.94292	-8.40737	-8.88198	-8.5891	-3.50205
-8.12859	-8.32261	-5.11092	-7.15646	-1.30075	-5.51172	-6.77099	-7.80733	-7.37109	-2.92228
-7.92348	-4.70657	-6.52554	-8.2022	-0.90483	-2.66763	-6.95394	-8.38985	-7.76694	-0.95918
-8.2414	-5.72939	-7.05353	-7.84394	-1.2906	-2.698	-7.48246	-8.67107	-8.1831	-1.92547
-8.65162	-7.09315	-6.13701	-7.04027	-2.10274	-4.43886	-7.60443	-8.58068	-8.8733	-3.49188
-8.27216	-5.57442	-6.97383	-8.7057	-1.8591	-3.29515	-8.87491	-8.9322	-9.0357	-2.4137
-8.00552	-7.05183	-5.45959	-7.6987	-1.8794	-4.61093	-8.37688	-8.2392	-7.69589	-3.47154
-8.57872	-8.6092	-6.25782	-8.1652	-2.32383	-5.9219	-8.58213	-9.09015	-9.21418	-2.66293
-8.54118	-7.08042	-6.89189	-8.24496	-1.20443	-5.21125	-7.32221	-8.71154	-8.01688	-2.30779
-7.28345	-5.29378	-6.90212	-7.60691	-1.13688	-2.36865	-7.01444	-8.17352	-7.92032	-2.2694
-6.75436	-6.97951	-6.76463	-7.54377	-1.40227	-3.80122	-7.29951	-8.55055	-8.27445	-2.13907
-7.66709	-5.88437	-6.93398	-8.63792	-1.0165	-2.2729	-7.75688	-9.35402	-8.041	-1.23381
-7.98501	-5.91536	-5.59906	-7.00154	-0.41755	-2.34375	-6.78115	-8.45011	-7.69589	-0.98969
-8.27216	-5.73973	-6.36614	-7.57282	-1.04696	-3.01176	-7.29951	-8.85185	-8.9748	-1.52878
-7.80041	-7.54774	-5.3799	-6.74979	-1.68652	-4.83359	-7.30967	-7.83746	-7.49289	-3.8784
-7.24662	-2.85723	-8.70723	-9.13174	-0.68149	-1.22028	-7.96016	-8.71124	-9.10675	0.119004
-6.97998	-5.76039	-7.33247	-9.07364	-1.68652	-2.75872	-8.2854	-9.01254	-8.82255	-1.80341

-7.33892	-5.22315	-6.78455	-6.30438	-0.83377	-2.51581	-6.89296	-7.84751	-7.77709	-1.44741
-8.82596	-7.80603	-5.7983	-7.81489	-2.00122	-6.70604	-7.79754	-8.58068	-8.2237	-2.56627
-8.85673	-7.77504	-5.18065	-6.84661	-1.68652	-5.9773	-6.97427	-8.0082	-7.35079	-4.00046
-7.69786	-7.4031	-7.35239	-8.62824	-2.31593	-4.05425	-8.09229	-9.20337	-8.5688	-4.26492
-7.70811	-7.13448	-6.38607	-8.35712	-2.09259	-4.92469	-8.68179	-9.83611	-8.84285	-2.85108
-7.83118	-7.54774	-6.05732	-7.48568	-2.03168	-4.33765	-7.71623	-8.41998	-8.13235	-3.67497
-7.36968	-7.78537	-8.65742	-9.89668	-1.8591	-4.58056	-8.40737	-9.58502	-9.84771	-1.26432
-7.8722	-6.93818	-6.39603	-7.04995	-1.30075	-3.07248	-7.13689	-8.18898	-7.90905	-2.4137
-7.69786	-6.69023	-5.53929	-7.06932	-1.69667	-6.05827	-6.34411	-7.67677	-7.22899	-3.32914
-7.21585	-7.32045	-7.31254	-6.99186	-2.51896	-3.8417	-8.94605	-9.01254	-8.4064	-2.61713
-8.01578	-4.95453	-7.193	-8.84126	-0.56982	-2.62715	-7.37066	-8.09859	-8.0613	-0.86763
-7.77152	-5.5056	-7.08621	-8.06266	-1.34918	-2.19342	-7.57227	-8.58202	-8.83761	-1.53993
-8.22205	-6.44497	-5.76692	-7.76642	-1.24303	-4.55901	-6.60087	-7.73512	-7.94929	-3.21004
-8.13758	-7.32908	-7.06575	-9.47549	-1.73518	-5.15284	-8.40901	-9.25953	-9.28177	-1.52073
-6.86717	-4.62392	-7.33247	-9.21889	-1.26014	-2.30326	-6.64902	-8.3095	-7.838	-1.32535
-7.99679	-7.0528	-6.33964	-8.05127	-1.67728	-4.61742	-6.50469	-7.81483	-7.87205	-2.53815
-8.92851	-7.33078	-5.26035	-7.5825	-1.55455	-6.67568	-7.37066	-8.39989	-7.59439	-3.04433
-6.79538	-6.80387	-7.07345	-7.9214	-1.77788	-3.47734	-8.89523	-9.18328	-8.8936	-1.22364
-6.82614	-4.25199	-7.81065	-8.07632	-0.84392	-1.77696	-7.20804	-8.83176	-8.09175	-1.26432
-8.54906	-7.52708	-6.67497	-7.38885	-1.88955	-4.41862	-7.4723	-8.03833	-8.3049	-2.82056
-7.48994	-7.91849	-6.39077	-8.58677	-2.09223	-5.47409	-7.61074	-8.68165	-8.6445	-2.8453
-6.91844	-5.95669	-6.41595	-8.55078	-1.61546	-2.81945	-7.70606	-8.94224	-9.2184	-1.96616
-8.26898	-7.81718	-5.34762	-7.29928	-1.93783	-5.15284	-6.67781	-8.11373	-7.39892	-4.02589
-7.83722	-8.25924	-6.26805	-8.58677	-2.28523	-5.10416	-7.17794	-8.54216	-8.35483	-3.26763
-7.47117	-6.68442	-8.17027	-9.29319	-2.42033	-3.86783	-7.87042	-9.0503	-9.05004	-3.07566
-7.34915	-6.59232	-6.7794	-7.57273	-1.88958	-3.12798	-7.5915	-8.69161	-8.04585	-3.09486
-7.72862	-5.38845	-6.59527	-7.85362	-0.52922	-2.77897	-7.08607	-8.20907	-8.11205	-1.01003
-8.44732	-8.029	-5.89987	-7.31067	-2.31418	-4.85106	-7.5338	-8.85103	-8.2293	-4.01629
-8.55056	-7.98296	-5.7567	-7.50437	-2.21768	-4.9484	-7.45685	-8.5322	-8.4707	-3.93951
-7.33038	-7.13568	-7.00439	-6.44475	-2.11153	-3.75101	-7.5915	-8.38275	-8.37414	-3.18124
-7.20836	-6.29762	-6.8203	-8.03987	-1.40708	-2.52441	-7.71653	-8.59198	-8.93417	-2.18302
-8.45676	-8.22962	-5.45959	-6.14946	-2.27532	-5.18784	-8.47852	-8.48024	-7.7974	-4.46835
-7.34915	-4.88857	-7.12711	-7.83479	-1.16583	-1.8235	-7.23565	-8.25322	-8.07481	-1.07921
-8.5224	-7.66062	-6.38054	-7.96012	-2.08258	-5.04575	-7.23565	-8.61191	-8.59622	-2.88369
-6.88768	-4.9442	-7.01368	-8.50236	-1.16878	-2.57654	-7.13689	-8.83176	-8.2237	-1.36604
-7.85169	-6.15299	-7.3225	-8.82189	-1.30075	-3.63928	-8.68179	-9.66537	-9.16765	-1.09141
-6.83292	-5.34904	-7.27029	-8.11963	-1.38778	-2.52441	-8.36092	-9.26949	-8.70243	-0.63769
-7.52352	-5.93602	-6.34622	-7.73743	-1.38197	-2.99151	-7.8077	-8.44007	-8.21355	-2.04753
-8.16573	-8.34213	-6.07373	-7.69806	-2.48788	-4.14041	-8.30322	-8.95066	-9.34936	-3.32521
-8.04654	-7.64073	-5.80827	-8.06664	-1.82864	-4.51983	-8.05163	-8.7012	-8.3049	-4.04114
-8.25021	-7.37513	-6.31918	-8.10824	-1.87028	-3.81915	-7.82233	-8.59198	-8.50932	-2.60534
-8.02603	-8.09531	-6.94394	-7.70838	-2.22456	-5.86597	-8.54966	-9.35402	-9.04585	-2.8409
-6.71333	-4.65492	-9.0061	-8.43458	-1.24999	-2.05023	-7.79754	-9.24354	-9.53306	-0.46077
-8.38497	-7.86802	-5.08103	-8.01823	-2.07229	-5.79512	-7.84836	-8.33963	-8.01055	-3.77668
-7.79029	-8.35134	-7.81232	-8.87162	-2.42033	-5.90243	-8.84181	-10.2858	-9.43626	-2.00065
-7.7799	-6.19431	-5.91785	-8.67665	-1.09772	-3.6494	-8.03131	-8.96233	-8.5688	-0.81677
-7.65684	-7.39277	-5.75845	-7.62123	-1.63576	-3.23443	-7.74672	-8.29946	-7.88875	-1.88478
-9.01986	-7.72509	-7.00439	-9.09949	-1.81238	-3.92624	-7.88965	-8.4226	-8.17137	-2.70133

-7.09279	-3.63209	-7.9003	-8.50236	-0.80331	-1.57453	-7.76705	-9.41428	-8.90375	-0.55232
-8.0568	-6.57658	-5.85808	-8.41522	-0.85407	-3.55831	-7.50279	-8.48024	-8.82255	-2.24079
-8.29714	-7.9277	-6.04305	-7.32207	-2.03433	-4.84132	-7.07214	-8.09381	-7.81411	-3.69955
-7.90293	-7.50406	-7.0453	-7.86897	-2.14048	-3.76075	-7.76462	-9.08019	-8.58656	-4.13147
-7.97476	-6.8452	-7.53171	-9.56746	-2.41745	-3.88219	-9.09851	-9.61515	-9.1778	-3.24776
-8.40549	-7.24813	-5.99755	-7.39853	-1.38197	-4.1251	-7.06574	-8.20907	-7.67559	-2.77988
-6.76461	-3.93171	-10.859	-9.25762	-1.63576	-2.26278	-9.47457	-9.60511	-9.54321	-0.89815
-7.80907	-7.85402	-6.2885	-6.72959	-2.07293	-3.90677	-7.55303	-8.60194	-8.16171	-3.4308
-7.73398	-6.88703	-6.29873	-7.47019	-1.63868	-3.99438	-6.87017	-7.96428	-7.67893	-2.8357
-6.21082	-4.17967	-8.89651	-8.35712	-0.98605	-1.54416	-8.00082	-9.09289	-9.0763	-0.60317
-6.22282	-4.75963	-7.90437	-7.70945	-0.90528	-2.0182	-7.62036	-8.51227	-8.45138	-1.1464
-6.65458	-4.45572	-7.86346	-7.90315	-1.03073	-1.03497	-7.54341	-8.5322	-8.7314	-1.26158
-9.14188	-6.72125	-6.49304	-8.25635	-1.96678	-4.10147	-7.93774	-9.32927	-8.20034	-2.19262
-8.37223	-7.07122	-6.74872	-7.50437	-2.28523	-4.43245	-7.54341	-8.77132	-8.48035	-4.38103
-7.52352	-6.00834	-5.48948	-7.71806	-0.90483	-3.13321	-7.79754	-8.55055	-8.2034	-1.95598
-6.3749	-4.08668	-8.07962	-7.82457	-0.4277	-1.24052	-8.03131	-9.60511	-8.59925	-0.05391
-8.39523	-7.06216	-6.44584	-7.63092	-1.13832	-3.55831	-7.86869	-8.99246	-8.76165	-1.69152
-7.00187	-6.39892	-5.65443	-7.45879	-1.05003	-2.69964	-7.428	-7.95432	-8.20034	-2.44217
-7.06202	-6.23564	-7.39224	-7.70838	-1.84895	-2.5563	-7.97032	-8.96233	-8.61955	-1.59998
-8.41574	-6.85553	-6.03739	-7.04027	-1.3109	-4.52995	-7.72639	-8.08855	-7.82785	-3.00365
-7.13381	-6.27696	-7.68114	-9.0446	-1.90986	-3.05224	-8.37688	-8.96233	-8.84285	-1.82375
-9.0003	-8.93217	-8.97621	-9.13174	-2.85397	-7.43477	-9.46441	-10.9007	-11.1774	-2.57645
-8.13884	-6.59724	-5.96766	-7.38885	-1.18908	-3.54819	-7.42148	-9.09289	-8.447	-1.73221
-7.34917	-5.16116	-6.03739	-7.40821	-0.66119	-2.67775	-6.67952	-7.34533	-7.35079	-0.90832
-7.47224	-8.02299	-6.9041	-7.53409	-2.03168	-5.26881	-7.95	-8.5405	-8.1425	-2.45439
-6.82614	-5.58475	-6.30637	-7.08868	-1.81849	-2.51581	-8.05163	-9.25359	-7.61469	-1.72204
-7.99527	-6.78321	-6.40599	-7.66965	-0.78301	-3.51782	-8.0618	-8.96233	-7.76694	-0.70489
-7.67735	-6.58691	-5.31016	-7.17583	-0.96574	-2.94091	-7.82803	-8.17894	-8.6094	-1.71187
-7.61581	-4.88221	-6.79451	-8.34744	-0.7424	-1.2304	-8.04147	-8.02829	-8.63985	-0.6642
-7.24662	-5.88437	-7.24281	-8.99618	-1.08756	-2.50569	-9.37293	-9.11298	-9.0357	-0.89815
-7.66709	-6.14265	-5.98758	-8.30871	-0.96574	-3.51782	-8.24475	-8.44007	-7.9192	-1.42707
-7.49275	-7.10349	-5.69868	-7.1371	-0.66119	-3.5077	-7.61459	-8.4702	-7.51319	-1.28466
-9.16439	-9.29377	-5.51936	-7.1371	-1.5647	-5.38015	-7.6959	-8.04837	-7.72634	-3.55291
-8.35421	-7.7957	-7.15315	-8.4249	-2.09259	-4.78299	-8.26508	-9.11298	-8.5485	-2.18993
-8.20038	-5.80171	-5.88796	-7.74711	-0.96574	-3.33564	-7.92967	-8.05842	-7.59439	-1.92547
-8.21063	-6.76255	-8.87659	-9.66429	-1.20939	-4.81335	-8.50901	-9.67541	-9.2793	-1.0202
-7.93373	-5.62608	-9.33485	-10.1	-1.52409	-1.99962	-8.55983	-9.12302	-9.04585	-1.49827
-9.23618	-6.90719	-6.46576	-8.78316	-1.84895	-6.27082	-7.8077	-7.71694	-8.0613	-2.70868
-8.25165	-7.66139	-5.65883	-8.05696	-1.13832	-4.80323	-7.73656	-7.78724	-8.0816	-2.50524
-7.98501	-4.86155	-6.76463	-7.40821	-1.0165	-1.92877	-9.21031	-9.2335	-8.8124	-0.93883
-7.70582	-6.92386	-7.89414	-8.87162	-1.24303	-2.95275	-8.52443	-9.13	-8.83761	-1.36716
-6.39541	-3.19817	-7.07345	-7.67933	-0.50891	-0.83567	-7.50279	-8.57063	-7.9192	-0.07425
-7.83118	-4.90287	-7.56159	-9.11238	-1.69667	-1.9389	-7.85852	-8.34968	-8.3455	-1.51861
-8.27837	-6.7673	-6.50327	-8.01709	-1.55183	-4.9192	-7.41838	-8.5322	-8.5576	-2.56695
-7.54626	-7.00675	-6.07373	-8.47284	-1.26233	-4.09173	-7.50494	-8.81118	-8.21965	-2.02944
-8.46609	-6.79493	-5.51125	-7.51576	-1.62903	-4.2767	-7.89927	-8.50231	-8.20999	-2.24061
-7.99679	-5.95687	-6.47259	-7.78921	-1.05968	-3.48817	-7.13947	-8.18348	-7.81411	-1.7415
-7.88415	-8.39738	-6.59531	-8.00569	-1.43603	-4.98734	-7.73577	-8.65176	-7.7948	-3.33481

-6.92678	-5.87398	-6.04305	-6.5359	-0.35523	-1.75535	-6.37005	-7.50597	-6.75199	-2.12543
-8.0531	-6.40813	-5.47034	-7.15116	-1.27198	-3.26426	-7.33182	-8.26319	-7.84308	-1.91426
-7.86538	-5.80951	-7.49529	-7.83479	-0.98248	-2.37839	-8.08201	-9.02041	-8.86658	-1.88547
-6.56072	-5.32141	-7.63846	-7.91454	-1.01143	-1.69694	-7.36068	-8.74143	-8.59622	-1.86627
-8.17512	-8.25924	-6.51349	-9.48688	-2.33348	-7.70338	-8.07239	-9.49865	-8.95348	-3.72834
-6.4387	-4.58465	-7.99641	-7.82339	-1.20443	-1.60933	-8.48596	-9.14993	-8.80864	-0.69528
-8.27837	-7.57774	-5.68511	-7.85757	-1.97643	-5.89269	-7.33182	-7.94436	-7.71755	-3.18124
-8.29714	-7.22778	-6.44191	-8.05127	-1.86063	-4.06253	-7.73577	-8.70158	-8.49001	-1.77989
-8.02494	-6.02133	-6.72826	-7.72085	-1.62903	-3.25453	-7.64921	-8.50231	-8.20034	-2.39418
-7.38669	-3.70975	-8.81457	-7.23092	-1.00178	-1.76509	-8.0243	-9.04033	-8.43207	-0.66649
-8.99005	-6.77288	-6.25656	-9.13174	-1.55455	-5.17772	-8.71228	-9.45446	-8.82255	-2.52559
-8.23114	-7.14481	-6.0872	-7.66965	-1.43272	-3.91255	-7.60443	-8.44007	-7.99025	-2.29164
-7.27738	-6.39061	-7.96008	-8.87031	-1.93016	-3.53807	-8.62081	-9.08285	-9.14735	-1.97633
-8.40977	-7.3475	-5.09194	-8.74629	-1.87993	-5.08469	-7.8608	-8.32297	-8.03619	-3.44999
-7.57441	-6.03054	-5.88965	-7.96012	-1.46498	-3.2448	-6.2931	-7.34655	-7.34098	-3.24843
-8.31319	-7.47542	-4.91168	-7.09837	-2.26517	-7.3538	-7.48246	-8.48024	-8.0207	-4.01063
-7.94399	-8.36393	-8.47811	-10.7294	-2.722	-7.95096	-9.43392	-10.6396	-10.1319	-2.0577
-7.57441	-5.73584	-8.72253	-9.19064	-1.37813	-2.36865	-8.44748	-9.70788	-9.24315	-1.82788
-7.58505	-6.22531	-7.46197	-8.86062	-1.23984	-2.77897	-10.1149	-9.60511	-9.02555	-1.39655
-7.93373	-6.36995	-6.83436	-8.57014	-1.3109	-3.41661	-9.04769	-8.85185	-8.41655	-0.99986
-8.3337	-6.2873	-6.13701	-7.96981	-1.51394	-3.77086	-8.38704	-9.51472	-8.80225	-3.37999
-7.75275	-6.98833	-6.48281	-7.9829	-1.72553	-2.8846	-8.05316	-9.5684	-8.79899	-2.91249
-7.80041	-3.7974	-9.90268	-9.85795	-0.7018	-2.29314	-9.45425	-9.98676	-10.4364	-0.78626
-7.95924	-7.36592	-5.23512	-8.40447	-1.86063	-4.89973	-7.70692	-8.43256	-7.82377	-3.47879
-7.8722	-6.42161	-6.58531	-8.73475	-1.68652	-3.74049	-8.64114	-8.74137	-9.2184	-2.4137
-8.12819	-8.13031	-5.57261	-8.47284	-1.95713	-6.5936	-6.8317	-7.89454	-8.0555	-3.92991
-8.04654	-7.41343	-5.93777	-7.0209	-1.52409	-4.15547	-8.32606	-8.66102	-8.37595	-2.1289
-8.11833	-6.46293	-5.04118	-6.46899	-1.10787	-1.85793	-7.96016	-8.71124	-8.35565	-1.99667
-7.15204	-4.4373	-6.83053	-6.77517	-0.75088	-1.5996	-7.94736	-8.48238	-8.20034	-0.45532
-9.26694	-3.94204	-9.41454	-10.497	-0.80331	-1.54416	-9.21031	-10.5693	-9.93906	0.210547
-7.57479	-7.27912	-5.94773	-7.48568	-1.97077	-5.31942	-8.13295	-8.92215	-8.8327	-2.80022
-8.23114	-7.27912	-6.2466	-8.15378	-1.8591	-4.33765	-7.24869	-8.22916	-7.19854	-2.60696
-10.0669	-7.91968	-8.67735	-9.40286	-2.19411	-4.25668	-9.43392	-9.4645	-8.9951	-3.68514
-7.00049	-5.8637	-7.6114	-9.02523	-2.1332	-2.94091	-7.81787	-9.27367	-8.11205	-2.64765
-6.94921	-5.02685	-6.97383	-8.84126	-1.15863	-2.16157	-8.4277	-9.62519	-8.8733	-1.18295
-8.56933	-6.8594	-9.14183	-8.98555	-1.81238	-4.22802	-8.1878	-9.10011	-9.53282	-1.31917
-7.69643	-7.48564	-5.44989	-7.56134	-1.87028	-4.31564	-8.38016	-8.59198	-8.65415	-2.91249
-7.93108	-4.59386	-8.73275	-8.10824	-1.20443	-1.96952	-8.1301	-9.31931	-8.98245	-0.51291
-6.51379	-3.93999	-6.71803	-6.7182	-0.73158	-1.78456	-7.98583	-8.46246	-8.63484	-0.0042
-7.25529	-4.57544	-8.65094	-8.06266	-1.58078	-1.32702	-8.21666	-8.69161	-8.46104	-1.53993
-7.39608	-5.46876	-7.79187	-8.03987	-1.41673	-3.20586	-8.13972	-8.59198	-8.97279	-1.61672
-6.62642	-5.33983	-7.74073	-7.39043	-1.09828	-1.93058	-8.46672	-8.98055	-8.76037	-0.55131
-8.25021	-7.31987	-6.08396	-7.4474	-1.97643	-4.58821	-8.67831	-9.20971	-8.89554	-2.17342
-8.7289	-8.09347	-5.68511	-8.5412	-1.81238	-6.94406	-8.21666	-9.03037	-9.04038	-3.79553
-8.17512	-7.23699	-6.44191	-6.91189	-1.90888	-4.33511	-8.1878	-8.44253	-7.95895	-3.46919
-6.96432	-4.58465	-7.85323	-7.37904	-1.39743	-2.51468	-7.98583	-8.62187	-8.27758	-1.28078
-7.77152	-6.85019	-6.06351	-8.32472	-1.39743	-3.11824	-8.01468	-8.71154	-8.1424	-2.24061
-8.16573	-7.50406	-6.87144	-8.57538	-1.60008	-3.51737	-8.26475	-9.02041	-8.65415	-2.36538

-8.51302	-8.58157	-5.59306	-8.01709	-2.17908	-5.58117	-8.11086	-8.75139	-8.37414	-4.10268
-8.59749	-7.04359	-7.12711	-7.90315	-1.89923	-3.87756	-7.72615	-8.63183	-8.27758	-2.45177
-8.02494	-6.35287	-6.63622	-7.8006	-1.09828	-3.17665	-7.52418	-8.47242	-8.69278	-1.67431
-8.15635	-5.93845	-7.09643	-8.23357	-1.64833	-3.19612	-8.38978	-9.13997	-8.62519	-1.67431
-8.19389	-6.91465	-5.85897	-7.77782	-1.95713	-4.1112	-8.03392	-8.59198	-7.93963	-3.14285
-7.64012	-4.72279	-7.9555	-7.86897	-1.26233	-2.34918	-8.64946	-8.8311	-8.9921	-1.77029
-7.5181	-6.32525	-6.37032	-7.54994	-1.47463	-4.16961	-7.90889	-8.80121	-8.7314	-1.7031
-8.41916	-5.42272	-10.1645	-9.84009	-1.65798	-3.68287	-9.37079	-10.1263	-11.155	-0.91604
-8.71012	-7.30145	-5.61352	-8.18799	-2.23698	-5.88296	-7.92812	-8.8311	-8.29689	-3.86272
-7.1802	-6.39892	-6.44191	-7.83479	-1.76413	-3.8873	-7.90889	-9.03037	-8.66381	-1.95266
-7.79968	-5.84635	-6.60554	-7.19674	-1.58078	-3.43949	-7.8608	-8.5322	-8.19068	-2.02944
-7.40547	-5.84635	-7.76119	-8.59817	-1.60008	-3.35188	-8.55328	-9.17982	-9.06935	-1.55913
-9.22636	-7.09884	-7.69983	-9.21343	-2.41068	-6.7883	-9.11111	-9.9769	-9.79352	-3.21004
-7.03941	-4.05971	-7.03507	-7.0714	-0.81843	-2.10581	-8.11086	-8.44253	-8.4707	-0.14818
-8.42854	-7.17252	-6.12487	-8.79186	-1.52288	-3.9165	-8.37054	-8.95066	-8.75071	-2.23101
-7.41485	-5.22932	-6.91235	-7.49297	-1.34918	-2.58282	-8.14933	-8.33293	-8.45138	-1.20399
-7.55564	-6.05817	-5.48057	-7.8006	-1.16583	-3.10851	-7.101	-7.96428	-7.95895	-1.59752
-8.51302	-6.62916	-6.52372	-7.59552	-2.00538	-4.04306	-8.37054	-8.69161	-8.44173	-2.78771
-8.19389	-6.33446	-6.16578	-7.8006	-0.81843	-3.40055	-7.428	-8.35286	-8.28724	-1.60712
-8.34407	-6.86861	-6.47259	-7.67527	-1.92818	-4.15988	-7.36068	-8.46246	-8.39345	-3.17164
-8.33468	-6.94228	-6.176	-8.15381	-1.16583	-3.72181	-7.33182	-8.5322	-8.33552	-3.21963
-7.856	-7.61458	-6.25782	-8.26775	-1.44568	-4.31564	-7.70692	-8.313	-7.99757	-2.8549
-8.65162	-6.91752	-5.86804	-7.50504	-2.03168	-4.71214	-7.20804	-8.32959	-7.64514	-2.83073
-8.60034	-6.94851	-6.5355	-7.1371	-1.93016	-4.23644	-6.89296	-8.53046	-8.07145	-3.12571
-7.79016	-7.24813	-7.71102	-8.39585	-1.98092	-4.06437	-7.66541	-9.09289	-8.84285	-2.52559
-7.2979	-5.29547	-8.14936	-8.00854	-1.66622	-2.54618	-7.30967	-8.76146	-7.8583	-2.01701
-8.35346	-7.08042	-7.44415	-8.42726	-1.34918	-4.14041	-8.61099	-9.10011	-9.67765	-1.38636
-8.59008	-6.22531	-6.48569	-7.87298	-1.06726	-2.81945	-7.88901	-8.73133	-8.4064	-2.54593
-8.43625	-6.31829	-7.88038	-8.78316	-1.33121	-3.8417	-8.4277	-9.73567	-8.6094	-2.22044
-6.56976	-3.34281	-8.36852	-9.21889	-0.45815	-1.39234	-7.57393	-9.0728	-8.51805	-0.6642
-8.02603	-6.74188	-6.65504	-8.19252	-1.54439	-3.85183	-6.84214	-8.91211	-8.09175	-2.39336
-6.71333	-3.67342	-8.29879	-7.24361	-0.9962	-1.22028	-6.66935	-8.73133	-8.1831	-1.9153
-8.62565	-7.11726	-7.1578	-8.46144	-2.01503	-3.92624	-8.36092	-8.86099	-8.71209	-2.62454
-6.39541	-6.54558	-4.86187	-6.53677	-1.24999	-2.66763	-5.54116	-6.59208	-6.52864	-3.17656
-8.28775	-6.26078	-6.26805	-7.23092	-1.33953	-3.14745	-7.74539	-8.33293	-8.12309	-1.89507
-6.14928	-6.10133	-7.01368	-7.22424	-2.11289	-3.54819	-8.14311	-8.75141	-8.76165	-2.47473
-8.12859	-7.02083	-7.14319	-8.04727	-2.24487	-4.21619	-8.14311	-8.88198	-8.8327	-2.93245
-6.97998	-5.39879	-7.25277	-7.6406	-0.98605	-2.43484	-7.42148	-8.50033	-8.7109	-2.0577
-7.89354	-7.12647	-6.51349	-7.77782	-1.24303	-3.11824	-7.74539	-8.41264	-8.82796	-2.32699
-7.91322	-8.07465	-5.51936	-6.73042	-1.57485	-5.83561	-6.98443	-8.25929	-7.66544	-2.96296
-8.76443	-7.02083	-6.85428	-8.68633	-1.68652	-3.8417	-7.4723	-8.68111	-8.4064	-2.68833
-8.09782	-6.85553	-5.57914	-6.98217	-1.16878	-4.16559	-7.60443	-8.03833	-7.8989	-2.33233
-7.66709	-7.961	-6.55542	-7.96013	-1.8794	-4.15547	-7.61459	-8.46016	-8.13235	-3.75634
-8.62085	-7.84736	-5.81823	-8.17315	-1.07741	-4.6008	-7.07591	-8.51037	-8.01055	-2.63747
-7.5953	-6.39061	-5.84811	-8.27966	-0.83377	-2.83969	-6.72017	-7.95798	-7.74664	-1.69152
-9.17465	-8.31227	-6.58531	-8.19252	-1.6053	-6.34167	-6.76083	-8.19903	-7.59439	-3.10536
-8.72341	-7.4031	-5.878	-7.70838	-2.1332	-5.451	-7.60443	-8.37981	-7.8583	-4.5904
-8.01556	-7.14489	-6.37032	-7.91454	-1.40708	-3.94571	-7.2645	-8.67169	-8.26793	-2.56695

-6.79538	-4.53094	-6.13701	-7.25329	-0.82362	-2.05023	-7.71623	-8.96233	-7.8177	-1.29484
-8.426	-7.80603	-5.43967	-7.61155	-1.36166	-4.41862	-6.83197	-7.66672	-7.67559	-3.29862
-6.79537	-2.84406	-8.07823	-6.23966	-0.14293	-0.39247	-6.92788	-8.18348	-8.45138	0.274148
-6.89793	-4.40696	-7.34243	-8.28934	-0.90483	-1.39234	-8.67163	-9.41428	-8.853	-1.14226
-7.93373	-5.35746	-6.86425	-8.24093	-1.2703	-2.26278	-8.37688	-8.86189	-8.3049	-2.00684
-7.36968	-7.08282	-6.50561	-7.8633	-1.68652	-4.14534	-7.8077	-9.58502	-8.45715	-1.93564
-7.79016	-5.35746	-6.38607	-8.41522	-0.31603	-1.47332	-8.4277	-8.7715	-8.68045	-1.37621
-8.68238	-7.00017	-5.27031	-8.04727	-1.5647	-5.06638	-7.65524	-8.63089	-8.17295	-3.57325
-7.67735	-8.16763	-7.7708	-8.67665	-1.61546	-4.41862	-8.09229	-9.20337	-8.9342	-2.18993
-8.426	-6.58691	-5.14081	-7.32107	-1.23984	-3.94292	-7.92967	-8.33963	-7.59439	-3.42068
-7.52748	-4.29916	-7.82255	-7.92594	-1.03073	-1.47304	-8.55328	-9.42891	-8.80864	-1.43435
-7.34917	-4.41729	-7.60144	-9.60619	-1.55455	-1.45307	-9.14933	-9.47454	-9.0763	-0.91849
-7.56454	-7.94034	-5.12088	-7.57282	-1.59515	-4.8842	-7.20804	-8.80163	-8.80225	-3.16639
-7.79016	-7.7957	-6.46576	-6.18819	-1.88955	-3.94292	-7.97032	-8.51037	-8.19325	-3.69531
-7.80041	-7.29979	-4.99137	-7.42758	-1.50379	-3.38625	-7.15722	-8.13877	-7.43199	-2.65782
-7.21585	-4.86155	-8.03977	-8.65729	-1.16878	-2.16157	-8.33622	-9.73567	-10.2943	-0.99986
-7.22713	-5.61612	-7.1987	-8.02848	-1.48428	-2.94301	-8.06277	-9.33924	-9.01141	-1.11761
-7.98501	-8.5189	-6.47573	-7.3017	-2.29562	-4.89432	-8.22442	-8.58068	-8.3252	-4.21406
-8.37472	-7.70271	-6.06728	-7.48568	-1.81849	-4.49959	-7.71623	-8.35972	-8.2846	-3.58342
-7.503	-8.33294	-7.35239	-8.96713	-2.48851	-4.38826	-8.04147	-9.13307	-8.9342	-4.12251
-7.9645	-5.3058	-6.95391	-8.6476	-0.77286	-2.92066	-6.6287	-7.90777	-7.68574	-2.3425
-6.73384	-5.3058	-9.94253	-10.284	-1.69667	-3.66964	-7.90934	-10.3483	-10.0101	-0.67437
-8.908	-7.73371	-6.09717	-8.39585	-2.38699	-6.52386	-8.09229	-8.84181	-8.041	-4.94641
-7.31841	-6.22531	-5.44963	-6.64328	-1.48348	-2.66763	-7.71623	-9.35402	-8.21355	-2.09838
-6.93895	-4.09701	-6.39603	-6.94344	-1.15863	-1.64538	-8.21426	-9.41428	-8.86315	-1.11175
-7.73888	-7.74404	-7.79072	-8.46363	-2.19411	-3.68988	-8.56999	-9.35402	-8.59925	-2.85108
-7.48055	-7.32908	-7.55665	-8.19939	-0.06574	-2.47574	-6.43737	-7.64545	-7.17684	-1.82788
-7.57441	-7.21857	-6.95326	-7.53855	-1.42638	-3.63419	-7.08176	-8.313	-7.74652	-1.96226
-7.64012	-6.39892	-6.22714	-7.91454	-1.02108	-3.40055	-7.65883	-8.48238	-8.46104	-1.65511
-9.69566	-8.18556	-6.24759	-7.812	-2.26593	-7.97596	-5.66795	-7.17717	-7.66928	-4.97612
-8.55995	-7.03438	-5.87942	-7.24231	-1.49393	-4.62715	-6.47584	-8.08384	-7.86239	-2.58615
-8.00617	-4.81489	-7.9555	-8.42726	-0.85703	-1.99873	-8.5629	-9.22964	-9.1659	-0.50331
-7.93108	-7.55932	-5.29648	-7.23092	-1.15618	-3.40055	-6.73552	-8.26319	-7.98791	-2.70133
-7.56503	-4.62149	-6.18623	-6.33081	-0.72193	-2.23236	-7.73577	-8.32297	-8.3838	-0.5801
-7.7152	-6.98833	-6.32941	-8.45005	-1.05003	-4.34484	-7.14909	-8.90085	-8.35483	-2.19262
-8.19389	-5.5056	-6.34986	-7.37904	-1.26233	-2.67044	-7.51456	-8.29308	-7.7948	-1.7319

C1QB	ZNF296	SCARF1	ETV7	GBP1	STAT1	TAP1	TRAFD1	culture_po	any_positi
-10.1945	-6.26765	-8.48215	-9.67503	-2.15893	-0.41756	-3.72743	-3.16853	0	0
-8.73628	-6.40109	-7.52832	-7.55056	-0.98194	0.627872	-2.97546	-2.53629	1	1
-8.29682	-6.39083	-8.58471	-7.28372	-2.16907	-0.20646	-3.82014	-2.99517	0	0
-9.13579	-6.29844	-9.3847	-6.93477	-1.43853	0.406723	-3.23299	-2.74024	0	0
-9.29559	-6.61666	-8.73856	-7.45819	-1.65161	0.386618	-3.44931	-2.77083	0	0
-11.8924	-6.88355	-7.58986	-7.98161	-3.65045	-1.14132	-3.89225	-3.2807	0	0
-7.9073	-6.50374	-7.91806	-6.58582	-1.19502	0.537402	-3.01667	-2.51589	1	1
-7.9474	-6.1353	-8.50642	-6.16868	-1.64912	0.17852	-3.31483	-3.10888	0	0
-9.5353	-6.92461	-7.31294	-7.85845	-1.42839	0.537402	-3.23299	-2.5057	0	0
-7.76747	-6.51401	-8.16421	-6.49345	-1.49941	0.326305	-3.01667	-2.56688	0	0
-8.31679	-6.93487	-10.0616	-7.6224	-2.09805	0.054894	-3.53172	-3.03596	0	0
-9.22568	-7.07858	-9.53854	-6.97582	-2.36186	-0.28688	-3.74804	-3.11754	0	0
-8.36673	-6.80143	-10.0616	-7.47872	-1.8241	-0.04563	-3.62443	-2.91359	0	0
-10.2644	-5.79546	-10.1026	-7.69424	-2.26039	-0.1562	-3.67593	-3.43366	0	0
-9.54529	-6.9862	-8.4001	-7.15029	-1.60087	0.21573	-3.22269	-2.79122	1	1
-9.39547	-6.71931	-7.70268	-6.99635	-0.79931	0.698238	-2.90336	-1.78168	0	0
-10.9536	-8.72098	-8.66676	-9.66477	-2.53434	-0.55829	-4.27339	-3.45406	0	0
-9.78499	-7.72528	-9.08727	-7.35556	-1.65161	0.637925	-3.45961	-2.46491	0	0
-8.3967	-8.07429	-7.66166	-6.87319	-1.09355	0.597716	-3.69653	-2.42412	0	0
-11.0934	-8.08455	-9.40521	-7.2324	-1.01238	0.3062	-3.85105	-2.5057	0	0
-8.62642	-6.06235	-7.63089	-7.0374	-1.68205	0.014685	-3.3463	-2.81162	0	0
-7.64762	-7.22229	-8.59497	-7.28372	-0.83989	0.909335	-3.14028	-2.29155	0	0
-7.34798	-6.43189	-7.87704	-5.63135	-0.75872	1.070171	-2.87245	-2.22017	1	1
-7.37795	-6.65772	-6.61552	-5.73398	0.742946	2.025135	-2.59433	-1.7001	1	1
-9.40546	-6.34977	-8.32831	-6.63714	-2.16907	-0.07578	-3.54202	-2.80142	0	0
-10.3143	-6.46268	-8.55395	-9.15161	-2.75757	-0.45777	-3.53172	-2.77083	0	0
-7.83738	-6.90408	-7.26166	-7.05793	-0.48477	0.456984	-2.73854	-2.37313	0	0
-9.06588	-8.00243	-9.49752	-8.32029	-2.42273	-0.48793	-4.04677	-3.34188	1	1
-8.40668	-6.58586	-7.3232	-6.12398	-0.78916	1.060119	-2.64583	-1.98563	1	1
-8.50656	-6.69878	-8.02062	-6.44214	-0.78916	0.838969	-2.97546	-2.22017	0	0
-10.8476	-7.44614	-8.00906	-7.44266	-1.38816	0.208328	-3.65814	-2.57517	0	0
-9.21569	-6.83222	-8.27703	-8.47424	-1.50956	0.376566	-3.3669	-2.5057	0	0
-9.59019	-6.82817	-7.18325	-6.32793	-1.42682	0.317625	-3.12926	-2.67047	0	0
-9.78499	-7.88952	-8.45138	-9.35687	-2.38215	-0.25673	-4.45881	-3.13794	0	0
-7.52776	-5.90837	-8.87189	-6.68845	-2.16907	-0.32709	-3.82014	-3.01557	0	0
-9.06588	-6.53454	-9.18983	-6.69871	-1.04282	0.517298	-3.16088	-2.43431	0	0
-8.08708	-6.87328	-8.44113	-7.71477	-1.03268	0.728395	-3.18148	-2.40372	0	0
-8.86612	-6.90408	-7.70268	-6.54477	-1.07326	0.557507	-3.41841	-2.51589	0	0
-9.8437	-6.79072	-9.38854	-8.14522	-2.48035	-0.31828	-4.09423	-3.28995	1	1
-8.98597	-6.07261	-11.4462	-8.70003	-2.35171	-0.02552	-3.93345	-2.8728	0	0
-10.0047	-6.61666	-9.16932	-9.53135	-2.81844	-0.60855	-4.3661	-3.37248	0	0
-10.8071	-6.91244	-9.57622	-9.9063	-2.50934	-0.54681	-4.0664	-3.33761	0	0
-6.79866	-6.90408	-7.25141	-6.02135	-1.19502	0.286096	-3.06817	-2.37313	1	1
-9.17442	-7.67085	-7.54923	-7.38645	-0.82757	0.675323	-3.27772	-2.50845	0	0
-9.22568	-6.39083	-8.73856	-8.71029	-2.41259	-0.27683	-3.80984	-3.14813	0	0
-10.6854	-6.94989	-8.41258	-8.24826	-2.30637	-0.00033	-3.87155	-2.75625	0	0
-6.07954	-9.11105	-7.60012	-8.00214	-1.70234	-0.18636	-3.87165	-3.11754	1	1

-9.32556	-6.61666	-8.53343	-7.12977	-1.37765	0.3062	-3.16088	-2.4751	1	1
-5.55019	-7.54051	-7.44627	-5.3645	0.144308	1.050067	-2.59433	-1.83267	0	1
-8.18695	-6.49348	-8.34882	-7.4274	-1.07326	0.597716	-3.4081	-2.5057	0	0
-8.35303	-6.25702	-9.10701	-9.08197	-2.0454	-0.13943	-3.76948	-2.99451	0	0
-11.9324	-7.21203	-8.98471	-8.0124	-1.84439	0.12526	-3.75834	-2.93399	0	0
-7.60766	-6.87328	-8.236	-7.79687	-0.79931	0.859074	-3.12998	-2.39352	0	0
-6.94847	-9.57298	-7.34371	-6.63714	-0.98194	-0.26678	-3.51111	-2.81162	1	1
-9.31557	-7.48918	-7.58986	-8.57687	-1.37765	0.245887	-3.50081	-2.4751	0	0
-8.6364	-7.59183	-6.19502	-5.10792	0.976314	1.542627	-2.24409	-1.57773	1	1
-9.3755	-7.47892	-8.91291	-6.93477	-0.90077	0.698238	-3.53172	-2.78103	0	0
-7.68757	-6.40109	-8.20523	-7.3145	-1.19502	0.497193	-2.74884	-2.23037	0	0
-8.26686	-7.44812	-7.50781	-5.92898	-0.1195	1.11038	-2.87245	-2.03662	0	0
-4.00209	-7.65342	-8.06165	-6.76029	0.661775	1.251112	-3.49051	-2.28135	1	1
-8.27684	-6.21632	-8.3078	-8.08424	-1.42839	0.235835	-3.18148	-2.883	0	0
-10.1845	-6.10341	-8.41036	-9.34661	-2.68654	-0.3874	-4.00556	-3.20932	0	0
-9.29559	-6.10341	-7.40525	-9.13108	-1.79366	0.115208	-3.17118	-2.46491	0	0
-8.71631	-6.7501	-8.37959	-6.8424	-2.02702	-0.01547	-3.65533	-2.93399	0	0
-8.49657	-7.08885	-9.48726	-8.85398	-1.50956	0.265991	-3.48021	-2.62807	0	0
-6.93849	-5.84678	-8.36933	-7.57108	-1.27619	0.336357	-2.97546	-2.28135	0	0
-12.6415	-6.40109	-9.9693	-6.68845	-0.99209	0.79876	-3.53172	-2.51589	0	0
-9.31557	-7.05805	-9.26162	-8.75135	-2.20966	-0.26678	-3.67593	-3.04616	0	0
-8.50656	-6.36003	-7.09756	-5.24135	-0.85004	0.818865	-2.66643	-2.27116	0	0
-8.04713	-6.3703	-10.6872	-9.07977	-2.71698	-0.48793	-4.09827	-3.51524	0	0
-10.614	-7.37627	-10.3693	-8.71029	-1.956	-0.06573	-3.96436	-3.18892	0	0
-11.7526	-6.34977	-9.25137	-7.65319	-1.88497	0.165469	-3.64503	-2.9034	0	0
-10.1845	-7.44812	-9.92828	-9.38766	-1.8241	0.115208	-3.85105	-3.20932	0	0
-9.21569	-6.61666	-9.41547	-8.36135	-1.58058	0.43688	-3.41841	-2.61787	0	0
-8.46661	-6.02129	-9.24111	-7.98161	-2.42273	-0.27683	-3.73774	-3.21952	0	0
-10.0247	-6.55507	-8.13344	-8.71029	-1.89512	0.195626	-3.55232	-2.55668	0	0
-9.57525	-7.07858	-8.42061	-7.93029	-2.17922	0.064946	-3.61412	-2.8932	0	0
-8.85613	-7.07858	-8.00011	-6.54477	-1.59073	0.095103	-3.3463	-2.66885	0	0
-10.4741	-7.47892	-7.93857	-6.80135	-1.18487	0.507245	-3.51111	-2.64846	0	0
-9.19572	-6.34977	-8.91291	-7.73529	-2.40244	-0.1361	-3.77894	-3.13794	0	0
-6.26931	-6.47295	-7.75396	-6.31898	-1.13414	0.416775	-3.11968	-2.59747	0	0
-9.11582	-6.3703	-8.46164	-7.61214	-0.64711	1.040014	-3.03727	-2.108	0	0
-7.94725	-6.48321	-9.30265	-7.39661	-1.88497	-0.1562	-3.57292	-3.07675	0	0
-10.5241	-7.25309	-8.96419	-8.9874	-2.31112	-0.56835	-4.397	-3.39287	0	0
-9.5353	-6.18553	-10.3693	-7.59161	-2.29083	-0.14615	-3.55232	-3.2807	0	0
-8.49657	-6.20606	-9.13855	-6.19582	-1.79366	0.175521	-3.3566	-2.9034	0	0
-8.97599	-7.46865	-9.53854	-9.33635	-1.68205	0.356462	-4.13947	-3.21952	0	0
-8.98597	-6.10341	-9.18983	-7.82766	-2.02702	0.105155	-3.55232	-2.98497	0	0
-8.83616	-6.71931	-7.4873	-8.08424	-1.34721	0.517298	-3.16088	-2.4853	0	0
-10.8137	-6.5756	-8.67702	-8.39214	-3.38664	-1.14132	-4.3661	-3.63761	0	0
-10.3	-6.51919	-8.03721	-10.637	-3.27291	-1.04362	-3.96433	-2.88967	0	0
-9.81328	-6.33193	-8.68472	-8.49182	-2.02607	-0.17918	-3.68598	-2.94686	0	0
-8.14007	-6.12594	-8.21551	-6.88061	-0.93389	0.585899	-2.86018	-2.32737	0	0
-6.25391	-7.71767	-7.11756	-5.5317	-0.35396	0.615707	-2.62822	-2.0224	NA	NA
-9.87488	-7.3968	-7.72319	-6.1445	0.479139	1.753724	-2.48102	-1.74089	0	0

-7.97721	-7.89978	-6.92321	-6.87319	-0.64711	0.265991	-3.26389	-2.46491	0	0
-9.02593	-6.10341	-8.58471	-6.50371	-1.5197	0.276044	-3.72743	-2.65866	0	0
-8.09706	-6.76037	-8.34882	-6.9245	-2.1082	-0.26678	-3.71713	-2.8728	0	0
-9.77272	-5.59224	-9.77329	-9.17564	-2.24838	-0.08975	-3.59319	-2.88014	0	0
-10.0247	-6.49348	-8.57446	-6.82187	-0.79931	1.050067	-2.96516	-2.25076	1	1
-7.31802	-8.0127	-7.1591	-5.82635	-0.03833	0.768604	-2.68704	-1.95504	1	1
-5.65007	-7.55077	-6.29758	-4.94371	0.550164	1.140537	-1.68784	-1.74089	1	1
-8.77893	-6.23829	-8.83487	-5.92513	-0.85656	0.963469	-2.9808	-2.34644	0	0
-7.71416	-7.05288	-6.88295	-5.36308	-0.79857	0.675323	-3.1571	-2.34644	1	1
-8.68767	-7.47422	-8.03721	-7.5457	-1.92942	-0.06988	-3.17565	-2.5847	0	0
-11.7806	-6.91244	-7.67123	-7.70495	-2.30637	-0.45739	-3.53752	-2.87061	0	0
-5.70631	-6.17275	-7.17386	-5.46612	0.254956	1.599378	-1.9416	-1.07888	0	0
-8.6364	-6.07261	-7.9078	-7.71477	-1.62117	0.175521	-3.3566	-2.67905	0	0
-5.44032	-5.66201	-6.86167	-5.27214	0.499432	1.803985	-1.71874	-0.82312	0	0
-9.07301	-6.36001	-9.05071	-7.84546	-2.46102	-0.36796	-3.7602	-3.27089	0	0
-8.00824	-6.80944	-7.94337	-6.82441	-1.21418	0.138776	-3.25916	-3.23277	0	0
-8.16035	-7.13715	-6.52635	-4.76356	0.515921	1.410592	-2.10862	-1.87944	0	0
-10.2798	-6.64091	-8.71288	-9.1007	-2.606	-0.54681	-3.87155	-3.16606	0	0
-8.66637	-6.22659	-9.46675	-6.4524	-2.45317	-0.55829	-4.09827	-3.46425	0	0
-6.13946	-7.49945	-5.57965	-4.81029	0.976314	1.612992	-1.67754	-1.3024	0	1
-6.6788	-6.79116	-6.38989	-6.23687	-0.98194	0.597716	-3.3566	-2.16918	0	0
-6.77108	-7.51168	-7.08002	-5.37245	-0.05434	1.033022	-2.69317	-2.52752	0	1
-8.58646	-6.94514	-7.85652	-6.80135	0.063137	1.33153	-2.87245	-2.30175	0	0
-11.0707	-6.93116	-7.3334	-6.47781	-1.02087	0.655451	-3.06431	-2.81343	0	0
-9.39547	-6.48321	-7.02577	-5.99056	-0.12965	1.381791	-2.13078	-1.42477	0	0
-10.1074	-7.4555	-7.54923	-8.44498	-1.47515	0.416986	-3.49113	-2.74672	0	0
-7.76747	-6.71931	-8.36933	-5.84687	0.540018	2.00503	-2.54282	-1.28201	0	0
-9.1258	-6.72957	-7.1591	-7.12977	-1.88497	0.165469	-3.336	-3.02576	0	0
-8.03714	-5.88784	-9.94879	-8.06372	-2.38215	-0.14615	-3.78924	-2.97478	0	0
-8.56649	-6.19579	-8.54369	-8.24845	-2.47347	-0.16626	-3.92315	-3.06655	0	0
-5.65007	-6.67825	-6.30784	-4.52292	-0.16008	1.241059	-2.12048	-1.40438	0	0
-5.39038	-7.64316	-6.01041	-4.5024	-1.00224	0.396671	-2.61493	-2.54649	1	1
-8.65725	-6.99671	-6.98618	-6.74947	-0.78891	0.78462	-2.75812	-2.3655	1	1
-8.18063	-7.26824	-10.0079	-6.91808	-1.87143	0.099031	-3.59319	-3.15653	0	0
-7.63304	-6.52855	-6.61081	-4.52938	0.438598	1.549697	-2.06223	-1.71742	0	0
-9.1947	-6.48174	-7.53985	-6.78694	-0.52794	1.201935	-2.62822	-2.23207	0	0
-5.99025	-7.12779	-7.49293	-5.55043	-0.57627	0.794556	-2.80451	-2.62282	1	1
-12.2268	-6.57537	-8.67534	-7.69558	-2.3547	0.099031	-3.8066	-3.10888	1	1
-9.63075	-6.89371	-8.55334	-7.19911	-1.94875	-0.16924	-3.3705	-3.06122	0	0
-6.0511	-6.566	-6.76096	-6.24362	-0.86623	0.446794	-2.96225	-2.74672	1	1
-8.4766	-6.77063	-6.96423	-6.4524	-1.74292	-0.16626	-3.3772	-2.8932	0	0
-8.71631	-6.82196	-8.70779	-6.36003	-1.60087	0.406723	-3.01667	-2.67905	0	0
-9.6152	-6.97593	-7.28218	-7.51977	-1.83424	0.014685	-3.3154	-2.84221	0	0
-7.71753	-5.7544	-7.9078	-6.93477	-1.06311	0.748499	-3.00637	-2.41392	0	0
-7.50779	-6.61666	-7.25141	-7.39661	-1.87483	0.175521	-3.3257	-2.74024	0	0
-8.58646	-6.60639	-5.83605	-5.27214	-0.61667	1.029962	-2.42951	-1.74089	0	0
-8.30681	-6.24712	-7.82575	-5.99056	-1.22546	0.678134	-2.88275	-2.46491	0	0
-9.85491	-7.65342	-7.9078	-7.3145	-2.01688	0.366514	-3.69653	-2.81162	0	0

-7.73751	-7.79713	-9.1488	-7.30424	-1.02253	0.79876	-3.52142	-2.46491	0	0
-9.1258	-6.34977	-7.22064	-7.18108	-2.40244	-0.64876	-3.65533	-3.10734	0	0
-7.21814	-7.44812	-6.11297	-5.77503	-0.60653	0.567559	-2.70764	-2.36293	1	1
-8.68634	-8.64913	-6.44117	-5.73398	-0.99209	-0.08584	-3.3978	-2.4751	1	1
-8.65725	-6.16339	-7.58677	-6.7682	-1.18519	0.585899	-3.1571	-2.19395	0	0
-8.44664	-6.93487	-8.63599	-8.4024	-2.38215	-0.22657	-3.74804	-2.79122	0	0
-9.70509	-7.00673	-7.9796	-8.61793	-1.59073	0.205678	-3.3257	-2.8932	0	0
-7.87733	-6.06235	-8.18472	-7.18108	-0.89063	0.778656	-2.88275	-2.18958	0	0
-9.74504	-8.15641	-8.71804	-10.0445	-2.66625	-0.56835	-4.19098	-3.31129	0	0
-7.91728	-6.68851	-6.35912	-6.4524	-0.34272	0.899283	-2.42951	-2.01622	0	0
-9.11582	-6.9554	-8.0719	-6.20608	-1.05297	0.828917	-2.84155	-2.20997	0	0
-10.0247	-7.84846	-6.95398	-5.00529	-0.33257	1.01991	-2.24409	-1.76129	0	0
-10.7438	-7.24282	-8.57446	-8.09451	-2.38215	-0.1361	-3.72743	-2.8728	NA	1
-8.15021	-6.94989	-7.85891	-6.00007	-0.68259	0.585899	-2.95297	-2.57517	NA	NA
-10.7838	-7.81766	-8.49241	-7.75582	-1.73278	0.446932	-4.19098	-2.95438	0	0
-11.3131	-6.72957	-10.6565	-8.78214	-2.31112	0.165469	-3.69653	-3.14813	0	0
-12.0023	-6.68851	-8.84112	-7.32477	-1.27619	0.859074	-3.30509	-2.20997	0	0
-9.40546	-6.39083	-10.0001	-6.71924	-1.41824	0.678134	-2.79005	-2.60767	0	0
-7.9073	-7.48918	-7.46679	-6.01108	-0.05862	1.371739	-2.84155	-1.7001	0	0
-9.64517	-6.84249	-10.1231	-9.34661	-1.79366	0.21573	-3.19178	-3.07675	0	0
-7.8274	-6.05208	-8.4001	-6.86293	0.032697	1.311425	-2.83125	-1.85306	0	0
-7.45051	-6.36938	-8.61903	-6.32793	-1.42682	0.347433	-3.3705	-2.81343	0	0
-9.21569	-8.10508	-8.7283	-6.75003	-1.18487	0.346409	-3.75834	-3.15833	0	0
-7.17819	-7.27362	-8.43087	-6.70898	-0.61667	0.838969	-3.24329	-2.06721	0	0
-9.13579	-6.56533	-8.7283	-9.61345	-1.93571	0.396671	-3.54202	-2.75043	0	0
-12.1321	-7.43786	-8.97445	-6.9245	-1.36751	0.859074	-3.16088	-2.36293	0	0
-10.614	-7.70475	-8.16421	-8.49477	-1.70234	0.286096	-3.83044	-2.98497	1	1
-8.62642	-7.29415	-9.65136	-8.00214	-1.8038	-0.07578	-4.05707	-3.00537	0	0
-10.4542	-7.72528	-9.01547	-9.14135	-2.62566	-0.58845	-4.00556	-3.33169	0	0
-9.19572	-6.52427	-8.70779	-8.55635	-1.8241	0.21573	-3.42871	-3.00537	0	1
-7.53775	-7.17097	-8.21549	-7.25293	-1.57044	0.316253	-3.30509	-2.73004	0	0
-12.2919	-6.81169	-8.58471	-8.65898	-1.97629	0.165469	-3.69653	-3.14813	0	0
-9.47537	-8.02296	-9.20009	-8.26898	-1.52985	0.426827	-3.65533	-2.75043	0	0
-9.00595	-7.62263	-8.61548	-7.37608	-1.76322	0.105155	-3.67593	-2.78103	0	0
-10.0567	-6.89371	-8.00906	-7.26468	-1.26251	0.466666	-3.22205	-2.70859	0	0
-8.81618	-6.7809	-8.32831	-6.11371	-1.29648	0.386618	-3.66563	-2.69945	0	0
-8.65725	-6.63154	-8.34689	-7.70495	-1.30117	0.45673	-3.08287	-3.00404	0	0
-10.1276	-6.25702	-8.92871	-8.00471	-2.28704	-0.15931	-3.68598	-2.93733	0	0
-10.9997	-7.13715	-9.27593	-9.99061	-2.66399	-0.40771	-4.26125	-3.11841	0	0
-9.7423	-7.67085	-7.50231	-7.18037	-1.56214	0.297753	-3.60247	-2.57517	0	0
-9.58005	-6.80008	-8.43135	-7.30215	-1.0692	0.705132	-3.47257	-2.44174	0	0
-8.89048	-6.41619	-8.67534	-6.65579	-1.76511	0.2282	-3.53752	-2.84202	NA	0
-9.98567	-7.38996	-9.10701	-9.30678	-1.68779	-0.07982	-3.92722	-2.97545	0	0
-8.8702	-6.95925	-7.94337	-6.98365	-0.67292	1.122446	-3.08287	-1.96522	0	0
-9.26568	-7.90493	-7.85891	-8.06091	-2.0454	-0.24873	-3.98289	-3.00404	0	1
-6.13222	-7.2776	-7.69938	-5.56917	0.177633	1.470209	-2.71173	-2.1463	0	0
-9.65103	-7.47422	-9.39792	-7.95787	-1.50414	0.486538	-3.88083	-2.77531	0	0
-10.4217	-7.37123	-8.74103	-8.50118	-1.50414	0.287817	-3.84371	-2.66094	0	0

-4.72267	-6.82817	-7.57739	-6.3654	-0.36363	0.625643	-2.99008	-2.47033	0	0
-12.1659	-7.5304	-9.73576	-10.8243	-2.30637	-0.31828	-4.48393	-3.32808	0	0
-10.0668	-6.50046	-8.66595	-8.89462	-2.41269	-0.18911	-3.90866	-3.29948	0	0
-10.8476	-6.55664	-8.59088	-8.22016	-1.76511	0.496474	-3.86227	-2.83249	0	0
-8.34288	-7.4555	-9.30408	-7.28341	-0.8469	0.585899	-3.74165	-2.55611	0	0
-7.49107	-7.66149	-8.17798	-7.38645	-0.03501	1.152255	-3.27772	-2.29878	0	0
-7.85613	-7.56786	-9.08824	-8.05154	-0.46995	1.420528	-3.46329	-2.28925	1	1
-9.61047	-7.0248	-8.47827	-7.8642	-0.85656	0.8343	-3.44473	-2.2416	1	1
-9.01217	-6.28511	-9.8859	-8.08901	-2.15172	-0.24873	-3.62103	-2.90873	0	0
-9.7423	-7.07161	-8.42197	-9.64401	-2.36436	-0.41764	-3.94578	-3.30902	0	0
-9.68145	-7.56786	-9.46361	-8.50118	-1.45582	0.387177	-4.00145	-2.81343	0	0
-7.86627	-7.7083	-9.1164	-8.2951	-1.33983	0.347433	-4.0664	-2.88014	0	0
-10.3406	-6.77199	-8.6378	-9.30678	-2.40302	-0.4872	-3.9365	-3.2423	0	0
-8.73837	-5.77014	-8.66595	-7.31151	-0.89522	1.132383	-3.04575	-2.05099	0	0
-10.1986	-8.39181	-7.99968	-6.58085	-1.24318	0.337497	-3.73237	-2.59423	0	0
-9.26568	-6.18212	-8.16859	-6.17805	-1.19485	0.45673	-3.25916	-2.55611	0	0
-7.95754	-6.63154	-8.90994	-8.38877	-2.84763	-0.75547	-4.3262	-3.147	0	0
-9.25554	-7.49295	-8.34689	-9.22248	-1.98741	0.248072	-4.21485	-2.86108	0	0
-9.15414	-6.93116	-8.90056	-9.06323	-1.71678	0.277881	-4.21485	-2.86108	0	0
-9.49892	-8.3169	-7.62431	-5.39118	0.602909	1.460273	-2.53543	-1.00264	0	0
-9.07301	-6.77199	-8.48765	-6.94618	-0.94355	0.73494	-3.38906	-2.33691	0	0
-9.05273	-6.94989	-8.69411	-7.06796	-1.94875	-0.50707	-4.23341	-3.38526	0	0
-7.0753	-7.13715	-8.53458	-5.92513	-0.88556	0.486538	-3.49113	-2.60376	0	0
-10.1545	-6.63719	-7.35397	-6.39082	-1.78351	0.185573	-3.3463	-2.60767	0	0
-9.34553	-6.19579	-10.0308	-8.61793	-3.0721	-1.03075	-4.12917	-3.73958	0	0
-7.86735	-6.84249	-6.09246	-5.1695	-1.04282	0.487141	-2.81065	-2.19977	1	1
-7.88732	-6.40109	-7.55909	-5.6724	-0.64711	0.838969	-2.51192	-1.82247	0	0
-10.2344	-6.61666	-7.12833	-6.30871	-1.93571	0.095103	-3.27419	-2.63826	0	0
-10.6539	-6.7809	-8.29754	-6.78082	-1.06311	0.698238	-2.91366	-2.24056	0	0
-7.70754	-7.18123	-6.77962	-6.79108	-0.9718	0.577611	-2.71794	-2.28135	1	1
-8.22691	-6.86302	-6.94372	-7.33503	-0.74858	0.688186	-2.56342	-1.85306	0	0
-7.94725	-6.80143	-8.16421	-6.32924	-1.61102	0.115208	-3.24329	-2.45471	0	0
-8.65638	-6.72957	-7.0668	-6.2574	-1.22546	0.356462	-2.98576	-2.4853	1	1
-8.38671	-7.12991	-7.26166	-6.78082	-1.35736	0.497193	-3.07847	-2.39352	0	0
-9.46539	-6.21632	-8.05139	-9.02845	-2.60537	-0.16626	-3.53172	-2.85241	0	0
-7.13824	-6.23685	-6.38989	-5.64161	-0.93121	0.758551	-2.72824	-2.06721	0	0
-10.8037	-6.58586	-8.85137	-9.50056	-2.79815	-0.44772	-4.02616	-3.40307	0	0
-10.8537	-7.51998	-7.92832	-7.96108	-1.29648	0.497193	-3.4081	-2.4751	0	0
-4.96091	-7.24282	-8.4719	-6.36003	-0.69784	0.557507	-3.3154	-2.5057	0	0
-10.4741	-6.45242	-8.05139	-6.61661	-1.27619	0.507245	-3.08877	-2.42412	0	0
-12.6914	-6.63719	-11.4975	-8.39214	-2.29083	-0.06573	-3.94376	-2.95438	0	0
-9.87488	-7.17097	-8.53343	-7.17082	-1.52985	-0.05568	-3.46991	-2.78103	0	0
-8.58626	-6.8469	-6.6765	-5.87829	0.196963	1.559633	-2.42409	-1.38386	0	0
-7.10572	-7.10906	-6.75157	-4.90408	-1.02087	0.685259	-2.95297	-2.05099	0	0
-9.44541	-7.58157	-7.82575	-8.49477	-1.42839	0.085051	-3.28449	-2.4955	0	0
-10.2844	-6.71931	-8.73856	-6.61661	-1.31678	0.647977	-3.27419	-2.53629	0	0
-8.07709	-7.1607	-6.7386	-4.33819	0.103722	1.793933	-2.19259	-1.28201	0	0
-5.99025	-7.59594	-6.07591	-5.46612	-0.41196	0.764748	-2.88802	-2.38456	1	1

-10.8836	-8.06402	-8.22575	-8.83345	-2.71698	-0.69902	-3.48021	-2.92379	0	0
-7.64318	-6.22893	-6.40436	-6.67453	-0.45062	1.162191	-2.70245	-1.73648	0	0
-4.67127	-7.38653	-8.08216	-5.85714	-0.09921	0.999805	-2.96516	-2.35274	NA	0
-10.3743	-8.20773	-8.53343	-7.37608	-1.99658	-0.09589	-4.09827	-3.22971	0	0
-9.55528	-7.3968	-7.84627	-7.6224	-0.80945	0.456984	-3.20208	-2.41392	0	0
-8.12703	-7.03752	-7.68217	-5.89819	-0.36301	1.140537	-2.83125	-1.99583	0	0
-7.84737	-6.04182	-8.98471	-7.61214	-1.91541	-0.00542	-3.58322	-3.05636	0	0
-6.78867	-6.41136	-9.20009	-5.9495	-0.21082	1.291321	-2.85185	-2.15898	0	0
-9.00203	-6.63154	-7.28648	-7.60191	-0.8179	0.705132	-2.90658	-2.07005	0	0
-9.44541	-7.08885	-9.30265	-9.27477	-2.39229	0.004633	-4.17038	-3.17873	0	0
-8.12703	-6.40109	-7.98985	-7.12977	-0.71814	0.939492	-2.95486	-2.23037	0	0
-8.79621	-6.25738	-8.16421	-7.21187	-0.32243	1.241059	-2.75914	-1.88365	0	0
-8.97599	-6.67825	-8.55395	-7.98161	-1.68205	0.286096	-3.70683	-2.75043	0	0
-10.4521	-7.01543	-8.2249	-8.23889	-3.31157	-0.78528	-4.40043	-3.39479	0	0
-9.80497	-5.94943	-8.08216	-6.55503	-1.50956	0.477089	-3.16088	-2.66885	0	0
-7.06516	-7.19333	-8.29059	-7.03049	-0.69225	0.466666	-2.97153	-2.4608	0	0
-5.59477	-7.00607	-6.07591	-4.63242	0.322613	1.490081	-2.25708	-1.47916	0	0
-7.47782	-7.01699	-8.97445	-6.81161	-0.9515	0.879178	-3.3566	-2.26096	0	0
-9.73216	-6.3881	-8.97563	-6.88998	-1.59113	0.446794	-3.53752	-2.78484	0	0
-8.82617	-4.96399	-6.95398	-7.37608	-0.41374	0.758551	-1.94536	-1.85306	0	0
-11.0834	-7.26335	-9.0565	-8.53582	-1.83424	0.326305	-3.68623	-2.70964	0	0
-8.62682	-6.72518	-8.44073	-7.9204	-2.1034	-0.36796	-3.97361	-2.99451	0	0
-8.59645	-6.68851	-10.0103	-8.95661	-1.76322	0.135312	-3.87165	-3.15833	0	0
-10.0547	-6.08288	-9.36419	-7.92003	-1.55014	0.577611	-3.10938	-2.19977	0	0
-12.1421	-8.8031	-9.45649	-9.84951	-2.27054	-0.14615	-4.30429	-2.97478	0	0
-8.86612	-6.41136	-7.75396	-8.16635	-0.91092	0.668081	-2.73854	-2.32214	0	0
-9.32556	-7.17097	-8.05139	-5.58003	-1.07326	0.627872	-3.06817	-2.19977	0	0
-11.383	-6.97593	-8.96419	-8.05345	-2.55464	-0.28688	-4.18068	-3.11754	0	0
-9.39547	-7.07858	-8.16421	-7.43766	-1.48926	0.336357	-3.72743	-2.77083	0	0
-9.65515	-6.81169	-8.44113	-6.86293	-0.36301	1.11038	-2.98576	-2.23037	0	0
-9.96539	-6.98734	-7.50231	-7.5176	-1.59113	0.218264	-3.4169	-2.52752	0	1
-7.52149	-6.30384	-7.20202	-6.95555	-0.99188	0.585899	-3.05503	-2.48939	NA	0
-5.26054	-8.78257	-7.62063	-5.84687	0.519725	1.210903	-2.54282	-1.93464	1	1
-9.43542	-6.88355	-8.32831	-7.54029	-1.8241	-0.04563	-3.79954	-3.00537	0	0
-8.75865	-6.95925	-9.48238	-8.92272	-2.71232	-0.53688	-4.11279	-3.28995	0	0
-8.4766	-8.76204	-9.24111	-10.7732	-3.69104	-1.26195	-4.85024	-3.6886	0	0
-7.8274	-6.40109	-6.00015	-4.61529	-0.98194	0.627872	-2.60463	-1.88365	0	0
-9.06588	-7.45839	-9.41547	-8.9053	-2.0879	-0.1562	-4.19098	-3.2807	0	0
-7.63763	-7.38653	-7.40525	-5.61082	-0.10935	1.150589	-2.79005	-1.93464	0	0
-5.95969	-6.73984	-9.33342	-8.27924	-2.45317	-0.26678	-3.89225	-3.15833	0	0
-6.98842	-8.97761	-7.0668	-7.76608	-1.50956	-0.53819	-3.82014	-3.35208	1	1
-11.2432	-7.37627	-9.75392	-9.53135	-3.9447	-1.463	-4.71633	-3.83136	0	0
-8.77623	-7.40706	-9.24111	-8.82319	-2.09805	-0.25673	-4.32489	-3.52544	1	1
-8.70632	-6.73984	-7.63089	-7.24266	-2.61552	-0.79955	-3.96436	-3.30109	0	0
-10.9835	-8.24879	-9.88725	-8.63845	-2.89962	-0.76939	-4.4176	-3.47445	1	1
-7.9474	-7.65212	-8.85364	-7.19911	-1.27217	0.307689	-4.14063	-2.8039	0	0
-8.90607	-7.49945	-8.44113	-7.14003	-0.89063	0.728395	-3.41841	-2.4955	0	0
-8.97599	-6.79116	-8.73856	-5.93924	-1.77336	0.024737	-3.66563	-3.33169	0	0

-4.26177	-8.23853	-7.52832	-6.5345	-0.24126	0.738447	-3.10938	-2.17938	1	1
-7.70754	-7.14017	-8.85137	-7.90977	-2.60537	-0.24667	-3.99526	-3.21952	NA	NA
-9.18573	-7.54051	-9.20009	-8.0124	-1.35736	0.487141	-3.90255	-2.79122	NA	0
-8.68634	-6.82196	-9.37444	-6.09319	-1.1037	0.668081	-3.11968	-2.46491	0	0
-8.65725	-6.45365	-7.88706	-6.82441	-1.31084	0.466666	-3.12926	-2.37503	0	1
-6.45907	-6.9554	-5.87708	-5.66214	-0.74858	0.517298	-3.01667	-2.28135	0	1
-10.7139	-6.77063	-7.66166	-6.12398	-1.12399	0.647977	-3.26389	-2.29155	0	0
-10.2245	-6.96567	-8.86163	-8.2074	-1.60087	0.406723	-3.66563	-2.883	NA	0
-4.10196	-7.61236	-6.74885	-5.17977	0.722653	0.999805	-2.08958	-1.93464	1	1
-5.75993	-7.99217	-6.4104	-5.87766	0.529872	1.190798	-2.17199	-1.48596	NA	1
-9.48536	-6.86302	-7.75396	-6.85266	-0.13979	1.241059	-3.03727	-2.108	0	0
-8.60654	-8.00792	-8.42197	-8.68853	-1.33983	0.595835	-3.83443	-2.40362	0	0
-9.30625	-7.67085	-9.45423	-6.81504	-0.46028	1.261551	-3.73237	-2.3655	0	1
-8.38345	-7.2776	-7.66184	-7.68621	-2.33537	-0.19905	-3.85299	-3.10888	0	0
-7.36938	-6.67836	-8.98502	-7.42392	-1.10786	0.725004	-3.5004	-2.27972	0	0
-9.60033	-6.57537	-8.20613	-6.83377	-1.12719	0.665387	-3.38906	-2.40362	NA	0
-8.33274	-7.14652	-9.24778	-6.38414	-1.44615	0.45673	-3.57463	-2.5847	0	0
-8.86006	-7.97047	-7.06125	-8.43561	-0.63426	0.605771	-3.20349	-2.22254	1	1
-7.86627	-7.52104	-8.39381	-7.5738	-2.02607	-0.04007	-4.12207	-3.10888	0	1
-7.87641	-7.52104	-9.50115	-6.64642	-0.68259	0.8343	-3.6303	-2.76578	0	0
-5.91927	-8.13901	-7.39909	-8.33257	-0.97255	0.138776	-3.47257	-2.84202	NA	NA
-10.0364	-6.99671	-9.08824	-7.09606	-1.0692	0.963469	-3.33339	-2.27972	0	0
-9.50906	-6.95925	-8.38443	-6.78694	-0.93389	0.824364	-3.25916	-2.45127	0	0
-5.5542	-7.37123	-7.29586	-4.95091	0.844544	1.301296	-2.34986	-1.87944	0	0
-8.57648	-6.65772	-8.87189	-7.56082	-0.49492	0.889231	-2.93426	-2.18958	0	0
-7.39792	-6.82196	-8.91291	-5.93924	-0.52536	1.150589	-2.95486	-2.4751	0	0
-7.63763	-6.66798	-6.995	-5.87766	-1.24575	0.567559	-2.82095	-2.19977	0	0
-7.36796	-6.80143	-6.23604	-5.76477	-0.80945	0.577611	-2.62523	-1.64911	0	0
-3.89222	-7.43786	-7.96934	-5.77503	-0.14994	1.060119	-2.98576	-2.15898	NA	NA
-9.4685	-6.98734	-7.3897	-6.42161	-1.66845	0.327561	-3.22205	-2.29878	0	0
-9.05589	-7.25309	-7.71294	-7.68398	-1.2559	0.637925	-3.46991	-2.32214	0	0
-9.28561	-7.72528	-7.56935	-6.81161	-1.64146	0.21573	-3.58322	-2.4853	0	0
-9.03591	-8.16667	-8.93342	-7.33503	-1.55014	0.43688	-3.51111	-2.56688	1	1
-8.37672	-7.84846	-7.61037	-7.76608	-1.01238	0.517298	-3.49051	-2.36293	0	0
-8.56649	-7.63289	-8.57446	-6.05214	-0.1195	1.351634	-3.23299	-2.33234	0	0
-8.42666	-6.69878	-10.1026	-10.1677	-2.6561	-0.65882	-4.22188	-3.2909	0	0
-8.33274	-6.61282	-7.28648	-5.70031	-0.73091	0.854173	-2.52615	-1.85085	0	0
-8.03714	-6.91434	-8.5642	-8.68977	-2.09805	-0.11599	-3.99526	-3.17873	0	0
-9.28561	-7.15044	-8.18472	-7.19135	-0.36301	1.170694	-2.99606	-1.91425	0	0
-10.6839	-7.83819	-10.0103	-8.4024	-1.77336	0.43688	-3.88195	-2.82182	0	0
-8.01716	-6.73984	-7.20013	-6.78082	-1.91541	-0.11599	-3.54202	-2.64846	0	0
-9.60521	-7.21203	-8.67702	-6.9245	-1.24575	0.678134	-3.72743	-2.75043	0	0
-6.20938	-6.92461	-6.80013	-6.28819	0.245772	1.592888	-2.62523	-1.95504	0	0
-8.94602	-7.3968	-7.78473	-7.57108	-0.61667	0.758551	-3.27419	-2.33234	0	0
-9.02593	-6.81169	-8.25652	-8.33056	-2.31112	-0.50803	-3.86135	-2.93399	0	1
-9.40546	-6.9554	-9.32316	-8.97714	-1.94585	0.245887	-4.00556	-2.99517	0	0
-5.92972	-8.03323	-6.43091	-6.29845	0.418261	1.090276	-2.52222	-1.67971	1	1
-9.33555	-7.43786	-7.57961	-6.1445	-0.96165	0.698238	-3.23299	-2.53629	0	0

-7.36796	-6.7501	-8.26677	-5.22082	-0.45433	1.070171	-2.96516	-1.79188	0	0
-8.20693	-7.63289	-9.01547	-8.85398	-1.19502	0.507245	-3.79954	-2.92379	0	0
-9.06588	-7.71501	-7.73345	-7.79687	-2.55464	-0.50803	-4.10857	-2.91359	0	0
-3.87085	-7.43677	-7.13633	-6.16868	-0.63426	0.188456	-2.91585	-2.60376	1	1
-9.43808	-6.78135	-7.72753	-7.18974	-2.3547	-0.1295	-3.58391	-2.82296	0	0
-7.28826	-6.45365	-7.28648	-5.31624	-1.18519	0.685259	-2.84163	-2.10817	0	0
-12.4417	-6.70904	-8.93342	-10.4242	-2.73727	-0.64876	-4.17038	-3.2603	0	0
-7.05502	-6.73454	-6.09468	-6.53401	-1.07887	0.536218	-3.0272	-2.2416	0	1
-10.7039	-6.62692	-8.84112	-8.11503	-2.85903	-0.56835	-4.25279	-3.22971	0	0
-9.08585	-6.87328	-7.09756	-6.44214	-1.66175	0.024737	-3.43901	-2.39352	0	0
-7.80742	-7.29415	-6.4104	-5.41582	-0.70799	0.838969	-2.60463	-1.81227	0	0
-10.0161	-6.72518	-9.58561	-7.5457	-1.43649	0.387177	-3.6303	-2.62282	0	0
-10.1745	-6.93487	-9.27188	-7.0374	-1.29648	0.567559	-3.65533	-2.80142	0	0
-8.68767	-6.85626	-8.16859	-7.33962	-1.3205	0.17852	-3.21277	-2.72766	0	1
-8.05895	-5.74205	-8.34689	-8.11712	-1.53314	0.397113	-3.03648	-2.43221	0	0
-8.45662	-6.01102	-9.28214	-6.82187	-1.69219	0.085051	-3.3978	-2.73004	0	0
-9.79498	-6.71931	-9.09752	-7.06819	-0.71814	1.009858	-2.98576	-2.07741	0	0
-9.91484	-7.82793	-8.4719	-7.90977	-1.17473	0.497193	-3.69653	-2.82182	0	0
-7.55773	-7.47892	-7.85652	-6.24714	-1.05297	0.92944	-3.04757	-2.0978	0	0
-8.04713	-7.21203	-8.90266	-6.08293	-0.74858	1.040014	-2.67673	-2.17938	0	0
-7.36796	-7.07858	-8.00011	-7.01687	-1.03268	0.788708	-3.43901	-2.69945	0	0
-11.3231	-7.22229	-8.44113	-7.01687	-1.41824	0.21573	-3.49051	-2.57708	NA	0
-4.96091	-6.9862	-6.98475	-6.12398	-0.86019	0.12526	-3.27419	-2.27116	0	0
-9.27562	-6.73984	-8.5642	-7.56082	-1.66175	0.185573	-3.74804	-2.84221	0	0
-7.79743	-6.9862	-8.91291	-8.04319	-1.05297	0.567559	-3.43901	-2.52609	0	0
-6.88855	-6.31897	-8.34882	-5.88792	-0.90077	0.959596	-2.86215	-1.97543	0	0
-10.5441	-7.73554	-10.2872	-9.50056	-1.5197	0.899283	-3.3772	-2.85241	0	0
-11.0235	-6.9862	-9.33342	-6.09319	-1.16458	0.61782	-3.3669	-2.38333	0	0
-8.50656	-6.32924	-9.70264	-8.52556	-2.49376	-0.42761	-4.00556	-3.15833	0	0
-7.52776	-6.72957	-8.17447	-6.2574	-0.8196	1.100328	-2.62523	-2.20997	1	1
-9.88487	-6.9862	-8.04113	-7.37608	-1.02253	1.241059	-3.03727	-2.15898	1	1
-9.65515	-6.99646	-8.35908	-8.75135	-3.18371	-0.44772	-4.4073	-3.35208	0	0
-11.403	-6.31897	-8.8924	-8.86424	-1.21531	0.959596	-3.24329	-2.22017	0	0
-10.1146	-7.07858	-9.09752	-5.33371	-0.35287	1.51247	-2.61493	-1.90405	0	0
-8.42666	-6.52427	-7.25141	-8.09451	-1.97629	-0.16626	-3.71713	-2.99517	0	0
-8.64639	-7.68422	-8.71804	-7.16056	-1.15443	0.627872	-3.3978	-2.46491	0	0
-5.40037	-7.38653	-7.86678	-7.12977	-1.24575	0.245887	-3.82014	-2.91359	0	0
-9.49535	-6.65772	-8.67702	-6.87319	-0.93121	0.748499	-3.57292	-2.45471	0	0
-7.9872	-6.7501	-8.95394	-6.51398	-0.90077	0.688186	-3.3566	-2.60767	0	1
-9.55528	-7.82793	-8.52318	-8.35108	-2.05746	0.145364	-3.63473	-2.83201	0	0
-9.33555	-7.23256	-8.95394	-9.62372	-2.07776	0.03479	-3.95406	-3.07675	0	0
-8.50656	-7.34547	-6.42065	-6.39082	-2.13863	0.03479	-3.23299	-2.44451	0	1
-9.80497	-7.38653	-9.58982	-8.99766	-1.93571	-0.01547	-3.99526	-3.09715	0	0
-8.94602	-7.04779	-8.27703	-7.7045	-0.8196	0.668081	-3.27419	-2.59747	0	0
-9.85491	-6.90408	-8.7283	-9.92135	-3.15328	-0.8297	-4.51031	-3.35208	0	0
-10.4941	-6.93487	-8.46164	-6.59608	-1.35736	0.537402	-3.52142	-2.4751	0	0
-0.5663	-0.81693	-0.64641	-0.61266	-1.17473	-1.17148	1.22734	-0.77214	0	1
-8.22691	-7.05805	-8.43087	-7.90977	-1.27619	0.487141	-3.46991	-2.66885	0	0

-6.33922	-7.43786	-8.19498	-6.4524	-1.45882	-0.02552	-3.75834	-2.55668	0	0
-6.90852	-6.9862	-9.24111	-7.22214	-1.44868	0.346409	-3.61412	-2.70964	0	0
-9.43542	-7.10938	-8.55395	-9.39793	-1.60087	0.265991	-3.55232	-2.71984	NA	NA
-11.7726	-7.06832	-8.05139	-6.32924	-1.30663	0.517298	-3.46991	-2.61787	0	0
-9.88487	-6.5756	-7.85652	-7.57108	-2.29083	-0.46782	-3.69653	-2.95438	0	0
-7.68757	-6.43189	-7.7437	-7.96108	-1.50956	0.276044	-3.23299	-2.61787	0	0
-9.63518	-6.3703	-7.96934	-8.34082	-2.89962	-0.76939	-3.90255	-3.41327	0	0
-9.6951	-6.46268	-7.64114	-8.14582	-1.11385	0.718343	-3.27419	-2.45471	0	0
-8.18695	-6.5448	-8.1437	-6.59608	-1.28634	0.276044	-3.17118	-2.56688	0	0
-8.49657	-6.48321	-8.65651	-6.78082	-1.45882	0.567559	-3.41841	-2.54649	0	0
-9.87488	-5.89811	-8.65651	-6.66793	-1.76322	0.195626	-3.21239	-2.51589	0	0
-7.48781	-5.87758	-7.89755	-6.19582	-0.80945	0.919387	-2.76944	-2.04681	0	0
-9.62519	-6.55507	-8.63599	-6.41135	-1.72263	0.316253	-3.29479	-2.73004	0	0
-7.77746	-6.3703	-8.33856	-7.10924	-1.6719	0.135312	-3.26389	-2.82182	0	0
-9.84492	-7.366	-8.04113	-5.93924	-0.63697	0.688186	-3.14028	-2.02642	0	0
-9.76502	-7.35574	-8.90266	-9.55187	-2.87932	-0.74929	-4.4485	-3.41327	0	0
-9.01594	-6.53454	-9.04624	-7.27345	-2.04732	-0.27683	-4.06737	-3.18892	0	0
-9.63518	-6.58586	-9.43598	-10.1574	-2.27054	-0.09589	-3.99526	-3.12774	0	0
-5.68603	-7.68958	-7.07064	-6.08438	-0.00601	1.023086	-2.92513	-2.60376	1	1
-8.55584	-6.65963	-8.48765	-8.19206	-1.29151	0.377241	-3.23133	-2.99451	0	0
-9.21569	-6.51401	-9.30265	-8.9053	-3.06196	-0.66887	-3.92315	-3.2705	0	0
-8.36673	-6.84249	-7.63089	-6.36003	-1.89512	0.105155	-3.15058	-2.95438	0	0
-8.12703	-7.00673	-7.79499	-8.51529	-1.63131	0.12526	-3.15058	-2.39352	0	0
-9.96477	-7.37627	-6.46168	-7.0374	-0.50506	0.738447	-2.76944	-1.95504	0	0
-8.81618	-6.91434	-8.97445	-8.06372	-2.32127	-0.29693	-3.69653	-2.98497	0	0
-8.51655	-6.88355	-7.72319	-8.65898	-1.68205	0.155417	-3.45961	-2.69945	0	0
-10.7158	-7.11843	-8.47827	-7.53633	-1.18519	0.595835	-3.6767	-2.78484	0	0
-10.1581	-7.37123	-7.29586	-6.04691	-1.52347	0.019543	-3.57463	-2.93733	0	0
-9.38548	-6.30871	-8.236	-8.50503	-2.20966	-0.35725	-3.65533	-2.91359	0	0
-9.77501	-6.3395	-8.4719	-10.3421	-3.03152	-0.86991	-4.03646	-3.39287	0	0
-9.33555	-7.35574	-8.94368	-7.95082	-0.92107	0.627872	-3.44931	-2.75043	NA	NA
-9.60033	-8.22327	-9.67007	-9.8501	-2.55767	-0.96413	-4.46538	-3.8046	0	0
-5.58462	-7.4555	-7.68061	-6.82441	-0.42162	0.625643	-3.04575	-2.19395	1	1
-10.0247	-7.42759	-8.3078	-7.22214	-0.62682	0.828917	-2.76944	-2.06721	0	0
-8.966	-7.70475	-9.27188	-8.64872	-1.49941	0.21573	-3.4081	-2.61787	0	0
-9.88487	-6.63719	-7.98985	-6.80135	-0.77902	0.758551	-3.07847	-2.37313	0	0
-10.1046	-8.56701	-8.64625	-8.55635	-2.26039	-0.58845	-3.99526	-3.49485	0	0
-6.30926	-8.28985	-8.13344	-7.48898	-1.15443	0.406723	-3.46991	-2.73004	1	1
-8.65638	-7.20176	-6.6668	-6.27793	-1.20516	0.456984	-2.91366	-2.19977	0	0
-8.93604	-7.35574	-10.236	-8.75135	-2.30098	-0.3673	-4.09827	-3.09715	0	0
-10.3044	-7.76634	-9.32316	-6.16503	-1.31678	0.517298	-3.42871	-2.42412	NA	0
-8.56649	-8.34118	-9.97956	-9.56214	-3.39679	-1.24185	-5.03566	-3.99452	0	0
-8.60644	-6.9862	-8.16421	-5.60056	-0.9718	0.658029	-3.19178	-2.32214	0	0
-6.61888	-8.96734	-7.50781	-7.61214	-0.93121	0.175521	-3.61412	-2.62807	1	1
-9.82495	-7.07858	-7.52832	-6.82187	-1.84439	-0.08584	-3.67593	-2.91359	0	0
-7.8274	-8.47462	-7.83601	-6.02135	-0.32243	0.688186	-2.69734	-2.06721	0	0
-7.26808	-7.29415	-7.68217	-5.88792	-0.70799	0.698238	-3.05787	-2.29155	1	1
-8.10965	-6.21021	-7.76507	-5.39118	-1.29151	0.575963	-2.88802	-2.26066	0	0

-9.54529	-7.11964	-7.63089	-7.29398	-0.51521	0.788708	-3.06817	-2.14879	NA	NA
-6.39915	-7.47892	-7.36423	-5.71345	-0.73843	0.537402	-3.04757	-2.36293	NA	0
-7.46784	-6.48321	-9.33342	-5.79556	-1.14429	0.637925	-3.3257	-2.66885	NA	0
-8.36673	-7.05805	-9.1488	-7.29398	-1.47912	0.346409	-3.94376	-2.85241	NA	0
-8.01838	-6.75327	-8.4032	-7.33962	-1.61046	-0.05995	-3.33339	-2.68	NA	0
-10.9136	-6.99646	-9.2206	-8.58714	-2.69669	-0.18636	-4.4073	-3.12774	NA	0
-9.92482	-7.93058	-9.40521	-8.44345	-1.72263	0.03479	-4.04677	-3.23991	0	0
-10.0946	-6.70904	-8.51292	-8.2074	-1.5197	-0.00542	-3.76864	-3.08695	0	0
-9.43808	-6.72518	-7.568	-7.9204	-2.15172	-0.22886	-3.61175	-2.78484	0	0
-9.39751	-6.79072	-9.2947	-7.77989	-1.56214	0.287817	-3.29628	-2.54658	0	0
-8.89048	-6.37874	-8.13105	-7.46139	-1.59113	0.277881	-3.30555	-2.50845	0	0
-7.89731	-7.15044	-8.31805	-6.04187	-0.58623	1.150589	-2.89305	-2.24056	0	0
-11.3231	-6.91434	-8.70779	-6.32924	-1.28634	0.547454	-3.53172	-2.34254	0	0
-8.26686	-6.9862	-8.92317	-8.35108	-1.21531	0.426827	-3.61412	-2.54649	0	0
-10.8781	-6.68772	-8.17798	-7.29278	-2.11306	-0.05001	-3.77876	-3.08028	0	0
-3.39424	-2.71777	-2.55683	-2.62778	-2.52867	-0.95419	0.006895	-0.15442	0	0
-7.47079	-6.93116	-8.32812	-6.06564	-0.77924	0.754812	-2.88802	-2.28925	0	0
-9.41545	-6.80143	-10.5129	-7.53003	-1.3878	0.487141	-3.63473	-2.91359	0	0
-8.16035	-6.71581	-7.90583	-5.81272	-1.66845	0.347433	-3.46329	-2.5847	0	0
-9.30558	-6.97593	-7.87704	-6.42161	-1.42839	0.43688	-3.56262	-2.56688	0	0
-8.12703	-7.15044	-8.44113	-8.05345	-1.74292	0.085051	-3.87165	-3.00537	0	0
-9.27562	-7.12991	-8.06165	-6.44214	-0.99209	0.406723	-3.84075	-2.77083	0	0
-10.9592	-6.85626	-8.64719	-7.42392	-2.05507	-0.08975	-3.63958	-2.85155	0	0
-8.72629	-7.73554	-8.74881	-8.07398	-1.72263	-0.37735	-4.03646	-2.97478	0	0
-7.09558	-8.05474	-8.17798	-7.55507	-0.73091	0.8343	-3.53752	-2.47033	0	0
-9.23567	-7.3968	-8.52318	-7.98161	-2.28068	-0.05568	-4.4279	-3.18892	0	0
-7.56205	-6.97798	-9.85775	-7.99534	-2.98295	-0.81509	-4.1499	-3.83319	1	1
-11.7726	-7.12991	-9.16932	-10.0753	-3.2243	-0.89002	-4.3558	-3.25011	0	0
-8.77893	-6.42556	-8.16859	-6.68389	-1.66845	0.446794	-3.01792	-2.49892	0	0
-6.85858	-7.95111	-6.72834	-4.73845	1.311145	1.763776	-2.19259	-1.24122	0	1
-8.09706	-8.76204	-8.53343	-6.56529	-0.46448	0.52735	-3.23299	-2.40372	1	1
-10.4042	-7.74581	-8.26677	-7.40687	-2.00673	-0.39746	-4.19098	-3.39287	0	0
-9.28561	-6.7501	-8.90266	-7.15029	-1.74292	0.185573	-3.84075	-2.64846	0	0
-8.52654	-7.05805	-6.71808	-5.98029	-0.37316	0.728395	-2.97546	-2.30175	1	1
-8.31679	-7.30441	-8.01037	-7.61214	-1.24575	0.356462	-3.50081	-2.53629	0	0
-9.98475	-6.97593	-9.67187	-8.31003	-1.66175	0.054894	-4.01586	-3.05636	0	0
-8.70632	-7.51998	-8.48215	-8.15608	-1.65161	0.085051	-3.95406	-2.95438	0	0
-10.1346	-7.15044	-8.52318	-7.06819	-1.68205	-0.01547	-3.79954	-2.92379	0	0
-11.6589	-7.84875	-9.59499	-8.78221	-2.8573	-0.68592	-4.56744	-3.50916	0	0
-9.99581	-7.80193	-8.37504	-7.69558	-1.84243	0.267945	-3.70453	-2.59423	0	0
-7.7243	-5.92931	-7.8683	-5.91576	-0.46995	1.380784	-2.55399	-1.62212	0	0
-8.75626	-6.81169	-8.65651	-6.51398	-1.55014	0.21573	-3.53172	-2.69945	0	0
-7.26808	-7.22229	-8.41036	-5.13871	-0.44418	0.577611	-2.77974	-2.24056	0	0
-10.1745	-6.96567	-7.79499	-5.98029	0.215333	1.492365	-2.40891	-1.57773	0	0
-7.93726	-7.12991	-8.57446	-7.02714	-0.55579	0.838969	-3.30509	-2.25076	0	0
-9.15577	-6.27791	-10.1334	-9.31582	-1.30663	0.879178	-3.4081	-2.53629	0	0
-5.6201	-8.11535	-6.46168	-5.07714	0.174747	0.949544	-2.38831	-1.90405	1	1
-6.76869	-8.23853	-8.18472	-7.04766	-0.90077	0.537402	-3.76864	-2.80142	0	0

-10.2045	-6.92461	-8.31805	-6.58582	0.266065	1.592888	-2.65613	-1.95504	0	0
-12.5216	-8.27959	-8.64625	-10.5166	-2.79815	-0.33714	-4.3558	-3.17873	NA	0
-9.36551	-6.92461	-9.48726	-11.5121	-2.58508	-0.43767	-4.04677	-3.09715	0	0
-9.49535	-7.51998	-9.42572	-7.54029	-1.99658	-0.07578	-3.83044	-3.44386	0	0
-9.43542	-7.63289	-9.3847	-7.73529	-2.06761	-0.16626	-4.16008	-3.31129	0	0
-8.28683	-6.70904	-9.60008	-8.68977	-1.75307	0.135312	-3.94376	-3.00537	0	0
-6.55895	-8.34118	-8.8924	-10.1779	-2.81844	-0.92017	-4.34549	-3.43366	0	0
-8.40668	-6.7501	-7.84627	-6.91424	-0.85004	1.029962	-3.08877	-2.27116	0	0
-10.3443	-6.81169	-8.36933	-8.27924	-2.79815	-0.62866	-3.64503	-3.10734	0	0
-8.57648	-7.31468	-9.27188	-8.71029	-2.33142	-0.39746	-3.97466	-3.42346	0	0
-8.78622	-6.79116	-7.17961	-5.63135	0.113869	1.180746	-2.69734	-1.82247	0	0
-7.73444	-7.15588	-8.18736	-6.96492	-0.69225	0.824364	-3.18493	-2.25113	0	0
-8.89048	-7.03416	-8.24366	-8.60422	-1.89076	0.387177	-3.35195	-2.40362	0	0
-9.78286	-8.21391	-7.85891	-8.2951	-2.11306	-0.17918	-3.75093	-2.88014	0	0
-4.93095	-7.69448	-7.43602	-5.74424	-0.42389	0.879178	-3.09907	-2.44451	0	0
-8.88034	-6.82817	-7.29586	-6.7963	-2.44169	-0.30835	-3.75093	-3.03263	0	0
-8.8861	-7.35574	-8.91291	-9.32608	-2.63581	-0.50803	-3.91285	-2.99517	0	0
-8.56649	-7.8074	-9.26162	-7.3145	-1.85453	0.175521	-3.72743	-2.79122	0	0
-6.32923	-7.27362	-7.7437	-6.29845	-0.23111	0.668081	-2.76944	-2.22017	1	1
-9.98475	-6.89381	-9.03599	-7.4274	-1.70234	0.225782	-3.68623	-2.69945	0	0
-9.8437	-7.00607	-8.36566	-10.0093	-2.81864	-1.10323	-3.89938	-3.47103	0	0
-5.41036	-7.63289	-8.83086	-7.43766	-0.33257	0.607768	-3.55232	-2.78103	0	0
-9.13386	-6.23829	-9.67945	-7.40519	-2.02607	0.069223	-3.38906	-3.06122	0	0
-9.98567	-6.85626	-8.17798	-8.3513	-3.19559	-1.04362	-3.91794	-3.50916	0	0
-8.74851	-7.41805	-10.205	-7.19911	-2.83797	-0.46732	-3.59319	-3.69977	0	0
-8.8195	-6.64091	-9.20085	-6.68389	-1.73611	0.039415	-3.23133	-2.76578	0	0
-10.574	-7.32494	-6.55398	-6.66793	-0.36301	0.838969	-2.68704	-1.87346	0	0
-9.41779	-6.93116	-11.8941	-7.78926	-1.92942	0.496474	-4.07568	-3.20418	0	0
-9.59019	-6.60346	-9.21024	-7.8923	-2.77997	-0.74553	-3.83443	-3.23277	0	0
-7.40994	-6.69709	-9.43546	-6.50591	-1.14652	0.804492	-3.56535	-2.61329	NA	0
-7.56205	-6.96862	-7.50231	-6.47781	-1.17552	0.516346	-2.80451	-2.27972	0	0
-10.9236	-6.53454	-11.0154	-8.60766	-1.6719	0.265991	-4.08797	-3.07675	0	0
-7.30854	-6.69709	-7.32401	-4.91344	-0.69225	0.665387	-2.64678	-2.19395	0	0
-8.94118	-6.83753	-8.37504	-9.14754	-2.65432	-0.88464	-3.89938	-3.33761	0	0
-3.65251	-7.66369	-7.55909	-7.16056	-0.27169	1.281268	-3.3154	-2.52609	1	1
-8.29682	-8.22826	-8.26677	-7.47872	-1.1037	0.386618	-3.95406	-3.00537	1	1
-6.61897	-7.92365	-7.83076	-6.92745	-0.50861	0.317625	-3.43545	-2.55611	NA	0
-10.1046	-6.96567	-8.38985	-6.20608	-0.63697	1.050067	-3.18148	-2.17938	0	0
-9.71187	-6.67836	-9.46361	-8.45435	-2.62533	-0.45739	-3.99217	-3.37573	0	0
-10.7938	-7.15044	-11.1488	-7.97135	-1.39795	0.386618	-3.83044	-2.95438	0	0
-8.06909	-7.22142	-8.9381	-7.72368	-1.75544	-0.13943	-3.72309	-3.09934	0	0
-9.89486	-7.28388	-9.43598	-9.17214	-2.19951	-0.64876	-4.45881	-3.54583	0	0
-4.91097	-7.92031	-7.85652	-7.33503	-0.2514	0.808813	-3.06817	-2.37313	0	1
-8.97599	-7.31468	-10.3078	-9.00793	-1.74292	0.235835	-4.22188	-3.20932	0	0
-10.7057	-8.13901	-10.5522	-9.02576	-3.28257	-1.46093	-4.96642	-3.69977	0	0
-8.09706	-7.73554	-7.58986	-6.98608	-1.14429	0.225782	-3.61412	-2.63826	0	0
-9.16575	-7.02726	-9.3847	-6.96556	-1.26604	0.557507	-3.59352	-2.66885	0	0
-10.7259	-7.3525	-9.10701	-7.21784	-1.26251	0.655451	-3.85299	-2.65141	0	0

-4.70123	-8.15641	-7.09756	-6.4524	-0.09921	0.838969	-3.01667	-2.07741	0	0
-9.75503	-7.73554	-7.70268	-5.97003	-1.21531	0.517298	-3.3772	-2.29155	0	0
-8.8702	-6.50046	-9.25716	-7.25531	-2.24838	-0.22886	-3.6303	-2.85155	0	0
-9.25554	-6.43492	-8.90056	-6.66516	-1.99708	-0.05995	-3.47257	-2.97545	0	0
-9.30558	-7.81766	-10.2462	-7.37608	-1.31678	0.135312	-3.97466	-3.62741	0	0
-11.403	-6.93487	-8.8924	-8.34082	-0.94136	0.849022	-3.44931	-2.43431	0	0
-8.24688	-8.44383	-7.34371	-7.75582	-1.59073	-0.3673	-3.28449	-2.98497	1	1
-8.29218	-6.36938	-8.90994	-7.03986	-2.31604	-0.3779	-3.6303	-3.06122	0	0
-8.35303	-6.57537	-8.47827	-6.14995	-1.80377	0.198392	-3.33339	-2.87061	1	1
-4.50147	-8.32065	-7.12833	-6.0624	0.134162	1.20085	-2.85185	-2.24056	1	1
-4.77337	-7.75512	-7.50231	-5.52233	-0.35396	0.585899	-2.99008	-2.54658	0	0
-5.61505	-7.0248	-6.89234	-6.18742	-0.37329	0.903853	-3.06431	-1.87944	0	0
-9.71187	-8.12028	-9.59499	-7.1429	-1.24318	0.297753	-4.12207	-2.94686	1	1
-10.0871	-6.566	-11.0026	-8.39814	-2.51901	-0.24873	-3.77876	-3.147	0	0
-9.0459	-7.27362	-8.46164	-7.72503	-0.49492	0.818865	-3.27419	-2.29155	1	1
-3.41281	-7.94084	-7.73345	-5.53898	0.793678	1.462209	-2.55312	-1.96523	1	1
-9.02593	-6.99646	-8.57446	-7.77635	-0.85004	0.637925	-3.14028	-2.56688	0	0
-7.00432	-6.33193	-8.5815	-6.7682	-1.04021	0.715068	-2.92513	-2.1177	0	0
-5.84982	-7.49945	-8.73856	-8.23819	-1.49941	-0.07578	-3.30509	-2.83201	0	0
-8.36673	-6.84249	-8.54369	-7.79687	-1.31678	0.386618	-3.43901	-2.41392	0	0
-6.91851	-7.83819	-8.91291	-7.16056	-1.33707	0.386618	-3.67593	-2.85241	0	0
-8.85613	-8.13588	-10.6872	-9.63398	-3.87367	-1.43284	-5.22108	-3.83136	0	0
-8.69633	-6.92461	-8.33856	-6.61661	-1.39795	0.12526	-3.3463	-2.35274	0	0
-7.86735	-6.79116	-7.66166	-6.13424	-0.18038	1.301373	-2.82095	-1.84286	0	0
-9.31557	-6.69878	-8.83086	-7.72503	-2.47347	-0.31704	-3.76864	-2.94419	0	0
-6.52899	-6.47295	-8.78984	-8.88477	-0.51521	0.859074	-3.30509	-2.34254	0	0
-11.1733	-7.24282	-8.09242	-6.58582	-0.65726	0.879178	-3.15058	-2.02642	0	0
-11.1234	-7.02726	-8.236	-7.35556	-0.27169	1.261164	-3.16088	-2.02642	0	0
-7.35797	-7.04779	-7.43602	-4.91292	0.458847	1.623045	-2.56342	-1.46556	0	1
-9.81496	-7.79713	-7.88729	-5.71345	-0.1195	1.170694	-3.15058	-1.6899	0	0
-11.6028	-7.20176	-8.24626	-7.34529	-0.31228	0.899283	-3.11968	-2.12839	0	0
-8.24688	-6.65772	-8.53343	-6.8424	-0.60653	0.647977	-3.01667	-2.16918	0	0
-12.3618	-6.49348	-8.16421	-8.31003	-2.53434	-0.29693	-3.99526	-2.93399	0	0
-10.634	-7.64316	-8.00011	-8.37161	-2.91991	-0.92017	-4.3558	-3.45406	0	0
-9.06588	-6.61666	-7.7437	-7.41714	-0.6877	0.859074	-2.87245	-1.95504	0	0
-7.1682	-8.61833	-7.49756	-7.33503	-1.44868	-0.28688	-3.60382	-2.74024	0	0
-6.8486	-7.97164	-6.96423	-5.77503	-1.09355	0.336357	-3.18148	-2.28135	0	0
-11.3031	-7.88952	-8.06165	-10.2908	-3.17357	-0.99054	-4.3764	-3.22971	0	0
-11.8824	-7.62263	-7.69242	-8.05345	-2.04732	-0.19641	-3.58322	-2.76063	0	0
-9.00595	-7.26335	-7.08731	-6.93477	-0.44418	0.577611	-2.82095	-2.03662	0	1
-8.42401	-7.4555	-8.41258	-6.70263	-1.42682	0.188456	-3.32411	-2.29878	0	1
-4.62133	-7.08885	-6.51296	-5.38503	0.722653	1.271216	-2.39861	-1.73069	1	1
-6.519	-7.04779	-7.29243	-6.02135	-0.77902	0.587663	-3.21239	-2.22017	NA	0
-9.58005	-7.28696	-7.69938	-9.55971	-2.36436	-0.24873	-3.65814	-2.73719	0	0
-9.37723	-7.19333	-8.57211	-8.3232	-1.58147	0.158648	-3.6767	-2.65141	0	0
-10.6448	-7.80193	-9.04132	-7.76115	-1.44615	0.12884	-3.82515	-2.8039	0	0
-8.05895	-7.30569	-7.49293	-6.42161	-0.96288	0.595835	-3.40762	-2.26066	0	0
-9.37723	-7.23078	-9.33223	-7.28341	-1.76511	0.317625	-3.74165	-2.81343	0	0

-8.77893	-5.26453	-6.94864	-4.23899	-0.25731	1.271487	-2.27563	-1.66977	0	0
-8.43415	-7.3525	-8.5815	-7.83609	-0.94355	0.953533	-3.3705	-2.35597	0	0
-8.21106	-7.23078	-7.18325	-7.04923	-1.04987	0.50641	-2.77668	-1.8985	0	0
-7.48093	-6.86562	-8.02783	-5.5317	-0.57627	0.854173	-2.62822	-2.09864	NA	0
-12.247	-8.64461	-9.80144	-11.0023	-3.69818	-1.29202	-4.78085	-3.65211	0	0
-5.65561	-7.67085	-8.27182	-6.40287	-0.064	0.973405	-3.12926	-2.12723	1	1
-9.26568	-7.40868	-9.64191	-9.51287	-1.93909	0.337497	-4.02928	-3.00404	0	0
-8.28204	-7.20269	-8.88179	-8.53865	-1.53314	0.337497	-3.62103	-2.69906	0	0
-8.91076	-7.33378	-8.92871	-7.59254	-0.82757	0.943597	-3.2406	-2.35597	0	0
-6.20321	-8.16709	-6.6765	-6.05627	0.148636	1.261551	-2.80451	-2.07005	1	1
-9.92482	-7.87925	-9.23085	-7.63266	-1.58058	-0.08584	-4.10857	-2.97478	0	0
-7.48781	-6.86302	-7.85652	-7.19135	-1.91541	-0.23662	-3.76864	-2.75043	0	0
-7.94725	-7.49945	-8.60523	-8.0124	-1.1037	0.336357	-3.74804	-2.91359	0	0
-9.35695	-7.75512	-9.56684	-8.12648	-1.64912	0.575963	-3.85299	-2.93733	0	0
-9.31639	-6.94053	-7.40847	-6.93682	-1.49448	0.307689	-3.19421	-2.84202	0	0
-9.83493	-6.87328	-9.21034	-8.51529	-2.74742	-0.73923	-4.26309	-3.44386	NA	0
-9.89486	-9.33688	-9.48726	-10.4858	-3.89396	-1.63388	-5.02536	-3.98432	0	0
-9.03245	-7.69894	-7.09879	-6.14995	-1.81344	-0.06988	-3.09215	-2.3655	0	0
-9.57525	-7.55077	-8.20523	-6.73977	-1.24575	0.336357	-3.3463	-2.56688	0	0
-9.06588	-8.14614	-8.22575	-6.38056	-0.52536	0.547454	-3.63473	-2.79122	0	0
-10.2844	-7.366	-9.45649	-8.09451	-1.04282	0.597716	-3.55232	-2.62807	0	0
-8.72823	-7.07161	-8.73164	-6.47781	-1.77477	-0.06988	-3.3705	-2.93733	0	0
-7.92727	-8.60807	-7.52832	-6.04187	0.083429	0.70829	-2.74884	-1.81227	1	1
-9.76258	-7.44614	-9.56684	-8.06091	-1.7941	0.486538	-3.72309	-2.9278	1	1
-9.70509	-7.70475	-9.83597	-6.70898	-0.70799	1.009858	-3.52142	-2.45471	0	0
-9.71187	-7.07161	-9.16332	-7.40519	-2.69299	-0.45739	-3.76948	-3.30902	0	0
-9.36551	-6.93487	-8.51292	-8.41266	-1.11385	0.547454	-3.46991	-2.60767	0	0
-8.69633	-6.88355	-8.43087	-7.54029	-0.82975	0.849022	-3.3257	-2.37313	0	0
-9.68145	-7.32441	-8.20613	-5.9626	0.593244	1.420528	-2.39625	-1.48869	0	0
-9.33555	-8.76204	-6.65655	-5.70319	-0.38331	0.366514	-2.89305	-2.04681	0	0
-8.61643	-7.14017	-9.72315	-9.04898	-2.14878	-0.3472	-4.27339	-3.18892	0	0
-7.74749	-6.45242	-9.3129	-7.30424	-1.55014	0.336357	-3.56262	-2.58728	0	0
-10.3843	-8.46436	-10.7283	-8.13556	-1.71249	-0.3472	-4.03646	-3.57642	0	0
-5.69002	-7.64316	-9.5488	-6.17529	-0.89063	0.557507	-3.62443	-2.59747	0	0
-6.61888	-7.99217	-7.60012	-6.4524	-0.44418	0.869126	-3.22269	-2.33234	1	1
-6.28433	-8.10155	-8.43135	-8.67916	-1.78444	0.029479	-3.84371	-3.09934	0	0
-8.19077	-7.50231	-8.90994	-6.95555	-1.5718	0.297753	-3.71381	-2.91827	0	0
-8.01838	-8.22327	-7.3897	-7.02112	-0.83723	0.238136	-2.99008	-2.51798	1	1
-5.96997	-7.54913	-7.22079	-5.10079	0.438598	1.162191	-2.5076	-2.05099	1	1
-5.01675	-7.17461	-8.11229	-5.74715	-0.48928	0.566027	-2.66533	-2.54658	0	0
-7.18685	-7.40868	-8.21551	-6.84314	-0.86623	0.655451	-3.13854	-2.51798	0	0
-5.65561	-7.89556	-7.71815	-5.90639	-0.30564	0.436858	-2.86946	-2.41315	1	1
-10.5941	-7.57722	-8.79733	-9.38172	-1.99708	-0.42758	-4.10351	-3.04216	0	0
-11.0504	-8.02665	-10.0079	-10.3934	-2.62533	-0.71573	-4.31692	-3.22324	0	0
-10.6752	-7.07161	-9.5293	-7.24594	-1.58147	0.387177	-3.55608	-2.47986	0	0
-5.41223	-7.48359	-8.39381	-6.99302	-0.52794	0.923725	-3.20349	-2.43221	1	1
-9.43808	-7.46486	-9.05071	-6.58085	-1.11753	0.665387	-3.45401	-2.34644	0	0
-8.79921	-7.3525	-9.50115	-7.88293	-1.33017	0.387177	-3.66742	-2.83249	0	0

-9.5192	-7.2776	-9.95159	-9.80326	-2.548	-0.32822	-4.00145	-3.18512	1	1
-8.93104	-7.41805	-9.77329	-8.74474	-1.13686	0.655451	-3.60247	-2.78484	0	0
-9.36709	-7.06225	-7.82138	-7.25531	-0.83723	0.794556	-3.11998	-2.12723	0	0
-9.20484	-7.83002	-8.8161	-7.64874	-0.97255	0.526282	-3.52824	-2.59423	1	1
-8.52542	-7.30569	-9.60438	-7.31151	-1.12719	0.436858	-3.79732	-2.81343	0	0
-7.49107	-7.68958	-7.89645	-7.10543	-0.48928	0.883981	-3.40762	-2.3655	0	0
-8.36317	-7.12779	-8.14044	-6.26236	-0.94355	0.476602	-3.56535	-2.5847	0	0
-9.49892	-10.5266	-8.46889	-8.57612	-1.39782	-0.41764	-3.92722	-3.00404	1	1
-10.7868	-7.37123	-10.3457	-9.19438	-2.19039	-0.10963	-4.44682	-3.17559	0	0
-9.29611	-7.26824	-9.06009	-8.59486	-1.66845	-0.01027	-3.57463	-2.90873	0	0
-7.80543	-7.83002	-8.90056	-6.93682	-0.8179	0.943597	-3.40762	-2.62282	0	0
-8.37331	-8.36372	-8.01844	-6.58085	-1.23351	0.317625	-3.65814	-2.8039	0	0
-11.233	-8.47608	-8.46889	-10.4215	-3.18592	-0.96413	-4.62311	-3.5854	0	0
-7.77501	-7.8113	-7.31463	-5.46612	0.303283	1.420528	-2.93441	-2.04146	1	1
-10.4623	-8.0641	-8.97563	-7.20847	-1.62979	0.297753	-3.83443	-2.61329	1	1
-7.8967	-7.94238	-8.48765	-7.44266	-0.30564	1.271487	-3.05503	-2.27972	1	1
-9.21498	-6.96862	-7.87768	-7.36772	-0.75991	1.082702	-3.1571	-1.98428	0	0
-10.2493	-7.66149	-9.90467	-7.38645	-1.0692	0.814428	-3.65814	-2.76578	0	0
-9.00203	-7.20269	-8.14982	-6.64642	-0.52794	0.774684	-2.81379	-1.88897	0	0
-8.51528	-7.13715	-8.92871	-8.37941	-1.33017	0.486538	-3.4169	-2.52752	0	0
-9.52934	-7.37123	-9.28531	-8.42624	-1.30117	0.585899	-3.53752	-2.47033	NA	0
-9.73216	-7.65212	-9.473	-8.51992	-1.53314	0.416986	-3.8066	-2.71812	0	0
-10.1046	-7.06832	-8.65651	-8.22793	-1.76322	-0.00542	-4.04677	-2.99517	0	0
-5.7	-6.99646	-8.41036	-7.37608	-2.20966	-0.44772	-4.03646	-3.31129	0	0
-6.76869	-7.65342	-8.59497	-7.25293	-2.19951	-0.35725	-3.94376	-3.23991	0	0
-7.26808	-7.71501	-7.83601	-6.62687	-1.31678	0.43688	-3.336	-2.95438	0	0
-9.71187	-8.07346	-8.85364	-7.42392	-1.26251	0.138776	-3.64886	-2.71812	0	0
-9.30558	-7.61236	-8.21549	-5.84687	-0.82975	0.768604	-3.16088	-2.64846	0	0
-6.82862	-8.22826	-7.56935	-6.68845	-1.49941	-0.25673	-3.63473	-3.03596	0	0
-5.76992	-8.87496	-6.4104	-4.36898	0.316797	1.241059	-2.51192	-1.87346	0	0
-7.73751	-7.06832	-8.35908	-7.24266	-1.57044	-0.06573	-3.3669	-2.78103	0	0
-4.04204	-7.03752	-7.44627	-5.69292	0.286358	1.20085	-2.46041	-2.17938	0	0
-9.32653	-7.61467	-9.53869	-8.22016	-1.59113	0.2282	-3.92722	-2.73719	0	0
-5.20062	-6.63719	-6.93346	-7.05793	-1.35736	0.517298	-3.02697	-2.32214	0	0
-9.70173	-7.03416	-7.75569	-9.00703	-1.14652	0.516346	-3.59319	-2.35597	0	0
-9.23567	-6.80143	-8.66676	-7.87898	-1.61102	-0.03558	-3.80984	-3.16853	1	1
-10.1945	-7.5713	-8.34882	-7.76608	-1.49941	0.145364	-3.86135	-3.01557	0	0
-6.57893	-7.1607	-7.87704	-6.73977	-0.74858	0.758551	-2.87245	-1.95504	0	0
-8.46457	-7.07161	-7.22079	-6.7963	-1.13686	0.436858	-3.29628	-2.56564	0	0
-10.3044	-6.72957	-8.44113	-7.77635	-2.31112	-0.26678	-3.90255	-2.96458	0	0
-8.03714	-7.86899	-9.06676	-7.09898	-1.11385	0.497193	-3.72743	-3.01557	0	0
-9.24566	-6.85275	-7.82575	-5.89819	-1.08341	0.477089	-3.17118	-2.28135	0	0
-8.76624	-7.28388	-8.68728	-7.12977	-1.65161	0.346409	-3.75834	-3.17873	0	0
-9.96477	-7.1607	-7.77447	-7.30424	-1.26604	0.61782	-3.63473	-2.63826	0	0
-9.09584	-7.21203	-7.46679	-7.00661	-0.27169	1.11038	-3.03727	-2.0876	0	0
-11.1034	-6.91434	-8.49241	-8.9874	-2.1082	-0.02552	-4.01586	-3.02576	0	0
-10.7039	-7.11964	-9.74366	-9.26451	-2.79815	-0.3874	-4.4485	-3.34188	0	0
-9.72201	-6.91244	-7.73692	-6.93682	-1.75544	-0.19905	-3.44473	-2.74672	0	0

-7.38793	-7.02726	-7.8155	-5.35424	-0.00789	1.231007	-2.87245	-2.17938		0	0
-10.9935	-6.82196	-9.42572	-9.15161	-1.62117	0.386618	-3.52142	-2.97478		0	0
-5.44266	-7.14652	-5.63485	-4.27646	0.544917	1.410592	-1.71892	-1.49822		1	1
-8.42666	-7.54051	-7.76422	-5.39529	0.002258	0.989753	-2.61493	-2.04681		1	1
-7.79743	-7.73554	-7.72319	-5.89819	-0.39345	1.009858	-2.97546	-2.0876		0	0
-8.74627	-7.37627	-8.57446	-8.51529	-1.59073	-0.08584	-3.88195	-2.96458		0	0
-9.25564	-7.28388	-7.39499	-6.55503	0.205186	1.472261	-2.65613	-1.86326		0	0
-9.89486	-8.02296	-8.32831	-9.17214	-2.53434	-0.54824	-4.09827	-3.2705		0	0
-7.86735	-7.3968	-8.61548	-9.14135	-2.20966	-0.44772	-4.07767	-3.56623		1	1
-9.22568	-7.04779	-8.64625	-7.01687	-1.40809	0.446932	-3.3154	-2.64846		1	1
-7.64318	-7.67085	-7.27709	-5.36308	-0.28631	0.595835	-2.74884	-1.95569		1	1
-7.42788	-7.64316	-8.0719	-6.26766	-0.60653	0.43688	-2.81065	-2.36293	NA		0
-9.10583	-6.71931	-9.32316	-7.18108	-2.51405	-0.31704	-4.01586	-2.84221		1	1
-9.49535	-6.1342	-9.34367	-6.82187	-2.07776	-0.10594	-3.84075	-2.86261		0	0
-8.52654	-6.7809	-8.99496	-7.22214	-1.09355	0.778656	-3.61412	-2.4853		0	0
-7.20815	-8.32065	-7.70268	-5.69292	-0.41374	0.356462	-3.336	-2.55668		1	1
-7.61276	-7.7083	-8.45012	-7.61127	-0.87589	0.148712	-3.37978	-2.4608		0	0
-9.31557	-6.91434	-9.70264	-8.57687	-1.41824	0.135312	-3.88195	-3.23991		0	0
-11.5329	-7.09911	-9.3847	-7.05793	-1.33707	0.557507	-3.55232	-2.883		0	0
-8.44664	-7.63289	-10.5949	-8.43319	-2.04732	-0.35725	-4.18068	-3.6682		0	0
-9.60521	-6.89381	-7.76422	-5.92898	-0.70799	0.989753	-2.86215	-2.01622		0	0
-4.69124	-9.16238	-7.57961	-6.54477	-1.41824	-0.84981	-3.60382	-3.21952		1	1
-13.3107	-7.47892	-8.76933	-9.56214	-3.14313	-1.0408	-4.3558	-3.34188		0	0
-7.55773	-6.5448	-8.38985	-7.72503	-0.92107	0.497193	-3.23299	-2.33234		0	0
-6.25932	-6.72957	-7.6514	-5.38503	-0.13979	0.889231	-2.91366	-2.11819		0	0
-8.0671	-7.71501	-9.70264	-9.40819	-2.02702	0.054894	-4.01586	-2.84221		0	0
-8.2212	-6.11657	-6.70465	-4.7823	-0.49894	-0.06988	-1.79315	-2.53705		0	0
-10.584	-6.78135	-8.79733	-7.31151	-1.0982	0.685259	-3.20349	-2.26066		0	0
-8.65725	-7.21206	-7.39909	-6.21552	-1.01121	0.774684	-3.16638	-2.10817		0	0
-11.6386	-6.51919	-9.7076	-10.4496	-3.81417	-0.99394	-4.35403	-2.5847		0	0
-10.0972	-6.91244	-9.06009	-8.43561	-1.51381	0.208328	-3.56535	-2.68953		0	0
-8.2212	-8.14837	-7.59615	-7.32088	-0.53761	0.516346	-3.00864	-2.27972		1	1
-9.31639	-6.70645	-7.65246	-7.5457	-1.21418	0.486538	-3.25916	-2.43221		0	0
-8.26176	-6.57537	-7.53985	-5.75652	0.167967	1.241679	-2.77668	-1.86991		0	0
-8.26176	-6.82817	-7.53985	-8.00471	-1.41716	0.238136	-3.20349	-2.47033		0	0
-8.11979	-6.8469	-8.77857	-6.97429	-0.25731	1.211871	-3.07359	-2.23207		1	1

culture_or_	tb_diag_treat_prevto6_lab
0	0
1	1
0	0
0	0
0	0
0	0
1	1
0	0
0	0
0	0
0	0
0	0
0	0
0	0
1	1
0	0
0	0
0	0
0	0
0	0
0	0
0	0
1	1
1	1
0	0
0	0
0	0
1	0
1	1
0	0
0	0
0	0
0	0
0	0
0	0
0	0
0	0
0	0
1	0
0	0
0	0
0	0
1	1
0	0
0	0
0	0
1	1

1	1
0	1
0	0
0	0
0	0
0	0
1	1
0	0
1	1
0	0
0	0
0	0
1	1
0	1
0	0
0	0
0	0
0	0
0	0
0	0
0	0
0	0
0	0
0	0
0	0
0	0
0	0
0	0
0	0
0	0
0	0
0	0
0	0
0	0
0	0
0	0
0	0
0	0
0	0
0	0
0	0
0	0
0	0
0	0
0	0
0	0
NA	0
0	0

0	0
0	0
0	0
0	0
1	1
1	1
1	1
0	0
1	1
0	0
0	0
0	0
0	0
0	0
0	0
0	0
0	0
0	0
0	0
0	1
0	1
0	1
0	0
0	0
0	0
0	0
0	0
0	0
0	0
0	0
0	1
1	1
1	1
0	0
0	0
0	0
1	1
1	0
0	0
1	1
0	1
0	0
0	0
0	0
0	0
0	0
0	0
0	0

0	0
0	0
1	1
1	1
0	0
0	0
0	0
0	0
0	0
0	0
0	0
0	0
NA	1
NA	0
0	0
0	0
0	0
0	0
0	0
0	0
0	0
0	0
0	0
0	0
0	0
0	0
1	1
0	0
0	0
0	0
0	0
0	0
0	0
0	0
0	0
0	0
0	1
0	0
0	0
0	0
0	0
0	0
0	0
0	0
0	0
0	0
0	0
0	0

0	1
0	0
0	0
0	0
0	0
0	0
1	1
1	1
0	0
0	0
0	0
0	0
0	0
0	0
0	1
0	0
0	0
0	0
0	0
0	1
0	0
0	0
0	0
0	0
0	0
1	1
0	0
0	0
0	0
1	1
0	0
0	0
1	1
0	0
0	0
0	0
0	0
0	1
0	1
0	0
0	0
0	0
0	0
0	0
0	0
0	0
0	0
1	1

0	0
0	0
0	1
0	0
0	0
0	0
0	0
0	0
0	0
0	0
0	1
0	0
0	0
0	0
0	0
0	0
0	0
0	0
0	0
0	0
0	0
0	0
0	0
0	0
0	0
0	0
0	0
0	0
0	0
0	0
0	0
0	0
1	1
0	0
0	0
0	0
0	0
0	0
0	0
0	0
1	1
0	0
1	1
0	0
1	1
0	0
0	0
0	0

	1	0
NA		0
	0	0
	0	0
	0	0
	0	0
	0	0
	0	0
	1	1
	1	1
	0	0
	0	0
	0	0
	0	0
	0	0
	0	0
	0	0
	1	1
	0	0
	0	0
NA		1
	0	0
	0	0
	0	0
	0	0
	0	0
	0	0
	0	0
NA		1
	0	0
	0	0
	0	0
	1	1
	0	0
	0	0
	0	0
	0	0
	0	0
	0	0
	0	0
	0	0
	0	0
	0	0
	0	0
	0	1
	0	0
	1	1
	0	0

0	0
0	1
0	0
1	1
0	0
0	0
0	0
0	0
0	0
0	0
0	0
0	0
0	0
0	0
0	0
0	0
0	0
0	0
0	0
0	0
0	0
0	0
0	0
0	0
0	0
0	0
0	0
0	0
0	0
1	1
1	1
0	0
0	0
0	0
0	0
0	0
0	0
0	0
1	1
0	0
0	0
0	0
0	0
0	0
0	0
0	0
0	0
0	0

	0	0
	0	0
NA		0
	0	0
	0	0
	0	0
	0	0
	0	0
	0	0
	0	0
	0	0
	0	0
	0	0
	0	0
	0	0
	0	0
	0	0
	0	0
	1	1
	0	0
	0	0
	0	1
	0	0
	0	0
	0	0
	0	0
	0	0
	0	0
	0	0
	0	0
NA		0
	0	0
	1	1
	0	0
	0	0
	0	0
	0	1
	1	1
	0	0
	0	0
	0	0
	0	0
	0	0
	1	1
	0	0
	0	0
	1	1
	0	0

NA	0
0	0
0	0
0	0
0	1
0	0
0	0
0	0
0	0
0	0
0	0
0	0
0	0
0	0
0	0
0	0
0	0
0	0
0	0
0	0
0	0
0	0
0	0
0	0
0	1
0	0
1	1
0	0
0	0
0	1
1	1
0	1
0	0
1	1
0	0
0	0
0	0
0	0
0	0
0	0
0	0
0	0
0	0
0	0
0	0
0	0
1	1
0	1

0	0
0	0
0	0
0	0
0	0
0	0
0	0
0	0
0	0
0	0
0	0
0	0
0	0
0	0
0	1
0	0
0	0
0	0
1	1
0	0
0	0
0	0
0	0
0	0
0	0
0	0
0	0
0	0
0	0
0	0
0	1
0	0
0	0
0	0
1	1
1	1
0	0
0	0
0	0
0	0
0	0
0	0
0	1
0	1
0	0
0	0
0	0
0	0

0	0
0	0
0	0
0	0
0	0
0	0
1	1
0	0
1	1
1	1
0	0
0	1
1	1
0	0
1	1
1	1
0	0
0	0
0	0
0	0
0	0
0	0
0	0
0	0
0	1
0	0
0	0
0	0
1	1
0	0
0	0
0	0
0	0
0	0
0	0
0	0
0	1
0	0
0	0
1	1
1	1
1	1
0	0
0	0
0	0
0	0
0	0
0	0

0	0
0	0
0	0
0	1
0	0
1	1
0	0
0	0
0	0
1	1
0	0
0	0
0	0
0	0
0	0
0	0
0	0
0	0
0	0
0	0
0	0
0	0
1	1
1	1
0	0
0	0
0	0
0	0
0	0
0	0
0	0
0	0
0	0
0	0
1	1
0	0
0	0
1	1
1	1
0	1
0	0
1	1
0	0
0	0
0	0
1	1
0	0
0	0

1	1
0	0
0	0
1	1
0	0
0	0
0	0
1	1
0	0
0	0
0	0
0	0
0	0
1	1
1	1
1	1
0	0
0	0
0	0
0	0
0	1
0	0
0	0
0	0
0	0
0	0
0	0
0	0
0	0
0	0
0	0
0	0
0	0
0	1
0	0
1	1
0	0
0	0
0	0
0	0
0	0
0	0
0	0
0	0
0	0
0	0
0	0
0	0

0	0
0	0
1	1
1	1
0	0
0	0
0	0
0	0
1	1
1	1
1	1
0	0
1	1
0	1
0	0
1	1
0	0
0	0
0	0
0	0
0	0
1	1
0	0
0	0
0	0
0	0
0	0
0	1
0	0
0	0
0	0
1	1
0	0
0	0
0	0
1	1
